# Genome-Wide Identification and Expression Profiling of Aconitase Gene Family Members Reveals Their Roles in Plant Development and Adaptation to Diverse Stress in *Triticum aestivum* L.

**DOI:** 10.3390/plants11243475

**Published:** 2022-12-12

**Authors:** Mahipal Singh Kesawat, Bhagwat Singh Kherawat, Chet Ram, Anupama Singh, Prajjal Dey, Jagan Singh Gora, Namrata Misra, Sang-Min Chung, Manu Kumar

**Affiliations:** 1 Department of Genetics and Plant Breeding, Faculty of Agriculture, Sri Sri University, Cuttack 754006, India; 2 Krishi Vigyan Kendra, Bikaner II, Swami Keshwanand Rajasthan Agricultural University, Bikaner 334603, India; 3 ICAR-Central Institute for Arid Horticulture, Bikaner 334006, India; 4 KIIT-Technology Business Incubator (KIIT-TBI), Kalinga Institute of Industrial Technology 13 (KIIT), Deemed to be University, Bhubaneswar 751024, India; 5 Department of Life Science, Dongguk University, Dong-gu 10326, Republic of Korea

**Keywords:** aconitase, biotic stress, cis-acting regulatory elements, cold stress, drought stress, heat stress, qRT-PCR

## Abstract

Global warming is a serious threat to food security and severely affects plant growth, developmental processes, and, eventually, crop productivity. Respiratory metabolism plays a critical role in the adaptation of diverse stress in plants. Aconitase (ACO) is the main enzyme, which catalyzes the revocable isomerization of citrate to isocitrate in the Krebs cycle. The function of ACO gene family members has been extensively studied in model plants, for instance Arabidopsis. However, their role in plant developmental processes and various stress conditions largely remained unknown in other plant species. Thus, we identified 15 ACO genes in wheat to elucidate their function in plant developmental processes and different stress environments. The phylogenetic tree revealed that *TaACO* genes were classified into six groups. Further, gene structure analysis of *TaACOs* has shown a distinctive evolutionary path. Synteny analysis showed the 84 orthologous gene pairs in *Brachypodium distachyon, Aegilops tauschii, Triticum dicoccoides, Oryza sativa*, and *Arabidopsis thaliana*. Furthermore, Ka/Ks ratio revealed that most *TaACO* genes experienced strong purifying selection during evolution. Numerous cis-acting regulatory elements were detected in the *TaACO* promoters, which play a crucial role in plant development processes, phytohormone signaling, and are related to defense and stress. To understand the function of *TaACO* genes, the expression profiling of *TaACO* genes were investigated in different tissues, developmental stages, and stress conditions. The transcript per million values of *TaACO*s genes were retrieved from the Wheat Expression Browser Database. We noticed the differential expression of the *TaACO* genes in different tissues and various stress conditions. Moreover, gene ontology analysis has shown enrichment in the tricarboxylic acid metabolic process (GO:0072350), citrate metabolic process (GO:0006101), isocitrate metabolic process GO:0006102, carbohydrate metabolic (GO:0005975), and glyoxylate metabolic process (GO:0046487). Therefore, this study provided valuable insight into the ACO gene family in wheat and contributed to the further functional characterization of *TaACO* during different plant development processes and various stress conditions.

## 1. Introduction

The Krebs cycle, or citric acid cycle, is also known as the Tricarboxylic acid (TCA) cycle [1]. The TCA cycle produces ATP in almost all living organisms, which is a vital source of energy [2]. The TCA cycle is the principal component of carbon metabolism that supplies the electrons for oxidative phosphorylation, and also acts as an intermediate for amino acid biosynthesis. Further, the TCA cycle connects glycolysis to fatty acid metabolism through the glyoxylate cycle [3]. The reversible isomerization of citrate to isocitrate is the second step of the TCA cycle, which is catalyzed by aconitase gene family members for instance, via cis-aconitate [4]. Aconitase (ACO) is also recognized as aconitate hydratase (EC 4.2.1.3), which contains an iron–sulfur protein (4Fe–4S) with a molecular weight of ~90 kDa [5,6,7]. Generally, Aconitase has two isoforms, including the mitochondrial isoform and cytosolic aconitase. The mammalian aconitases are extensively studied, which expand our understanding and knowledge about the functions and underlying mechanisms of this gene family member [8,9]. ACO1 (cytosolic aconitase) contains the AcnA_IRP domain, binds to RNA stem-loop structure, and hinders the translation of the ferritin transcript in humans [10], while ACO2 contains the AcnA_mitochondrial domain and functions in the TCA cycle. 

Remarkably, all the identified aconitase genes in higher plants shared higher sequence similarity with the cytosolic aconitase, whereas animal aconitase genes have shown sequence similarity with mitochondrial isoforms [11,12,13]. *Arabidopsis* genome encodes the three aconitase genes and possesses the AcnA_IRP domain as human, however, no iron-responsive element-binding protein 1 (IRP1) activity was observed in the *Arabidopsis* [13]. All three AtACO were found in the proteome of mitochondria [14,15]. However, the ACO 1 gene localized in mitochondria and cytosol in wild species of tomato [16]. Plant aconitases have been implicated in carbon, sucrose, lipid metabolism, and the glyoxylate cycle [17,18,19]. Plant aconitases have also participated in a wide range of developmental processes, such as programmed cell death, oxidative stress, and hypersensitive response [11,12,13,20]. ACOs gene family members were only reported in some model plants including *Arabidopsis*, maize, soybean, sorghum, poplar, and physcomitrella [1,12,21]. However, the role of ACO gene family members largely remain unexplored in other plant species. The ACO gene family has been extensively studied in *Arabidopsis* [4,12]. AtACO3 regulates the stress response via phosphorylation at Ser91 that is prompted by mitochondrial dysfunction. Further, proteomic data showed phosphorylation and abundance of AtACO3 increased under stress, which required signaling via ANAC017. AtACO3 enhanced the tolerance against ultraviolet-B- and antimycin-A-stimulated mitochondrial dysfunction in *Arabidopsis*. These results suggest that AtACO3 is a target, and mediated signaling of mitochondrial dysfunction, which is crucial for accomplishing plants stress tolerance [22]. AtACO3 also functions in the first 3 days during germination in *Arabidopsis*. The expression and activities of ZmAco1 and ZMAco4 were increased in scutellum during germination in maize. The activity of mitochondrial and cytosolic forms of aconitase was highest on the fourth day of germination. However, the expression and activity of the cytosolic form suddenly declined, whereas the mitochondrial form reduced more slowly. Further, the activity of the mitochondrial form ACO was strongly inhibited by H_2_O_2_ compared to the cytosolic form. Thus, these results indicate that the mitochondrial form acts in the TCA cycle, while the cytosolic form operates in the glyoxylate [23]. Moreover, the expression of cytosolic and mitochondrial forms of aconitase is regulated by phytochrome in maize leaves [24]. Aconitase also acts as a biosensor for oxidants in plants. Oxidative stress in *Arabidopsis* cells, resulting in the degradation of a special type of mitochondrial protein, such as aconitase, led to the effect on respiratory functions [25]

With the recent advances in DNA sequencing technology, there has been a rapid increase sequenced in plant genomes in the past decade. However, the genes identified in plant genomes is now a great challenge for plant biologists, especially their structural and functional characterization [7,26,27,28]. The long-awaited wheat (*Triticum aestivum* L.) genome was recently completed and the ~124,201 gene loci were annotated [29,30]. Wheat is a globally major cereal crop and provides half of the food eaten by the world’s population [29,31,32]. India is the second-largest wheat-producing country [33]. Wheat is an important source of protein, carbohydrates, vitamins, and minerals [26,27,33]. Further, the global demand for wheat has been rising and we will need to increase wheat production to feed the ever-growing world population [34]. However, wheat production is severely affected by diverse stresses [35,36,37]. Thus, publicly available genome sequence data provide the opportunity to carry out a genome-wide analysis of the ACO gene family in wheat. Here, we identified and analyzed the ACO gene family of wheat using different computational tools. The analysis of the *TaACO* gene family includes phylogenetic analysis, chromosomal distribution, gene structure, synteny relationship, and protein–protein interaction. Further, the expression profiling of *TaACO* genes were investigated in different tissues, developmental stages, and stress conditions. The expression profile of *TaACO* genes revealed the differential expression pattern of the *TaACO* genes in different tissues and various stress conditions. Therefore, our results provide valuable information about the ACO gene family in wheat and contribute to the further characterization of ACOs during plant development and response to diverse biotic and abiotic stress. 

## 2. Results

### 2.1. Genome-Wide Characterization and Evolutionary Analysis of TaACO Genes

In the present investigation, 15 ACO genes were identified in the wheat genome by using different computational tools (Table 1; Table S1).

Compared to other plants, this number is relatively higher than, for instance, *A.thaliana, O.sativa*, *G.max*, *B. distachyon* and *S. bicolor* (Table S2). 

This may be due to the fact that wheat is hexaploid with a bulky genome size. *TaACO* gene family members contain protein lengths ranging from 477–1007 amino acids with molecular weights 51.92–109.32 kilodalton for TaACO10 and TaACO2. The isoelectric point was 5.67 and 6.6 for TaACO14 and TaACO7. We also correlated the molecular weight of TaACOs with their isoelectric point values to understand the dispersal of various TaACOs (Figure S1). These results indicate that all TaACOs had a similar isoelectric points and molecular weights. The GRAVY values ranged from −0.073 to −0.227, suggesting that TaACO proteins are hydrophilic. Further, we also predicted the subcellular location of TaACO proteins; most of the TaACOs localized at chloroplast thylakoid membrane, while two TaACOs were situated in the mitochondrion, cytoplasm, and one TaACO protein was found at extracellular space (Table 1).

**Figure 1:**
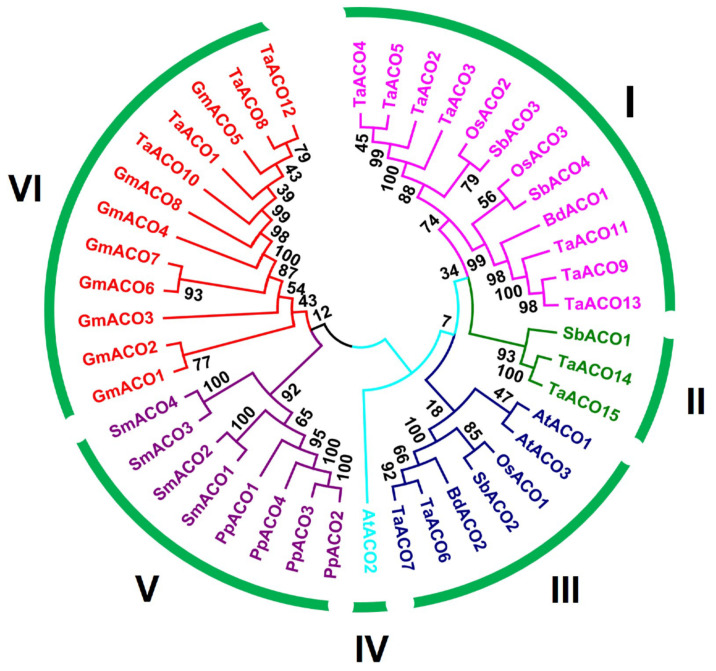
Phylogenetic analysis of ACO proteins among the rice (3) wheat (15), Arabidopsis (3), soybean (8), Brachypodium (2), Sorghum (4), Physcomitrella (4), and Selaginella (4) using the MEGAX by the neighbor-joining (NJ) method and the bootstrap was 1000 repeats.

Furthermore, to investigate the evolutionary dynamics between TaACO and other crop ACOs, the phylogenetic tree was generated with *Arabidopsis thaliana* (AtACOs), *Oryza sativa* (OsACOs), *Glycine max* (GmACOs), *Sorghum bicolor* (SbACOs), *Brachypodium distachyon* (BdACOs), *Physcomitrella patens* (PpACOs), and *Selaginella moellendorffii* (SmACOs) proteins (Table S3). The phylogenetic tree has shown that TaACO proteins were separated into six groups (Figure 1). 

Group I comprises seven members whereas, Group II, III, IV, V, and VI contain two, two, zero, zero, and four members, respectively (Figure S2).

### 2.2. Chromosomal Location, Gene Duplication and Synteny Relationship of TaACO Genes 

**Figure 2:**
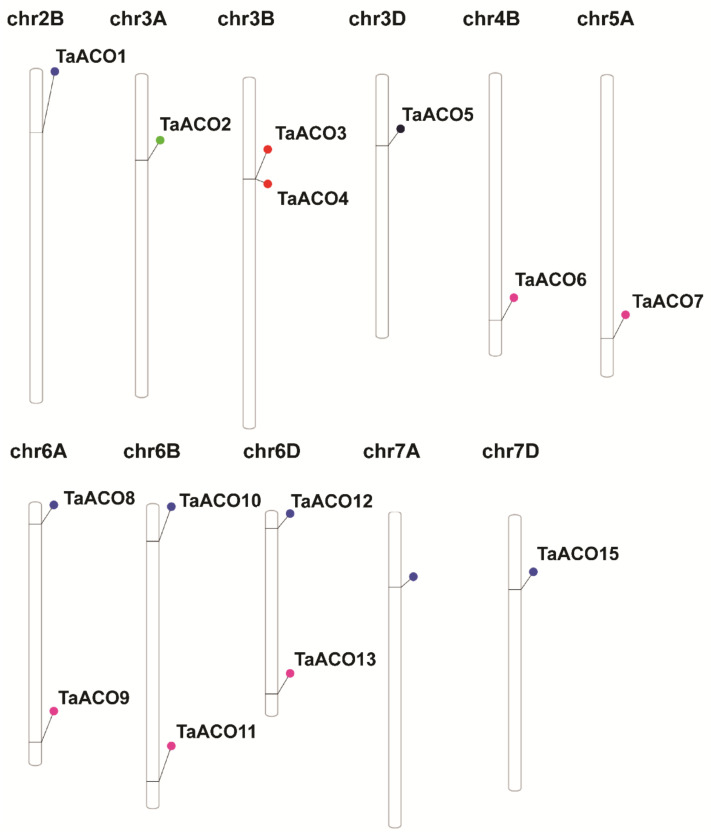
Chromosomal locations of identified ACO genes on the three sub-genomes of wheat. Schematic chromosomal location of ACO genes on the different chromosomes, whereas gene name is mentioned on the right side. The different colored sphere indicates the position of the ACO genes on the wheat chromosomes. The chromosome numbers are shown above the chromosomes.

The identified *TaACO* genes were mapped on the wheat chromosome using the Phengram webserver. The *TaACO* genes were located on 11 chromosomes of wheat (Figure 2 and Table 1). The B sub-genomes contain the maximum *TaACO* genes (6), followed by A sub-genomes (5) and D sub-genomes (4), respectively (Figure S3A). Further, a single gene was found on the chromosomes 2B, 3A, 3D, 4B, 5A, 7A, and 7D, while two *TaACO* genes were mapped on the chromosomes 3B, 6A, 6B, and 6D (Figure S3B).

**Figure 3:**
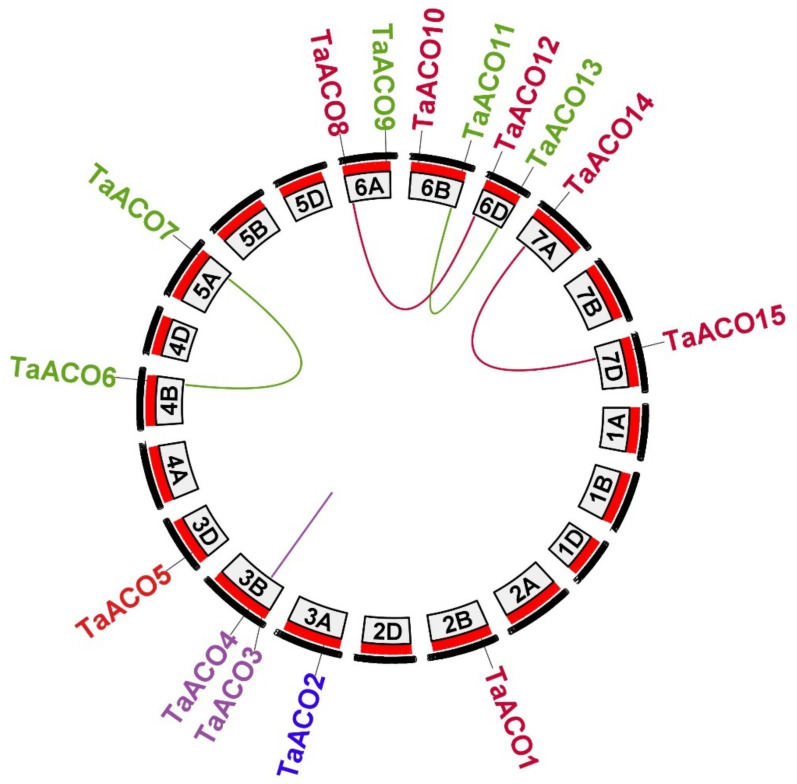
Chromosomal location and duplicated ACO genes in *T. aestivum*. Duplicated ACO genes are linked with distinct color lines and the figure was produced via TB tools.

Conversely, none of *TaACO* genes were mapped on the chromosome 1 (A, B & D), 2 (A &D), 4 (A & D), 5 (B & D), or 7 (B). Thus, these results indicated that the *TaACO* gene family members were unequally distributed on the wheat chromosomes. Further, we also scanned for the gene duplication in the ACO gene family in wheat. The phylogenetic tree of *TaACO*s has shown some gene duplications (Figure S3 and S4). We observed that ten *TaACO* genes were involved in duplication events (Figure 3 and Figure S4 and Table S4), which indicates that the increase of the ACO gene family in wheat was mainly caused via segmental and whole-genome duplication. 

**Figure 4:**
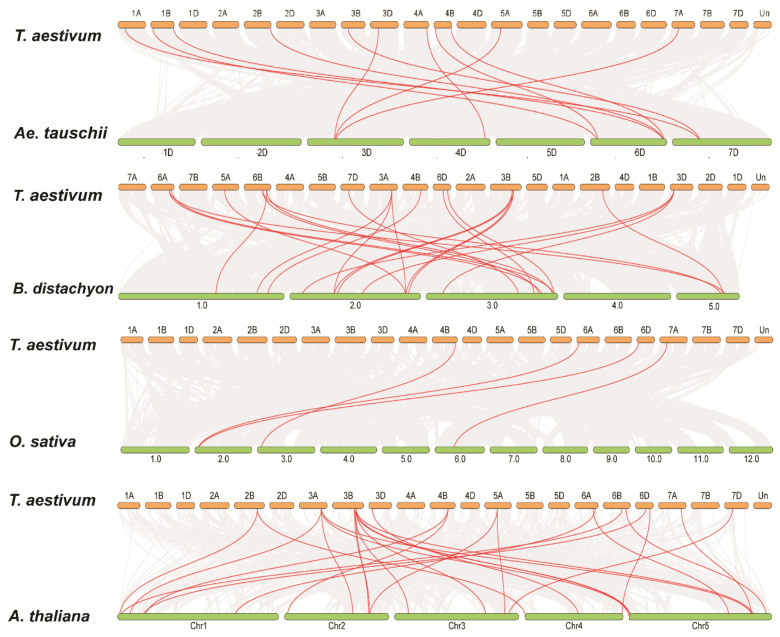
Syntenic connections of *TaACO* genes between *A. tauschii, B. distachyon, O. sativa*, and *A. thaliana*. The gray lines in the background denote the collinear blocks within *T. aestivum* and other plant species genomes, whereas the red lines indicate the syntenic ACO gene pairs.

To determine the selection force of duplicated *TaACO* genes, we calculated the Ka/Ks values for the five pairs of *TaACO* genes (Figure S4; Table S4). The Ka/Ks value was <1 for the five *TaACO* genes, pointing out that *TaACO* genes undergo a solid purifying selection with minor alterations, and subsequent duplication. Therefore, these results suggest the preserved evolution of *TaACO* genes. We also examined the synteny analysis of *TaACO* genes with model plants and other wheat relatives, for instance, *O. sativa A. thaliana*, *Ae. tauschii*, *B. distachyon*, and *T. dicoccoides*; the MCScanX was used to identify the orthologous pair genes between these plant genomes (Figure 4 and Table S5). 

We found 13, 15, 17, 10, and 29 orthologous genes between TaACOs with other ACOs in *O. sativa A. thaliana*, *Ae. tauschii*, *B. distachyon*, and *T. dicoccoides*, respectively. The results show that 4, 22, 11, and 20 *TaACO* genes were collinear with ACO genes in *O. sativa A. thaliana*, *Ae. tauschii*, and *B. distachyon*, respectively. Except for the *TaACO1* gene, all had two orthologous pairs of *TaACO* genes, which may play critical functions in the evolution of *TaACO*s. Thus, these outcomes indicate that *TaACO* genes may result from other crop ortholog genes.

### 2.3. Gene Structure and Conserved Motif and Protein Tertiary Structure Analysis of TaACO Genes

**Figure 5:**
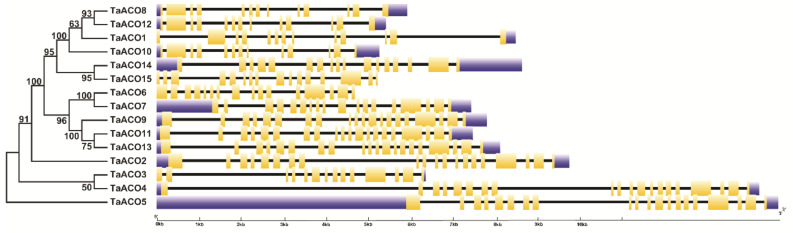
Gene structure organization of the *TaACO* genes. Blue boxes indicate the untranslated regions, yellow boxes denote the exons, and dark black lines signify the introns. The lengths of black lines and boxes are based on gene length.

We also examined the structural features of the *TaACO* genes (Figure 5). Gene structure analysis showed that *TaACO* genes significantly differed in gene structure (Figure 5 and Figure S5). 

**Figure 6:**
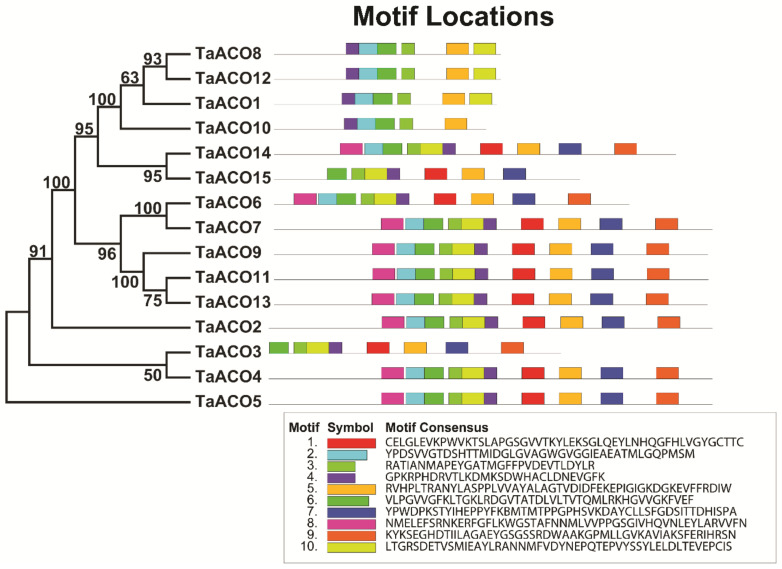
The motifs of TaACO genes are predicted by MEME. Distinct colored boxes denoting the various preserved motifs having differing sizes and sequences.

*TaACO* genes comprise 12–19 introns, for instance, *TaACO1*, *TaACO2*, *TaACO3*, and *TaACO4* had the minimum twelve introns, while a maximum of nineteen introns were identified in the *TaACO9*, *TaACO10*, *TaACO11*, *TaACO12*, *TaACO14*, and *TaACO15*. To understand the functions of *TaACO* genes, we analyzed the conserved motif of TaACO protein by the Multiple Em for Motif Elicitation (MEME) website. Finally, we identified the ten conserved motifs in TaACO proteins (Figure 6 and Figure S6). The TaACO gene family was discovered by the existence of aconitase domain (pfam PF 00330) and all fifteen TaACO proteins contain at least one aconitase motif (Table S6) which participates in isomerization of citrate to isocitrate in the TCA cycle. Additionally, the alignment of amino acid sequence also revealed that TaACO proteins shared a conserved aconitase domain (Figures S7 and S8A). Further, to understand the biological function of TaACO genes in wheat, the three-dimensional (3D) structure of TaACO proteins were predicted by phyre2 online web tools (Figure S8B). TaACO proteins consist of four globular domains with a linker fragment. 

### 2.4. Prediction of Cis-Acting Regulatory Elements (CAREs) in TaACO Genes 

**Figure 7:**
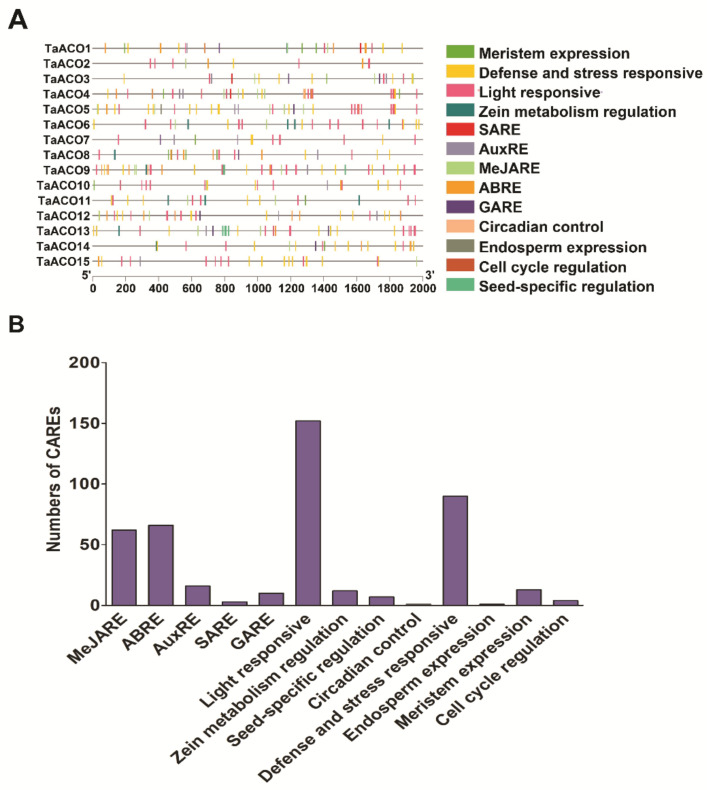
Cis-acting regulatory elements (CAREs) predicted in the *TaACO* promoters using the PlantCARE website. (**A**) The different colors represent the various CARE-related to phytohormone, defense and stress, growth, and development. (**B**) Most frequently predicted CAREs in the *TaACO* promoter regions.

To examine the precise functions of *TaACO* genes, 2000 base pair upstream sequences of *TaACO* genes were evaluated via PlantCARE online web server. This analysis has shown that *TaACO* promoters comprise different cis-elements related to plant hormones, developmental processes, defense and stress response (Figure 7A and Table S7). 

*TaACO* genes contain the five phytohormone responsive elements, such as the gibberellin responsive element (GARE), the auxin-responsive element (AuxRE), the abscisic acid response element (ABRE), the salicylic acid response element (SARE), and the MeJA response element (MeJARE). The CAREs fit into light responsive, stress responsive, ABRE, and MeJARE, which were the ubiquitous presence in the promoter region of *TaACO* gene family members (Figure 7B). Hence, these findings indicate that *TaACO*s play an essential role in plant development and response to diverse stresses. Further, *TaACO*s also comprise CARE related to meristem expression, zein metabolism, circadian control, endosperm expression, cell cycle regulation, and seed-specific regulation. The CAREs reported in the *TaACO* gene family demonstrated that *TaACO*s might be involved in various biological processes. Thus, these results provide crucial information to understand the intricate regulatory network of the *TaACO* gene family in response to plant hormones, multifactorial stress, and different developmental processes.

### 2.5. Gene Ontology Analysis of TaACO Genes 

Gene ontology (GO) enrichment analysis help in elucidating the function of any genes by comparing amino acid sequence with the known function of other plant species genes. All TaACO gens were magnificently interpreted and assigned GO terms by AgriGO, and further confirmed by EggNOG database (Figure S9; Tables S8 and S9) that reverted the similar outcomes as AgriGO. TaACO genes have shown enrichment in the tricarboxylic acid metabolic process (GO:0072350), citrate metabolic process (GO:0006101), isocitrate metabolic process GO:0006102, carbohydrate metabolic (GO:0005975), nucleoside phosphate metabolic process GO:0006753, glyoxylate metabolic process (GO:0046487), cellular respiration (GO:0045333), response to metal ion (GO:0010038), response to oxidative stress (GO:0006979), response to osmotic stress (GO:0006970) in the biological process category (Figure S9A), while in the cellular class, TaACO has displayed enrichment in the cytoplasm (GO:0005737), intracellular organelle (GO:0043229), membrane-bounded organelle (GO:0043227), intracellular membrane-bounded organelle (GO:0043231) (Figure S9B). In addition, the prediction of subcellular localization also returns similar results (Table 1). In the molecular function class, catalytic activity (GO:0003824) and binding (GO:0005488) were the predominant groups that participated in the TCA cycle (Figure S9C). Thus, these findings indicate that TaACO genes play an important function in plant developmental processes.

### 2.6. Expression Profiling of TaACO Genes in Different Tissues, Developmental Stages, and Stress Conditions 

**Figure 8:**
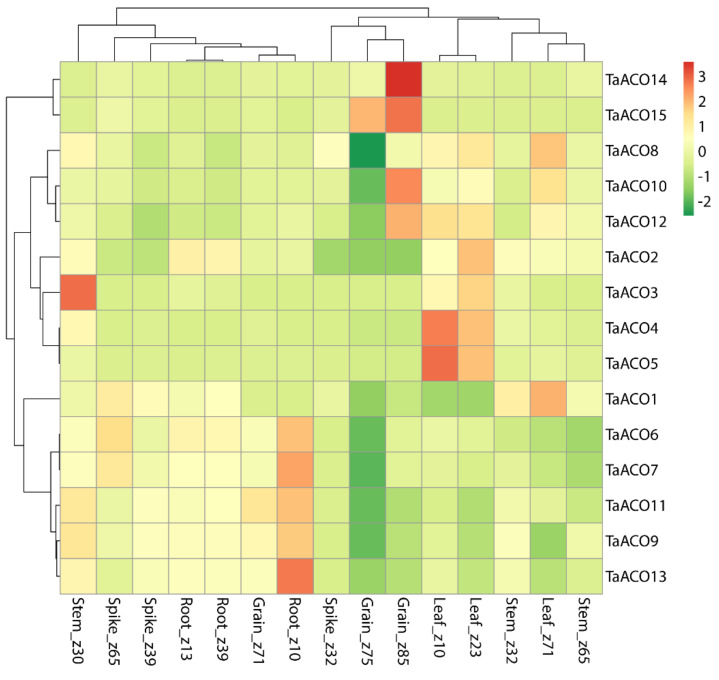
Heatmap demonstrating expression pattern of the *TaACO*s in different tissues and developmental stages. Columns indicate genes and rows denote different tissues and developmental stages. TPM numbers were directly used to produce the heatmaps.

To understand the role of *TaACO* genes, the expression profiling of *TaACO* genes were investigated in different tissues, developmental stages, and stress environments. The TPM values of *TaACO*s genes were downloaded from the Wheat Expression Browser. These TPM numbers were used to create heatmaps and carry out a principal component analysis (Figure 8, Figures S10A,B and S11). 

To understand the expression patterns of *TaACO*s, five tissues were taken from three different developmental stages. *TaACO*s genes exhibited differential expression in the various tissues and different developmental stages (Figure 8); for instance, the expression levels of *TaACO4*, *TaACO5*, and *TaACO12* in leaf_z10, the expression levels of *TaACO2*, *TaACO3*, *TaACO4*, *TaACO5*, *TaACO8*, and *TaACO12* in leaf_z23, and *TaACO1*, *TaACO8* and *TaACO10* were significantly elevated in leaf_z71.

The expression of *TaACO15* in grain_z75 was raised. The expression of *TaACO10*, *TaACO12*, *TaACO14*, and *TaACO15* were elevated in grain_z85. In addition, the expression of *TaACO6*, *TaACO7*, *TaACO9*, *TaACO11*, and *TaACO13* was significantly raised in root_z10, and *TaACO3* was upregulated in spike_z30. These findings demonstrated that the *TaACO* gene family might be implicated in various plant developmental processes.

**Figure 9:**
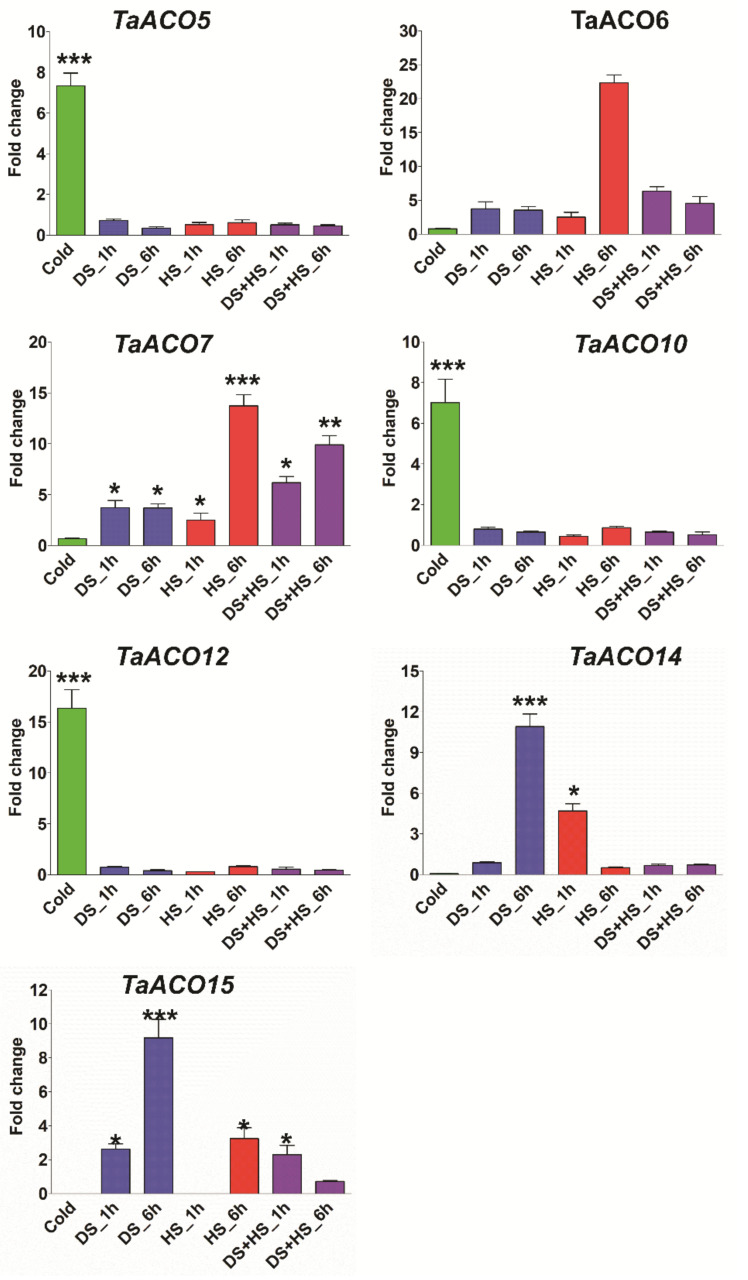
qRT-PCR analysis of randomly selected *TaACO* genes in different stress conditions to validate RNA seq data. The wheat actin gene was used as the internal control. The Asterisk denotes significant variances compared to the control, top of the bars signifies outcomes of the Tukey HSD test at the <0.05 (* *p* < 0.05) ** *p* lies in between the values of 0.05 and 0.001, and <0.001 level (*** *p* < 0.001). *** meaning expression is significantly induced compared with others. Error bars display standard deviation (SD). Data are mean ± SD (*n* = 3).

Expression profiling of *TaACO* was also examined in various stress conditions including cold, drought, heat, septoria tritici blotch, powdery mildew, and stripe rust (Figure S11). The expression level of *TaACO1* was significantly elevated in PM24h, while expression of *TaACO9*, *TaACO13*, and *TaACO15* was significantly induced in PM48h, and expression of *TaACO2*, *TaACO4*, and *TaACO8* was slightly upregulated in PM72h. *TaACO2*, *TaACO4*, *TaACO5*, and *TaACO8* have shown elevated levels in SR72h, whereas the expression of *TaACO11* was highly raised in Zt14d. Further, the differential expression pattern was also noticed for *TaACO* genes in abiotic stress. The expression level of *TaACO5*, *TaACO10*, and *TaACO12* was significantly elevated in cold. Similarly, the expression level of *TaACO6* and *TaACO7* were highly induced in heat stress after 6 h. *TaACO14* and *TaACO15* have displayed higher expression in drought stress after 6 h. *TaACO6* and *TaACO7* also showed elevated expression levels in combined drought and heat stress after 6 h (Figure S11). Furthermore, the expression kinetics of *TaACO* genes were confirmed by quantitative reverse transcription PCR (qRT-PCR). qRT-PCR analysis revealed that an almost similar expression pattern was observed under abiotic stress conditions (Figure 9). Collectively, these results have shown that *TaACO* genes may participate in different developmental processes and respond to biotic and abiotic stresses in wheat.

### 2.7. Protein–Protein Interaction Network Analysis of TaACO Genes 

**Figure 10:**
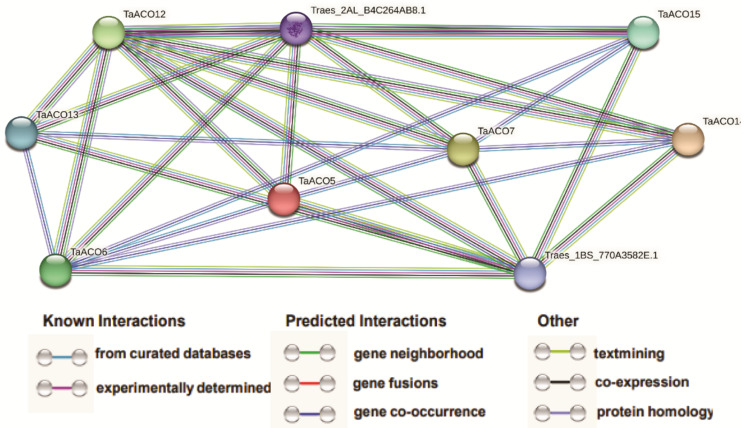
Protein–protein interaction (PPI) analysis of TaACOs proteins. PPI network generated via STRINGV9.1. Each knob denotes a protein, each edge represents an interaction.

To study the protein–protein interactions (PPIs) between TaACOs and other proteins, a protein network was generated by the STRING webserver (Figure 10 and Table S10).

According to the predicted results, we reported that TaACO interacted with 98 different wheat proteins. TaACO5 could interact with 13 other wheat proteins, for instance, Traes_1BS_770A3582E.1, Traes_2AL_B4C264AB8.1, Traes_2BL_6552196A1.1, TaACO12, Traes_2DL_AE61DD300.2, Traes_3B_0B9FADF42.1, TaACO7, TaACO6, Traes_3DS_0E5F1E5A4.2, Traes_5AL_1F730D0C4.3, Traes_5DL_E6D11E867.1, Traes_5BL_E3904C0B8.1, and Traes_5BL_1D75C197C.1. TaACO6 and TaACO7 could interact with 15 other wheat proteins, such as Traes_1BS_770A3582E.1, Traes_2AL_B4C264AB8.1, Traes_2BL_6552196A1.1, TaACO12, Traes_2DL_AE61DD300.2, Traes_3B_0B9FADF42.1, TaACO5, Traes_3DS_0E5F1E5A4.2, Traes_5AL_1F730D0C4.3, TaACO15, TaACO14, TaACO13, Traes_5BL_1D75C197C.1, Traes_5BL_E3904C0B8.1, and Traes_5DL_E6D11E867.1, while TaACO12 can interact with Traes_1BS_770A3582E.1, Traes_2AL_B4C264AB8.1, Traes_2BL_6552196A1.1, TaACO7, TaACO13, TaACO14, TaACO5, TaACO6, TaACO15, Traes_5DL_E6D11E867.1, Traes_5AL_1F730D0C4.3, Traes_5BL_1D75C197C.1, Traes_5BL_E3904C0B8.1, Traes_3B_0B9FADF42.1, Traes_2DL_AE61DD300.2, and Traes_3DS_0E5F1E5A4.2, which are 3-isopropylmalate (IPM) dehydrogenase and citrate synthase. IPM dehydrogenase enzyme catalyzes the oxidation and decarboxylation of IPM to ketoleucine in the presence of NAD+. Further, transamination of ketoleucine yields leucine in the leucine biosynthetic pathway [38,39]. Citrate synthase (CS) catalyzes the condensation of oxaloacetate (OAA) and acetyl coenzyme A to produce citrate in the TCA cycle [40,41]. These results provide valuable information to understand the biological functions of TaACO genes.

## 3. Discussion

The aconitase gene family in plants plays an important role in the glyoxylate and TCA cycles. ACO gene family has also been identified and characterized in other crops including three genes in *A. thaliana*, eight in *G. max*, two in *B. distachyon,* six in *Z. mays*, four in *S. bicolor*, and four in *C. clementina* [1,21]. However, for the first time, we identified the ACO gene family in wheat. Several researchers have shown that ACO genes are also present in primitive plants and expanded during evolution [1]. In this work, we performed an in silico analysis and identified the 15 ACO genes in wheat (Table 1). The phylogenetic analysis *A. thaliana*, *G. max*, *T. aestivum*, *B. distachyon, S. bicolor, Z. mays*, *P. patens*, and *S. moellendorffii* revealed that ACO genes were divided into six classes (Figure 1). The phylogenetic tree has shown that groups I and II were specific to monocot, whereas group IV had dicot-specific ACOs, and group V contained primitive-landed-specific ACOs (Figure 1 and Figure S2). The origin of this kind of genes suggests specific functions in the monocots that play a critical role in morphological development [7,26,28,42]. However, ACO genes were dispersed into the well-known clade *Arabidopsis*, soybean, sorghum, maize, and wheat, suggesting that the ACOs gene may originate from a common ancestor. The TaACO gene family is expanded in the wheat and contained more ACO genes compared to the earlier described ACOs in *A. thaliana*, *G. max*, *Z. mays*, *O. sativa*, *B. distachyon, S. bicolor, P. trichocarpa*, and *C. clementina* [1,21]. Tandem, segmental, and whole-genome duplications are the major forces involved in the gene duplication processes and expanding the gene family in various plant species [43,44]. Further, we also scanned for the gene duplication analysis of ACO gene family members in wheat. The phylogenetic tree of TaACOs has shown some gene duplications (Figure S4). We found ten *TaACO* genes involved in the duplication process (Figure 3 and Table S4). The duplicated gene pairs are *TaACO8:TaACO12*, *TaACO14:TaACO15*, *TaACO6:TaACO7*, and *TaACO11:TaACO13*, that indicate that the expansion of the ACO gene family in wheat was mainly caused via segmental duplication and whole genome duplication. Similarly, duplication event was reported in angiosperms, which play an important role in the expansion of the ACO genes. Segmental duplication was observed in *Z. mays*, *G. max*, and *P. trichocarpa*. Segmental gene duplication might be the main driving force of ACO gene family expansion in the *Z. mays*, *G. max*, and *P. trichocarpa* [1]. Similar results were observed in citrus [21]. Additionally, the Ka/Ks value was <1 for the five *TaACO* genes pointing out that *TaACO* genes undergo a strong purifying selection with minor alterations after duplication. Therefore, these results suggest the conserved evolution of *TaACO* genes. Furthermore, chromosomal mapping of *TaACOs* showed that a single gene was found on the chromosomes 2B, 3A, 3D, 4B, 5A, 7A, and 7D, while two *TaACO* genes were mapped on the chromosomes 3B, 6A, 6B, and 6D (Figure S3A). Conversely, none of the *TaACO* genes were mapped on the chromosomes 1A, 1B, 1D, 2A, 2D, 4A, 4D, 5B, 5D, and 7B. Hence, these results showed that the 15 TaACOs were unequally dispersed on the wheat chromosomes (Figure 2 and Figure S3B). Likewise, results were reported in PIN-FORMED (PIN), brassinazole-resistant (BZR), and PERKs gene family in wheat [26,27,28]. Thus, the unequal distribution of *TaACO* genes on the 11 chromosomes of wheat suggests that genes disappear and add through segmental duplication or whole genome events, and there might be errors during genome sequencing and assembly. Moreover, we found 13, 15, 17, 10, and 29 orthologous genes between *TaACOs* with other ACOs in *O. sativa A. thaliana*, *Ae. tauschii*, *B. distachyon*, and *T. dicoccoides*, respectively. The results show that 4, 22, 11, and 20 *TaACO* genes were collinear with ACO genes in *O. sativa A. thaliana*, *Ae. tauschii*, and *B. distachyon*, respectively. Except for the*TaACO1* gene, all had two orthologous pairs of *TaACO* genes, which may play key functions in the evolution of *TaACOs*. During the evolution, there have been changes in the upstream and/or exon regions of duplicated genes, which has caused a change in their expression pattern and function [45,46]. Therefore, these findings indicate that *TaACO* genes may result from other plant orthologous genes.

Gene structure analysis provided key clues to understanding gene function and evolution. Gene structure analysis revealed that *TaACO* genes greatly differed in gene structure. *TaACO* genes comprise 12-19 introns, for instance, *TaACO1*, *TaACO2*, *TaACO3*, *TaACO4* had the minimum of twelve introns, while a maximum of nineteen introns were identified in the *TaACO9*, *TaACO10*, *TaACO11*, *TaACO12*, *TaACO14*, and *TaACO15* (Figure 5 and Figure S5). The size of the intron is one of the important factors that influence the gene size, for example, a notable variance in gene size was observed between the largest gene *TaACO4* (9 kb) and the smallest gene *TaACO6* (2 kb) owing to the total intron length (9 kb vs. 2 kb). Several researchers have demonstrated the importance of introns in the evolution of various genes in plants [47,48]. Many gene families contain lower, absent, and higher numbers of introns [49,50]. The number of exon and intron differences may be suitable for the documentation of evolutionary mechanisms [50]. In this work, all 15 *TaACO* genes had higher numbers and bigger introns during evolution in wheat. In this study, genes have shown the variable intron-exon structures presenting diverse functions. The different gene structures of *TaACO* could result in diverse functions due to selection pressure during wheat genome evolution. Furthermore, examination of motifs revealed that all TaACO proteins comprise the 10 diverse motifs (Figure 6 and Figure S6). We noticed that seven motifs were found in all the TaACO proteins (Motif 2, 3, 4, 5, 6, 9, and 10). Hence, we speculated that the same group of genes in the phylogenetic tree might share similar functions, even in the different plant species. Moreover, multiple protein sequence alignment of TaACO with other ACO proteins exhibited that TaACO shared a conserved aconitase domain (Figures S7 and S8A). WOX and YABBY gene family was evolutionarily conserved in *G. hirsutum* [51]. Moreover, 3D structure analysis has shown that TaACO proteins comprise the four globular domains with a linker fragment (Figure S8B). Consequently, this result will help in understanding and explaining the molecular basis of the isomerization of citrate to isocitrate in the TCA cycle. 

CAREs present in the promoter section play a key role in the function and regulation of genes [52]. *TaACO* promoter regions comprise the different CARE elements related to phytohormones, related to growth and stress response (Figure 7A and Table S7). Additionally, ten CAREs were detected in each of the *TaACO* promoter regions (Table S7). A total of eight CAREs were predicted which are related to light response such as G-box (CARE participates in light response), Sp1 (light responsive element), GT1-motif (light responsive element), MRE (MYB binding site involved in light responsiveness), Box 4 (part of a conserved DNA module involved in light responsiveness), Box II (part of a light responsive element), chs-Unit 1 m1 (part of a light responsive element), and chs-CMA1a (part of a light responsive element) [53,54]. Further, we also found four CAREs related to growth, for instance, O_2_-site (zein metabolism regulation), GCN4-motif (endosperm expression), and CAT-box (meristem expression) [55,56]. Additionally, we detected phytohormone CARE related, for example, MeJA-responsive element (CGTCA-motif), abscisic acid-responsive element (ABRE), salicylic acid responsiveness (TCA-element), and gibberellin-responsive element (TATC-box-motif). The light responsive, defense and stress responsive, ABRE, and MeJARE elements were prevalent in *TaACOs* promoters (Figure 7B and Table S7). Furthermore, we also found that CAREs participated in the response to multifactorial stress, including low-temperature responsiveness (LTR) and defense and stress responsiveness (ARE and GC-motif). These results suggest that *TaACO* genes might be regulated by diverse developmental processes, phytohormone and different biotic and abiotic stress conditions. Many studies have demonstrated that light plays an essential role in plant development processes [57]. Light also modulates the expression level of aconitase genes [58]. The microarray analysis in *Arabidopsis* has shown light suppression of the mitochondrial aconitase [59]. This could lead to the decreased respiration of mitochondria and enhanced photosynthesis efficiency. Hence, these results suggest that *TaACOs* play an essential role in plant development and response to diverse stress. Further, *TaACOs* also comprise CARE related to meristem expression, zein metabolism, circadian control, endosperm expression, seed-specific, and cell cycle regulation. The CAREs reported in the *TaACO* gene family demonstrated that *TaACOs* may be involved in a variety of biological processes. Thus, these results provide crucial information to understand the intricate regulatory network of the *TaACO* gene family in response to plant hormones, multifactorial stress, and different developmental processes.

*TaACOs* genes exhibited differential expression in the various tissues (Figure 8), for instance, the expression level of *TaACO4, TaACO5*, and *TaACO12* in leaf_z10, *TaACO2, TaACO3, TaACO4, TaACO5, TaACO8*, and *TaACO12* in leaf_z23, and *TaACO1, TaACO8* and *TaACO10* were significantly elevated in leaf_z71. The temporal and spatial expression pattern of *TaACO* genes indicates that *TaACO* genes might have a function in different tissues and various developmental stages in wheat. In addition, our gene ontology results revealed a wide range of roles of *TaACO* genes in the biological processes (Figure S9A–C). These results demonstrated that *TaACO* genes play an important role in different tissues and developmental stages. Similarly, the expression level of PtACO3 was elevated in the root tissues of poplar [1]. The expression of cytosolic and mitochondrial forms of aconitase were regulated by phytochrome in maize leaves [24]. The expression of *AtACO2* and *AtACO3* were highly raised in the cauline leaf tissues in *Arabidopsis* [1]. Likewise, two *SmACO* genes were differentially expressed in leaf, stem, and root tissues. *ZmACO4* showed a higher expression compared to the other five ACO genes in maize [1]. Aconitase genes have been detected in mitochondria and cytosol. The functions of each isoform are diverse [60,61,62]. Eprintsev et al. [23] also reported the differential activity and expression of *Aco1* and *Aco4* in scutellum during the germination of maize seeds. Further, Aconitase knockdown and knockdown mutant of Drosophila showed a lethal effect in Drosophila, suggesting that Aconitase is important for viability [63]. We assumed that all key enzymes of the TCA cycle might be controlled by light via aconitase [64]. However, no direct experimental evidence of light governing the aconitase function has been found. Carrari and colleague [16] reported that the aconitase activity decreased in the ACO mutant of tomato; however, the photosynthesis rate was significantly increased in tomato. CcAco3 was constitutively expressed—although at low levels—along all phases of fruit pulp ripening in citrus [21]. Thus, these findings demonstrated that the *TaACO* gene family may be implicated in various plant developmental processes.

Many age-linked and mitochondrial disorders are related to raised expression levels of reactive oxygen species following decreased activity of ACO [65]. The differential expression pattern was also noticed for *TaACO* genes in abiotic stress (Figure S11). The expression level of *TaACO5, TaACO10*, and *TaACO12* was significantly elevated in cold. Similarly, the expression level of *TaACO6* and *TaACO7* were highly induced in heat stress after 6 h. *TaACO14* and *TaACO15* displayed higher expression in drought stress after 6 h. *TaACO6* and *TaACO7* also showed elevated expression levels in combined drought and heat stress after 6 h (Figure S11). The elevated expression level of *TaACO* genes under cold, heat, and drought stress indicates that *TaACO* genes might be involved in stress tolerance in wheat by coordinating regulation among the TaACOs. Several studies demonstrated that ACO genes have been implicated in response to oxidative stress in plants [11,12,66]. ABA and MeJA play a crucial role in oxidative homeostasis in plants. The CARE analyses in this work also revealed that all *TaACO* genes contained CAREs response to different phytohormones, defense and stress responsiveness. In addition, Wang et al. [1] showed that numerous ACO genes, for instance, *BdACO2*, *GmACO3*, and *OsACO1* had an elevated expression level in root tissues of *B. distachyon*, *G. max*, and *O. sativa* during stress conditions. The elevated expression of citrate triggers the expression of the oxidase, thereby inhibiting ROS formation in mitochondria [66]. Furthermore, the qRT-PCR analysis revealed that an almost similar expression pattern was observed under abiotic stress conditions (Figure 9). Moreover, our PPI results also revealed that TaACO interacted with 98 different wheat proteins. TaACO5 could interact with 13 other wheat proteins, while TaACO6 and TaACO7 could interact with 15 other wheat proteins. Hence, the TaACO protein also interacts with other proteins and modulates the metabolism and signaling pathways in wheat. These results suggested that plant ACO genes might be participated in oxidative stress by responding to different metabolisms and phytohormones. Therefore, these results have shown that *TaACO* genes may participate in different developmental processes and respond to abiotic stresses in wheat. Collectively, these findings demonstrated that *TaACOs* might regulate a wide range of cellular processes, phytohormones signaling and response to various stress; of course, this is important and must be proved by experimental data in the near future. Therefore, these results provided foundational information to elucidate the precise function of *TaACO* genes in plant developmental processes, responses to different plant hormones, and diverse stresses. 

## 4. Materials and Methods

### 4.1. Identification of ACO Genes in Wheat 

To identify the ACO gene in wheat, ACO protein sequences of *A.thaliana, O.sativa*, *G.max*, *B. distachyon, S. bicolor, P. patens*, and *S. moellendorffii* were downloaded from the Phytozome (https://phytozome-next.jgi.doe.gov/ accessed on 15 October 2022). The twenty-eight ACO protein sequences used as query against the *T. aestivum* proteome with an e-value of 10^−5^ and bit-score>100 kept cut-off to find *TaACO* genes and, finally, the BLASTp result was tabulated. Further verified for the existence of aconitase domain using other databases: InterPro [67], HMMscan [68], National Center for Biotechnology Information CDD [69] and Simple Modular 132 Architecture Research Tool (SMART, http://smart.emblheidelberg.de/ accessed on 15 October 2022). Eventually, the protein sequences with aconitase domain were selected and named according to their positions on the chromosomes. 

### 4.2. Biophysical Characteristics, Subcellular Localization Genomic Localization, Gene Duplication and Synteny Analysis

The biophysical features of TaACO proteins, including isoelectric point, lengths, and molecular weight, were evaluated by the ExPASy server [70] and isoelectric point calculator [71]. Subcellular localization was predicted using CELLO (http://cello.life.nctu.edu.tw/ accessed on 18 October 2022), WoLEPSORT (http://www.genscript.com/wolf-psort.html accessed on 18 October 2022), and BUSCA online tools (http://busca.biocomp.unibo.it/ accessed on 18 October 2022). For the distribution on wheat chromosomes, the genomic positions of *TaACO* genes were obtained from Ensembl plants (http://plants.ensembl.org/biomart/martview accessed on 18 October 2022). PhenoGram (http://visualization.ritchielab.org/phenograms/plot accessed on 18 October 2022) was used to represent the ACO genes on the wheat chromosomes. McScan tools were used to find out gene duplication and synteny analysis [72]. The Ka/Ks value was calculated using the TBtools [73]. 

### 4.3. Gene Structure, Protein Motif, and 3D Structure 

The coding sequences, corresponding genomic sequences, and protein sequences of TaACO genes were retrieved from Ensembl plants (https://plants.ensembl.org/index.html accessed on 15 October 2022). Further, exon/intron structures were constructed using the Gene Structure Display Server 2.0 (http://gsds.gao-lab.org/ accessed on 17 October 2022). The protein motifs in the TaACO were elucidated using Multiple Em for Motif Elicitation ver.5.3.3. (http://meme-suite.org/tools/meme accessed on 18 October 2022) with default settings. The three-dimensional structure (3D) of TaACOs were produced using the Phyre2 web server (http://www.sbg.bio.ic.ac.uk/phyre2/html/page.cgi?id=index accessed on 18 October 2022). 

### 4.4. Gene Ontology, Promoter Cis-Acting Regulatory Elements (CAREs), and Protein Interaction Network Analysis 

TaACO protein sequences were evaluated for GO terms enrichment using the agriGO (http://bioinfo.cau.edu.cn/agriGO/ accessed on 20 October 2022) and EggNOG (http://eggnogdb.embl.de/#/app/emapper accessed on 20 October 2022). To identify CAREs, 2000 bp upstream sequences of ACO genes were downloaded from Ensemble Plants and analyzed using the PlantCARE online web server (http://bioinformatics.psb.ugent.be/webtools/plantcare/html/ accessed on 20 October 2022). Further, the most frequently occurring CAREs were presented using the TBtools [73]. The TaACO protein interaction network was examined using the STRING online webserver (https://string-db.org/cgi accessed on 20 October 2022).

### 4.5. Expression Analysis of TaACO Genes 

Gene expression data of TaACO genes in different wheat tissues (leaf, stem, root, spike, and grain) and different stress conditions (cold, drought, heat, combined drought, and heat) were obtained from WheatExp database (http://www.wheat-expression.com/ accessed on 22 October 2022). The expression pattern was presented as a heatmap based on transcript per million (TPM), which was mapped by ClustVis [74] and principal component analysis (PCA) plots generated by TBtools [73].

### 4.6. Plant Growth, Stress Conditions, and Quantitative Reverse Transcription PCR Analysis

Wheat seeds were seeded in plastic pots and grown in a greenhouse, and then 10-day-old seedlings were acclimatized for 2 days in growth chamber environments. The wheat seedlings were exposed to drought and heat stress (37 °C) for 1 h and 6 h, while cold stress was for 3 days (4 °C). For the combined drought and heat stress, first, the wheat seedling was exposed to drought stress and then subjected to heat stress for 1 h and 6 h at 37 °C in an incubator. Controls were kept at 25 °C. The drought-, heat-, and cold-stressed wheat seedlings were collected for RNA isolation and stored at −80 °C. Total RNA was extracted from the control, drought-, heat-, and cold-stressed wheat seedlings as described by [75,76]. cDNA was synthesized using the iScriptTM cDNA synthesis kit (Bio-Rad, Hercules, CA, USA). Wheat actin (AB181991) was used as the control to standardize the gene expression data and the qRT-PCR was executed with Applied Biosystems 7500 Fast Real-Time PCR. Each qRT-PCR reaction was performed with three technical replicates and repeated three times. The fold change value was calculated based on mean 2-ΔΔCT and finally fold change value was used to plot the graph [77,78]. All primers used in this study were tabulated in Table S11.

## 5. Conclusions

Wheat is major cereal crop and widely consumed staple food worldwide. However, global warming is becoming a severe threat to food security due to the constant climate changes, largely affecting plant growth and development, eventually the yield. This has raised a significant challenge for plant biologists to increase yield and improve wheat’s quality and various stress tolerance. In this work, we identified 15 ACO genes in wheat genome. Phylogenetic tree revealed that *TaACO* genes classified into six groups. Further, gene structure analysis of TaACOs has shown a distinctive evolutionary path. Furthermore, Ka/Ks ratio revealed that most *TaACO* genes experienced strong purifying selection during evolution. Numerous cis-acting regulatory elements were detected in the TaACO promoters, which might play a crucial role in plant development processes, phytohormone signaling, and are related to defense and stress. Moreover, expression profile revealed that *TaACO* genes were differentially expressed in different tissues, developmental stages, and abiotic stress conditions. Therefore, our results provide valuable information about the ACO gene family in wheat and contribute to the further characterization of ACOs during plant development and response to diverse biotic and abiotic stress.

## Figures and Tables

**Table 1:** Nomenclature and characteristics of the aconitase (ACOs) genes in wheat was detected using different computational tools.

Proposed Gene Name	Gene ID	Genomic Location	Orientation	CDS Length (bp)	Protein Length (aa)	Protein Name	Molecular Weight (KDa)	Isoelectric Point (pI)	GRAVY	Predicted Subcellular Localization
*TaACO1*	TraesCS2B02G181900	2B:156914707-156922970	Reverse	1506	501	Hypothetical protein CFC21_022353	54.79	6.09	−0.202	mitochondrion
*TaACO2*	TraesCS3A02G184000	3A:214122773-214123221	Reverse	3024	1007	Putative aconitate hydratase, cytoplasmic isoform	109.32	6.50	−0.132	chloroplast thylakoid membrane
*TaACO3*	TraesCS3B02G213800	3B:253633139-253633475	Reverse	1971	656	Putative aconitate hydratase, cytoplasmic	71.29	5.92	−0.155	cytoplasm
*TaACO4*	TraesCS3B02G213900	3B:253666186-253666845	Reverse	3015	1004	Putative aconitate hydratase, cytoplasmic isoform	108.95	6.59	−0.153	chloroplast thylakoid membrane
*TaACO5*	TraesCS3D02G188200	3D:174907841-174908490	Reverse	3015	1004	Putative aconitate hydratase, cytoplasmic isoform	108.89	6.38	−0.123	mitochondrion
*TaACO6*	TraesCS4B02G335100	4B:626011015-626011628	Reverse	2400	799	Putative aconitate hydratase, cytoplasmic	86.7	5.98	−0.073	extracellular space
*TaACO7*	TraesCS5A02G505000	5A:670410810-670411419	Reverse	2988	995	Putative aconitate hydratase, cytoplasmic	106.93	6.60	−0.128	chloroplast thylakoid membrane
*TaACO8*	TraesCS6A02G080600	6A:49189960-49195152	Forward	1533	510	3-isopropylmalate dehydratase large subunit, chloroplastic-like	55.16	6.43	−0.218	chloroplast thylakoid membrane
*TaACO9*	TraesCS6A02G400000	6A:609449354-609450370	Forward	2931	976	Putative aconitate hydratase, cytoplasmic	106.2	6.41	−0.184	chloroplast thylakoid lumen
*TaACO10*	TraesCS6B02G108100	6B:88711659-88715726	Forward	1434	477	Hypothetical protein CFC21_101948	51.92	5.97	−0.221	chloroplast thylakoid membrane
*TaACO11*	TraesCS6B02G440200	6B:705380681-705381704	Forward	2934	977	Putative aconitate hydratase, cytoplasmic	106.31	6.37	−0.209	chloroplast
*TaACO12*	TraesCS6D02G074300	6D:37921196-37926065	Forward	1533	510	3-isopropylmalate dehydratase large subunit, chloroplastic-like	55.13	6.43	−0.227	chloroplast thylakoid membrane
*TaACO13*	TraesCS6D02G384200	6D:462627391-462628393	Forward	2928	975	Putative aconitate hydratase, cytoplasmic	106.15	6.35	−0.174	chloroplast thylakoid lumen
*TaACO14*	TraesCS7A02G219100	7A:186527123-186528324	Reverse	2715	904	Aconitate hydratase, cytoplasmic-like	99.8	5.67	−0.145	cytoplasm
*TaACO15*	TraesCS7D02G223000	7D:184190377-184191564	Forward	2067	688	Aconitate hydratase, cytoplasmic isoform	75.71	6.19	−0.156	cytoplasm

MW: molecular weight; KDa: Kilo Dalton; aa: amino acids; bp: base pair; ID: identity; pI: isoelectric point.

## Data Availability

Data are available in the manuscript and in the Supplementary Materials.

## Supplementary Materials

The following are available online at https://www.mdpi.com/article/10.3390/plants11243475/s1. Figure S1: The molecular mass (kDa) and isoelectric point of plots of *TaACO* genes. Figure S2: Dispersal of TaACOs in a different group of the phylogenetic tree. Figure S3: Chromosomal locations of identified ACO genes and on the three sub-genomes of (A) Dispersal of ACO genes in the A, B and D sub-genomes. (B) Dispersal of ACO genes on wheat chromosomes. Figure S4: Evolutionary analysis of *TaACO* genes. A phylogenetic tree was produced using MEGA7 with the NJ method and 1000 bootstrap replications. A black asterisk denotes the duplicated gene pairs. Figure S5: The different number of exons and introns found in the TaACOs gene family. Figure S6: Sequence logo of TaACO motif and height of each mountain indicate the conservation at this position, whereas the height of the different letters represents the frequency of the corresponding amino acids. Figure S7: Alignment of the TaACO proteins and preserved aconitase domain is indicated with red color line. Amino acid residues conserved in all proteins were shaded blue and similar amino acids were gray shaded. Dashes denote gaps that led to exploiting the alignment of the homologous region. Figure S8: Alignment and three-dimensional structure of the TaACO protein sequences. (A) The aconitase domain is underlined with red color. Colored and shaded amino acids are chemically similar residues. Dashes indicate gaps introduced to maximize the alignment of the homologous region. (B) Predicted 3D structures TaACO proteins. Figure S9: The predicted GO term of TaACO gene family using AgriGO (A) Biological Process. (B) Cellular component. (C) Molecular function. Figure S10: PCA plots displaying grouping of different (A) Different tissues and developmental stages. (B) Diverse stress conditions based on the *TaACO* expression profiles. HS: Heat stress; DS: Drought stress; DS+HS: Combined drought and heat stress; PM: Powdery mildew; SR: Stripe rust; Zt: *Zymoseptoria tritici*; d: days; h: hour. Figure S11: Heatmaps signifying the expression profile of TaACO genes in biotic and abiotic stress conditions. TPM values were directly used to produce the heatmaps. HS: Heat stress; DS: Drought stress; DS+HS: Combined drought and heat stress; PM: Powdery mildew; SR: Stripe rust; Zt: *Zymoseptoria tritici*; d: days; h: hour. Table S1: TaACO genomic, conserved domain sequence, protein and promoter sequence. Table S2: The number of ACO genes found in the other crop species. Table S3: ACO proteins from *Arabidopsis*, rice, soybean, wheat, brachypodium, sorghum, selaginella, and physcomitrella used to produce phylogenetic tree. Table S4: Estimated nonsynonymous substitutions (Ka) and synonymous substitutions Ks ratio of duplicate gene pairs in TaACO gene family. Table S5: Orthologous relationships of TaACO genes with other ACO genes in *B. distachyon*, *Ae. tauschii*, *T. dicoccoides, O. sativa*, and *A. thaliana*. Table S6: Identified the aconitase domains in the TaACO proteins predicted using pfam with default parameters. Table S7: Identified the cis-regulatory elements in the *TaACO* promoter region. Table S8: Predicted significant Go term in the TaACO gene family by AgriGo analysis. Table S9: TaACO gene annotation using EggNOG database. Table S10: The PPI network between TaACO and other proteins in wheat. Table S11: qRT-PCR primers used for the *TaACO* genes in this study.
