# Supplementary material for: Genome-Wide Identification and Expression Profiling of Aconitase Gene Family Members Reveals Their Roles in Plant Development and Adaptation to Diverse Stress in Triticum aestivum L
Source: Plants (Basel). 2022 Dec 12;11(24):3475. doi: 10.3390/plants11243475 (PMC9782157; doi:10.3390/plants11243475)
Supplement: Supplementary file 1 [file plants-11-03475-s001.zip › TaACO supplmentary figures.pdf]

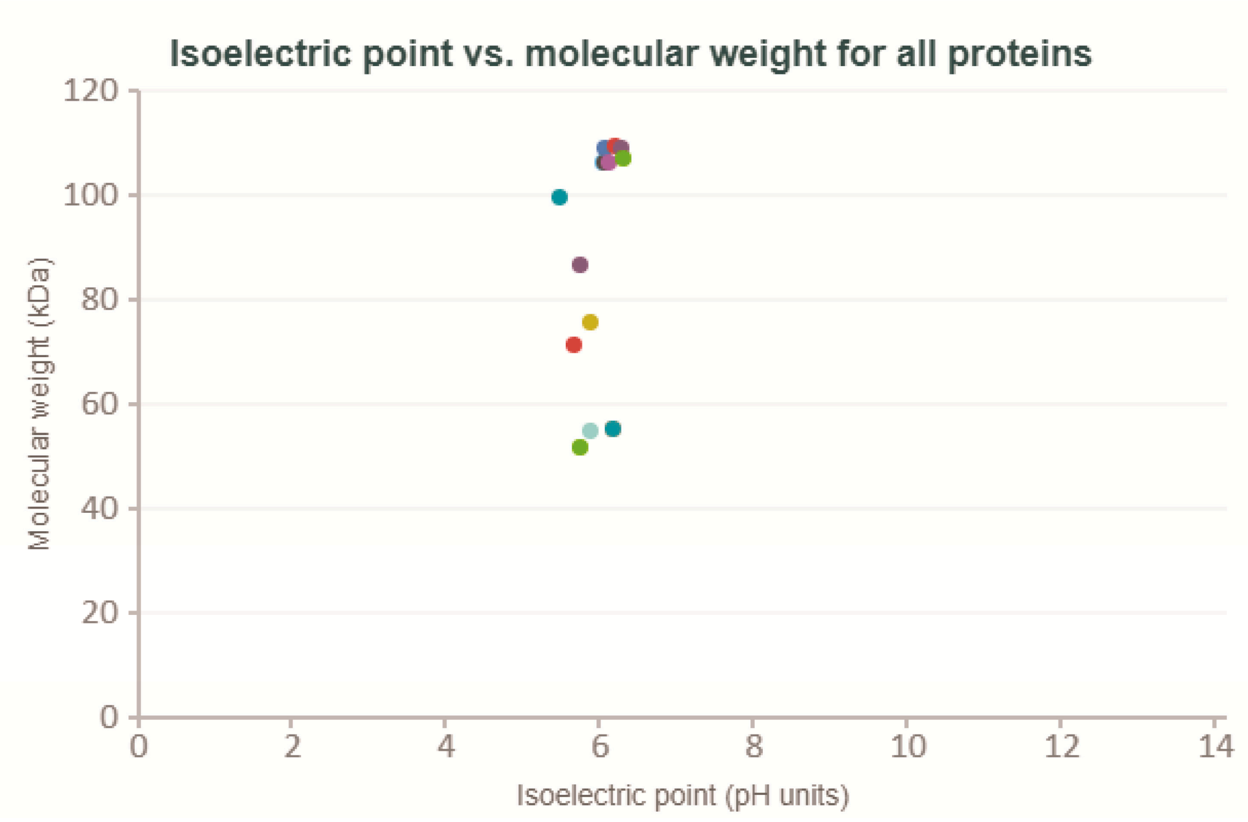

Figure S1. The molecular mass (kDa) and isoelectric point of plots of TaACO genes.

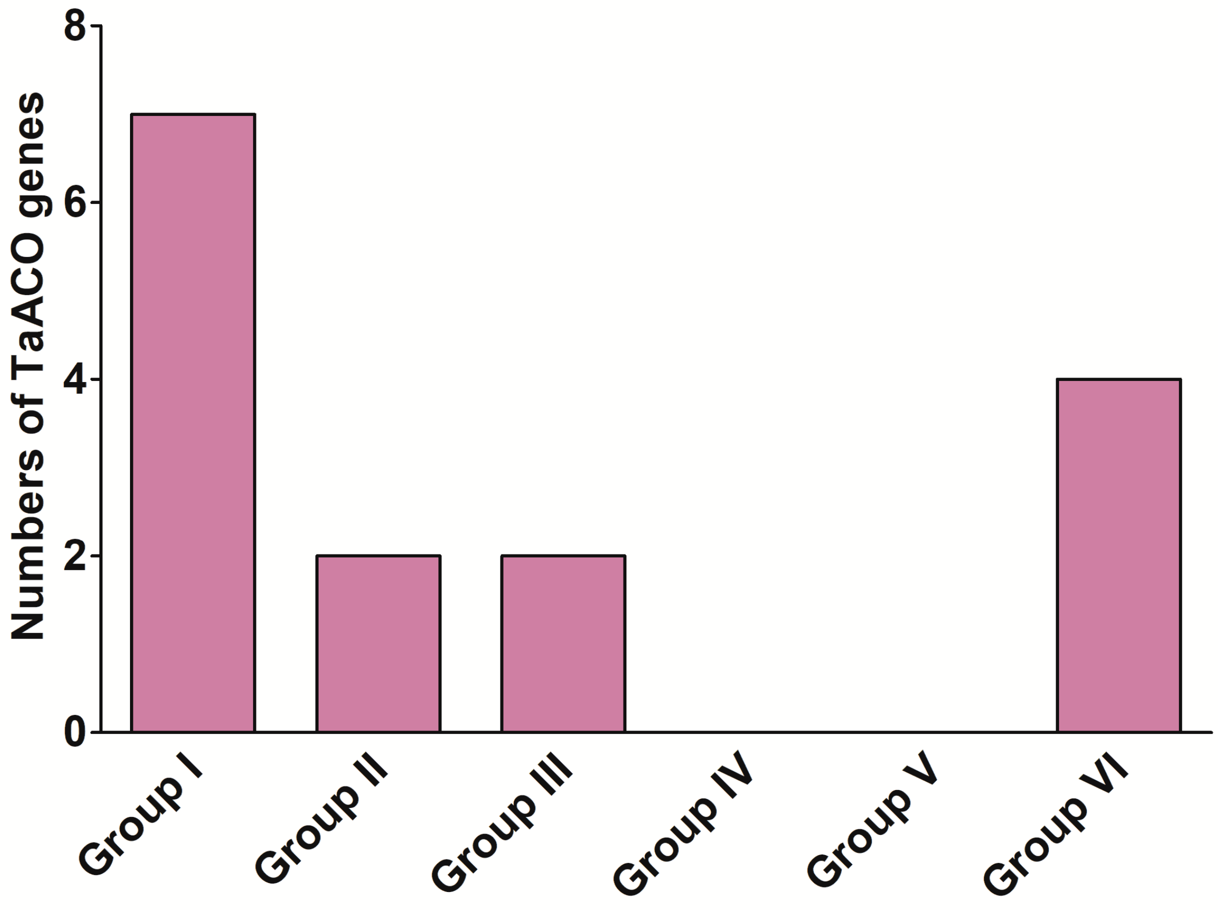

Figure S2. Dispersal of TaACOs in a different group of the phylogenetic tree.

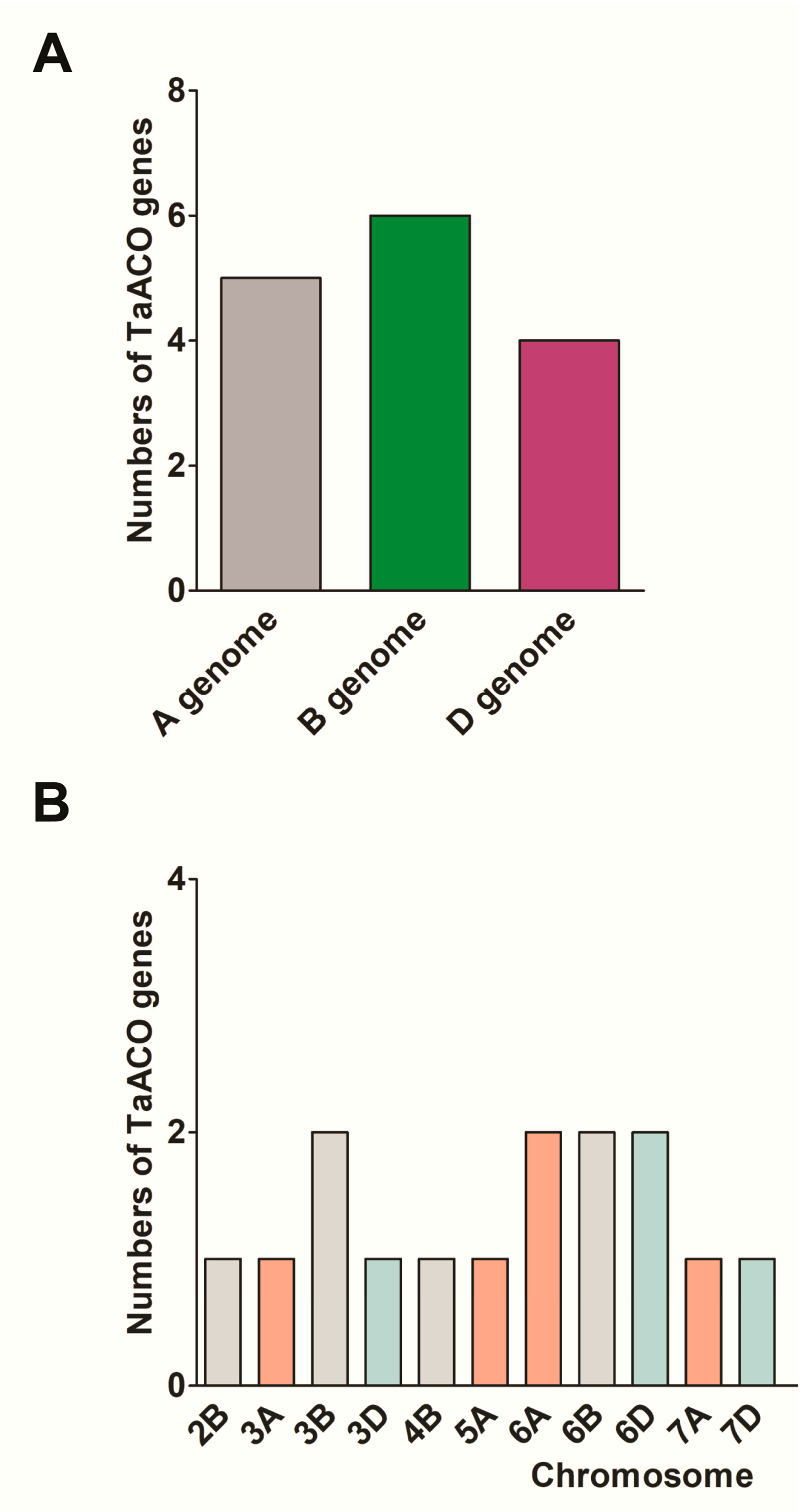

Figure S3. Chromosomal locations of identified ACO genes and on the three sub-genomes of (**A**) Dispersal of ACO genes in the A, B and D sub-genomes. (**B**) Dispersal of ACO genes on wheat chromosomes.

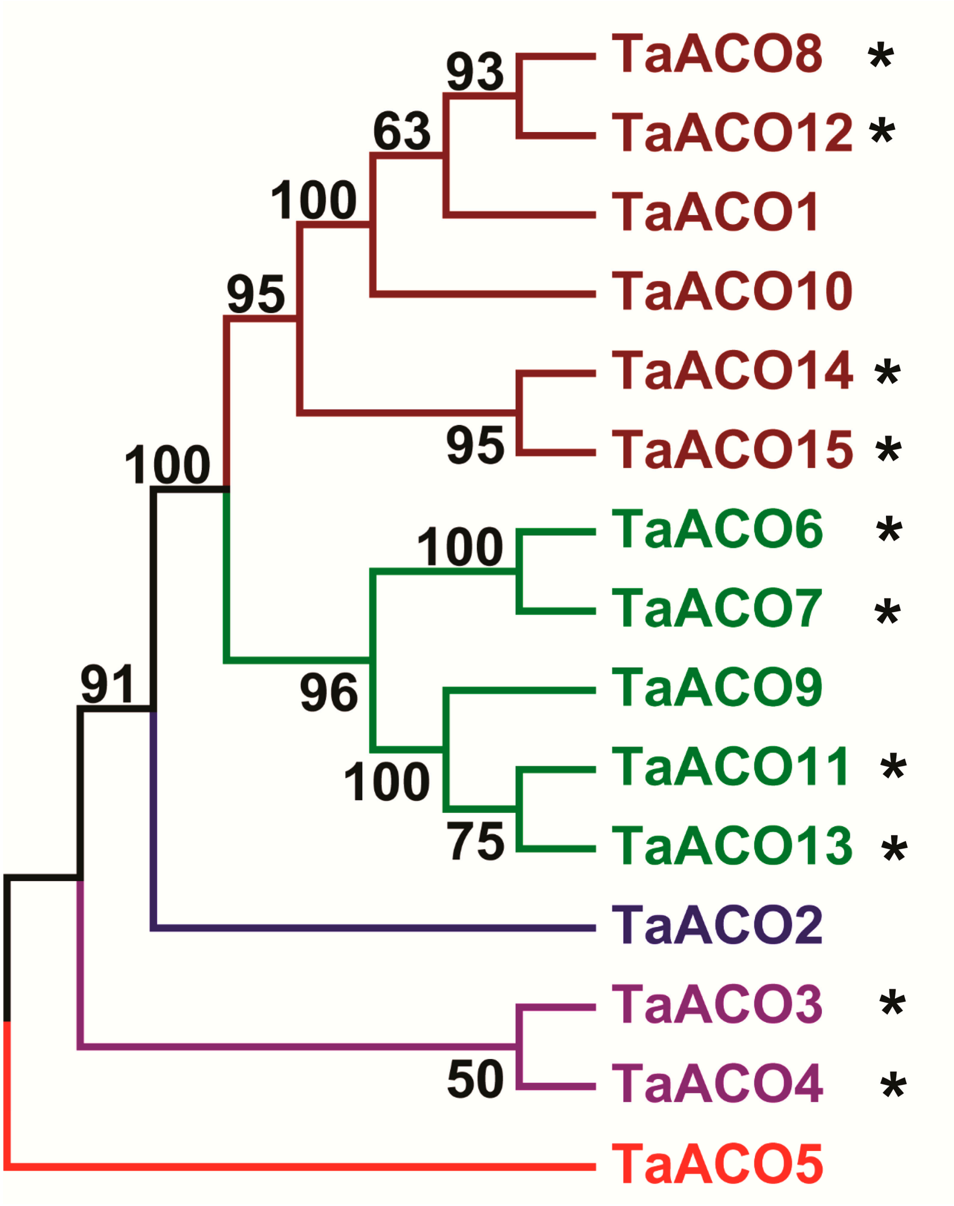

Figure S4. Evolutionary analysis of TaACO genes. A phylogenetic tree was produced using MEGA7 with the NJ method and 1000 bootstrap replications. A black asterisk denotes the duplicated gene pairs.

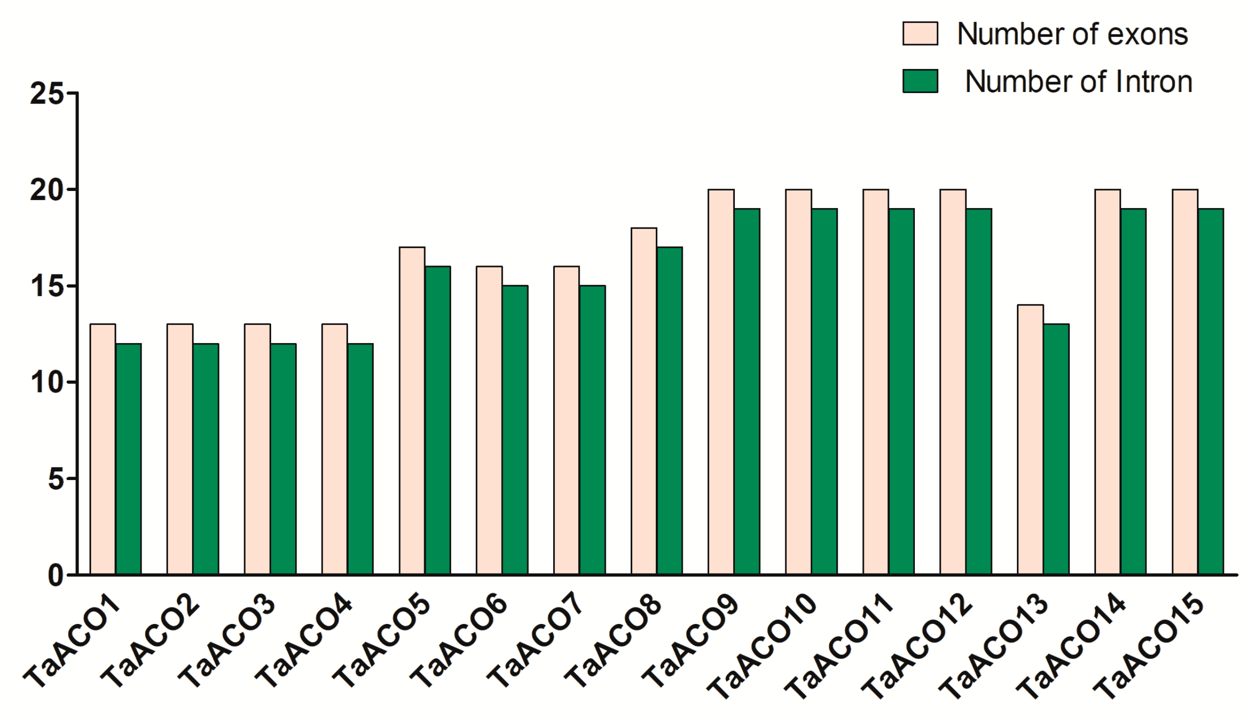

Figure S5. The different number of exons and introns in found in the TaACOs gene family.

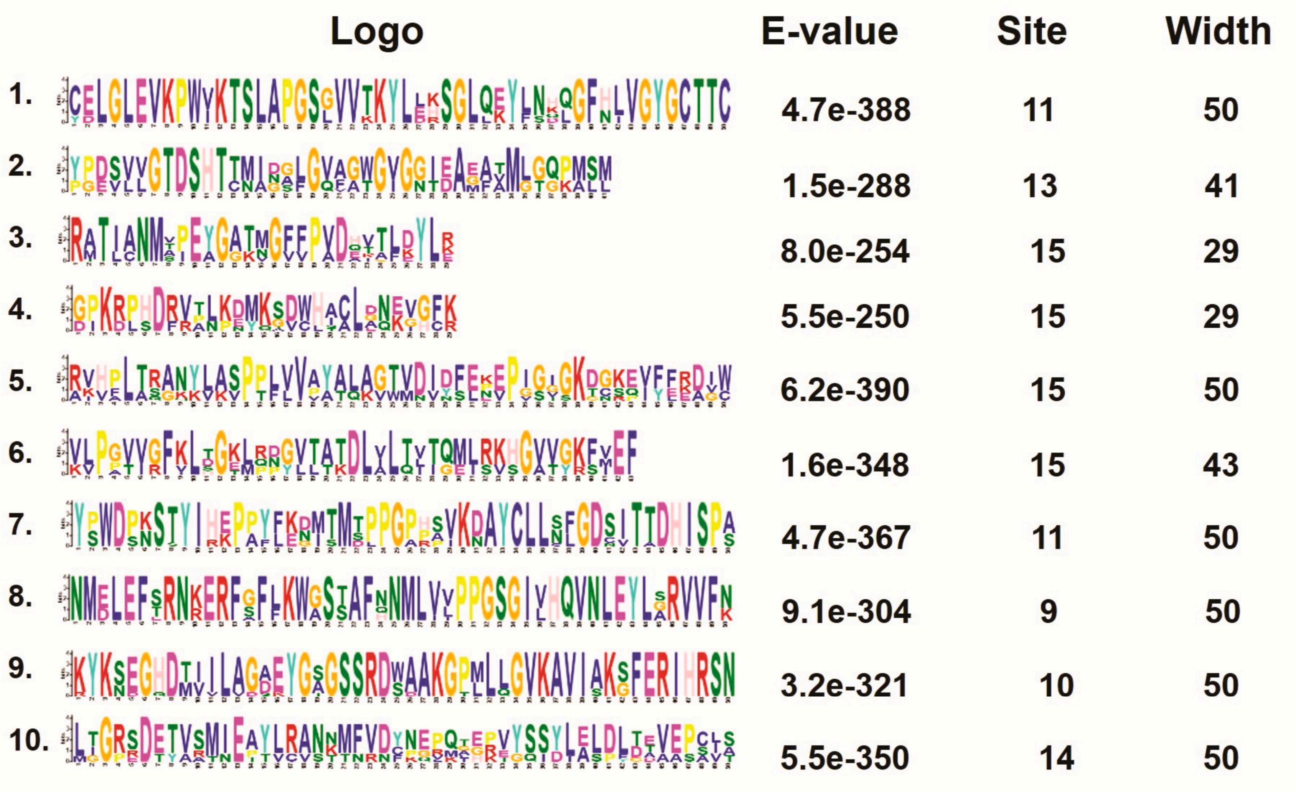

Figure S6. Sequence logo of TaACO motif and height of each mountain indicate the conservation at this position, whereas the height of the different letters represent the frequency of the corresponding amino acids.

```
                 *       200         *       220         *       240
TaACO1  : -----------------------------MNYSCIIREASMRMINPFVMVSRL------- :
TaACO2  : WEKTSPKLAEIPFKPARLVIMDNTGGPAVVDLAAIRDVIAELDSDPKKINPLVPVDLVID :
TaACO3  : ------------------------------------------------------------ :
TaACO4  : WEKTSPKLAEIPFKPARLVIMDNTGGPAVVDLAAIRDVIAELESDPKKINPLVPVDLVID :
TaACO5  : WEKTSPKLAEIPFKPARLVIMDNTGGPAVVDLAAIRDVIAELESDPKKINPLVPVDVVID :
TaACO6  : -------------------------------MTVLCLCLCALAYIAACIFFQVPVDLVVD :
TaACO7  : WENSATKQVEIPFKPARVLLQDFTGVPAVVDLACMRDAMSKLGSDPNKINPLVPVDLVVD :
TaACO8  : ----------------------------MASISAAAKATSAAFAHKKELAAAAPQQQRLS :
TaACO9  : WENTSPKLAEIPFKPARVLLQDFTGVPAVVDLAAMRDAIAKLGSDANKINPLVPVDLVID :
TaACO10 : ----------------------------MASISAAAKATSAAFAHKKELAAAVPPQQ--- :
TaACO11 : WENTSPKLAEIPFKPARVLLQDFTGVPAVVDLAAMRDAIAKLGSDANKINPLVPVDLVID :
TaACO12 : ----------------------------MASISAAAKATSAAFAHKKELAAAAPQQQRLS :
TaACO13 : WENTSPKLAEIPFKPARVLLQDFTGVPAVVDLAAMRDAIAKLGSDANKINPLVPVDLVID :
TaACO14 : WEKTSLEQVEIPFKPSRVILQDFTGVPVLVDFASMRDAMLELGGNPDKINPMVPADLVID :
TaACO15 : WEKTSLEQVEIPFKPSRVILQDFTGVPVLVDLASMRDAMSELGGNPDKINPMVPADLVID :

                 *       260         *       280         *       300
TaACO1  : -DFWSRRTRAGRVHAVATSTGLPRA--------------------PASTGSVKS------ :
TaACO2  : HSVRVDVAKCADALKQNMDLEFSRNKERFSFFKWASSAFNNMLVLPPGSGILHQVNLEYL :
TaACO3  : ------------------------------------------------------------ :
TaACO4  : HSVRVDVAKCADALKQNMDLEFSRNKERFSFFKWASSAFNNMLVLPPGSGILHQVNLEYL :
TaACO5  : HSVRVDVAKCADALKQNMDLEFSRNKERFSFFKWASSAFNNMLVLPPGSGILHQVNLEYL :
TaACO6  : HSVQVDVARSENAVQANMELEFSRNKERFGFLKWGSTAFNNMLVVPPGSGIVHQVNLEYL :
TaACO7  : HSVQVDVARSENAVQANMELEFSRNKERFGFLKWGSTAFNNMLVVPPGSGIVHQVNLEYL :
TaACO8  : AGASSRRARAGRVRAVATPTRAPRS--------------------PASTGSVKS------ :
TaACO9  : HSVQVDVARSSNALQSNMELEFTRNRERFGFLKWGSTAFQNMLVVPPGSGIVHQVNLEYL :
TaACO10 : -LARSRRARAGRVRAVATPTRAPRS--------------------PASTGSVKS------ :
TaACO11 : HSVQVDVARSTNALQSNMELEFTRNRERFGFLKWGSTAFHNMLVVPPGSGIVHQVNLEYL :
TaACO12 : AGASSRRARAGRVRAVATPTRAPRS--------------------PASTGSVKS------ :
TaACO13 : HSVQVDVARSSNALQSNMELEFTRNRERFGFLKWGSTAFQNMLVVPPGSGIVHQVNLEYL :
TaACO14 : HSVTANVVRSQNAIQANMELEFERNKERFAFLKWGSSAFHNMLIIPPGSGIVHQVNLEYL :
TaACO15 : HSVTANVVRSQNAIQANMELEFERNKERFAFLKWGSSAFHNMLIIPPGSGIVH------- :

                 *       320         *       340         *       360
TaACO1  : ------------------------------------------------PMTTTEKILARK :
TaACO2  : SRVVFKADGVLYPDSVVGTDSHTTMINSLGVAGWGVGGIEAMAAMLGQPMSMVLPGVVGF :
TaACO3  : ---------------------------------------------------MVLPGVVGF :
TaACO4  : SRVVFKADGVLYPDSVVGTDSHTTMINSLGVAGWGVGGIEAMAAMLGQPMSMVLPGVVGF :
TaACO5  : SRVVFKADGVLYPDSVVGTDSHTTMINSLGVAGWGVGGIEAMAAMLGQPMSMVLPGVVGF :
TaACO6  : ARVVFNNGGILYPDSVVGTDSHTTMIDGLGVAGWGVGGIEAEATMLGQPMSMVLPAVVGF :
TaACO7  : ARVVFNNGGILYPDSVVGTDSHTTMIDGLGVAGWGVGGIEAEATMLGQPMSMVLPAVVGF :
TaACO8  : ------------------------------------------------PMTTTEKILARA :
TaACO9  : GRVVFNTDGIMYPDSVVGTDSHTTMIDGLGVAGWGVGGIEAEATMLGQPMSMVLPGVVGF :
TaACO10 : ------------------------------------------------PMTTTEKILARA :
TaACO11 : GRVVFNTDGIMYPDSVVGTDSHTTMIDGLGVAGWGVGGIEAEATMLGQPMSMVLPGVVGF :
TaACO12 : ------------------------------------------------PMTTTEKILARA :
TaACO13 : GRVVFNTDGIMYPDSVVGTDSHTTMIDGLGVAGWGVGGIEAEATMLGQPMSMVLPGVVGF :
TaACO14 : ARVVFNRDGLLYPDSVLGTDSHTTMINGLGVVGWGVGGIDAEAAMLGQPMSMVLPGVVGF :
TaACO15 : -----------------------------------------------QPMSMVLPGVVGF :
```

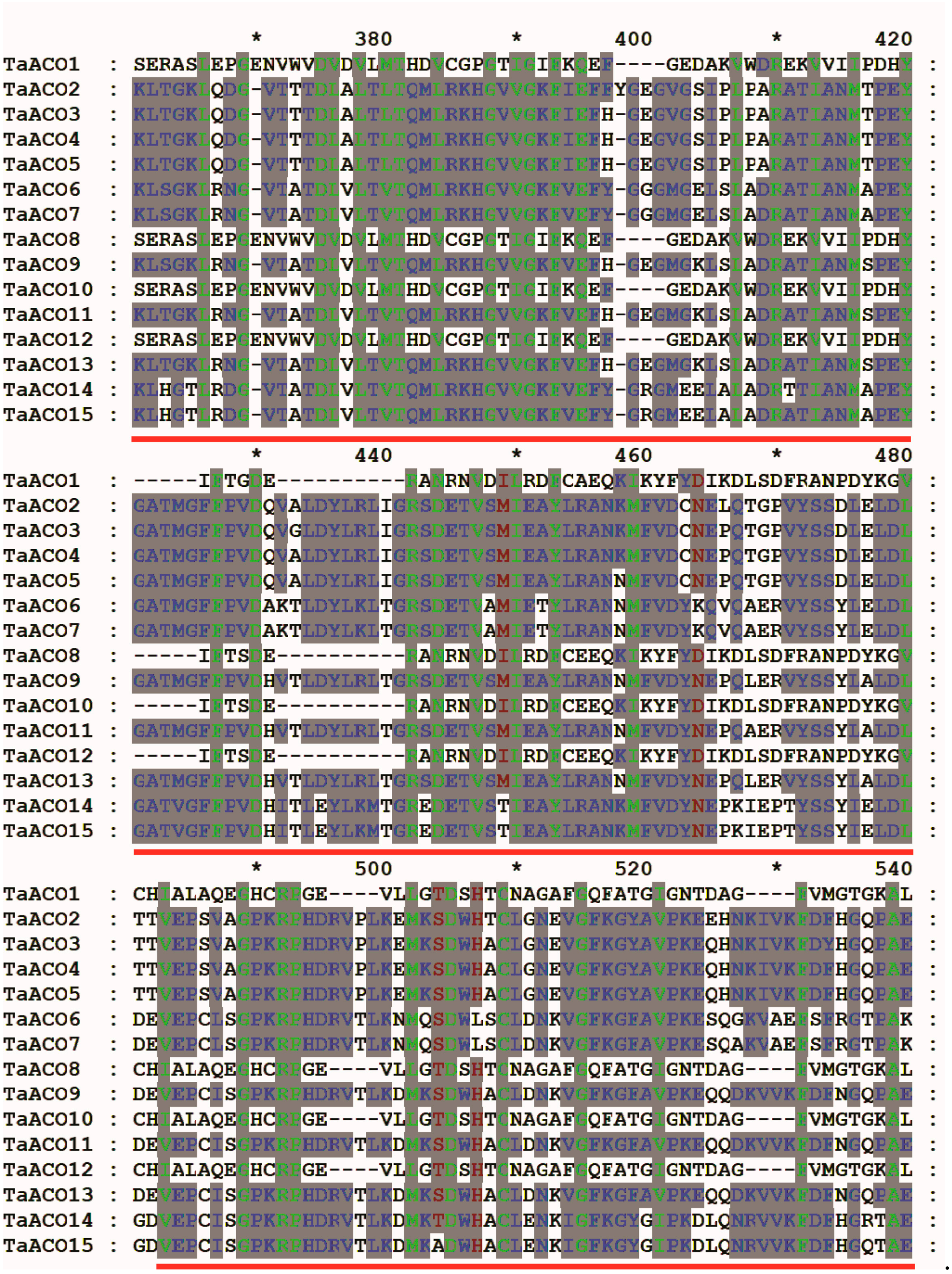

380
400
420
440
460
480
500
520
540
TaACO1
TaACO2
TaACO3
TaACO4
TaACO5
TaACO6
TaACO7
TaACO8
TaACO9
TaACO10
TaACO11
TaACO12
TaACO13
TaACO14
TaACO15

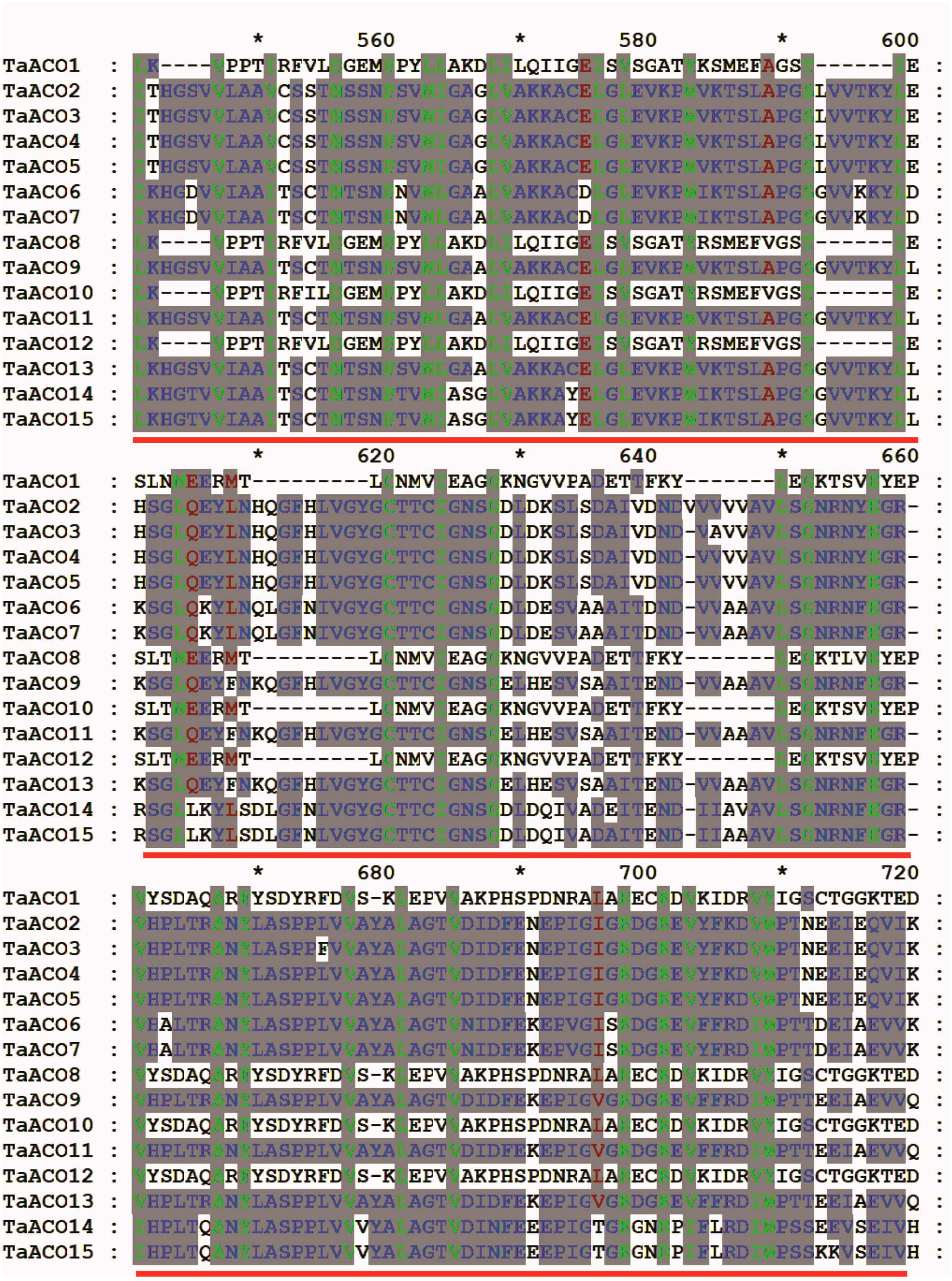

560
580
600
620
640
660
680
700
720
TaACO1
TaACO2
TaACO3
TaACO4
TaACO5
TaACO6
TaACO7
TaACO8
TaACO9
TaACO10
TaACO11
TaACO12
TaACO13
TaACO14
TaACO15

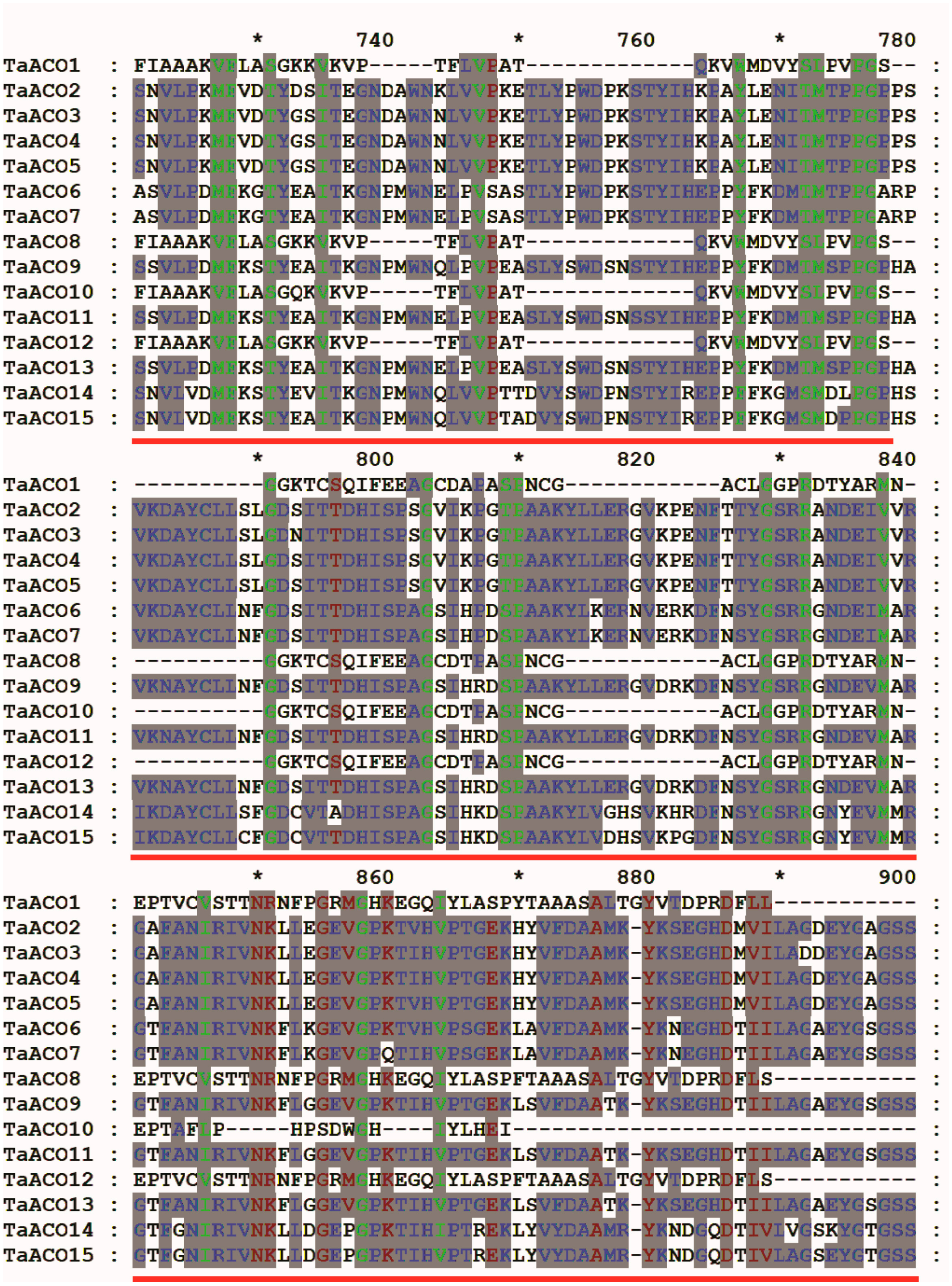

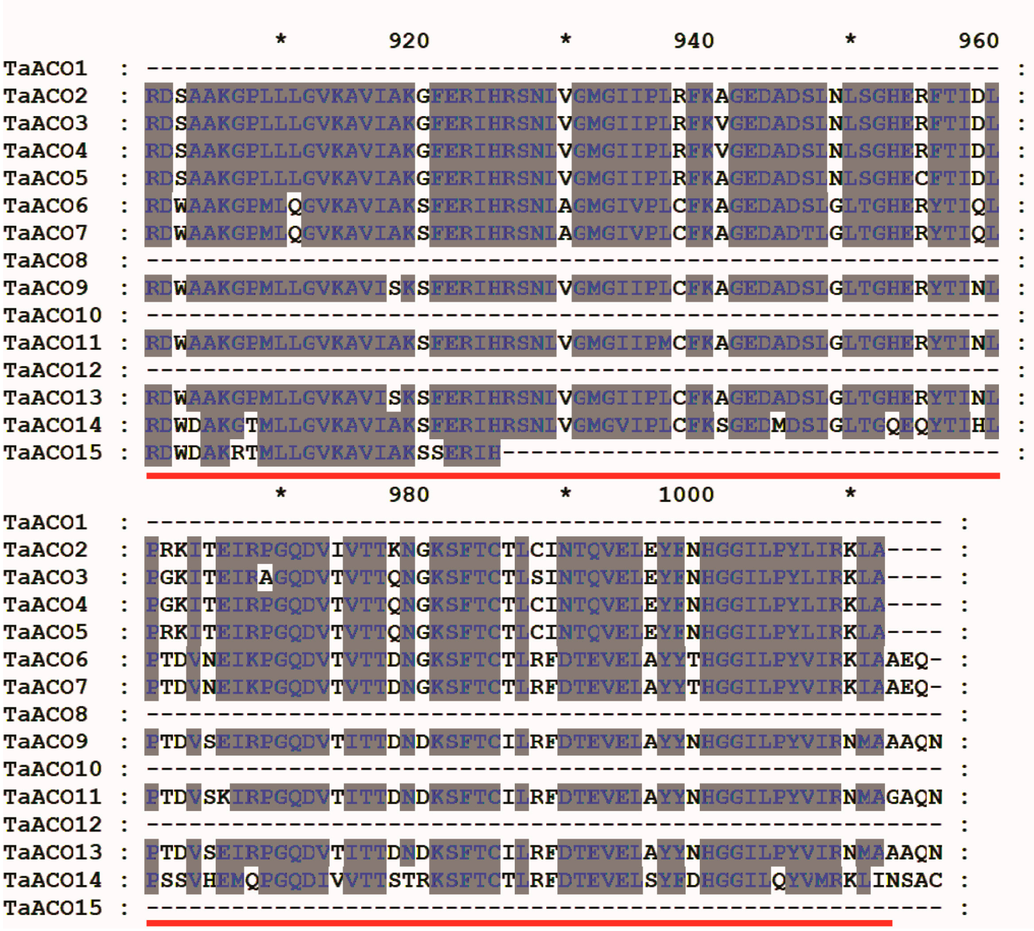

Figure S7. Alignment of the TaACO proteins and preserved aconitase domain is indicated with red color line. Amino acid residues conserved in all proteins were shaded blue and similar amino acids were gray shaded. Dashes denotes gaps led to exploit the alignment of the homologous region.

A

```
                  *        20         *        40         *        60         *
AtACO1   : ---------------------------------------------------------------------- :
AtACO2   : ---------------------------------MYRRATSGVRSASARLSSSLSRIASSETASVSAPSAS :
AtACO3   : -----------------------------------MYLTASSSASSSIIRAASSRSSSLFSFRSVLSPSV :
AtACO4   : ---------------------------------------------------------------------- :
OsACO1   : -----------------------------------MPPLTSALLSRSSSTRIPAAAAAAAAISNPAGAAA :
OsACO2   : ---------------------------------------------------------------------- :
OsACO3   : -----------------------------------MYKAAYSSAS--ARLSSSLLRFRSLPSPAPSSSLS :
OsACO4   : ---------------------------------------------------------------------- :
GmACO1   : -----------------------------------MYITTASSSASSLLRATRPKLFFPSPSRNFASFTP :
GmACO2   : ----------------------------------------MASTVSSILRASRSKLSSSSSASLSRTLAR :
GmACO3   : ---------------------------------------------MRVLSLTR---FFSSP--------- :
GmACO4   : ---------------------------------------------------------------------- :
GmACO5   : ---------------------------------------------------------------------- :
GmACO6   : ---------------------------------------------------------------------- :
GmACO7   : ---------------------------------------------------------------------- :
GmACO8   : ---------------------------------------------------------------------- :
TaACO1   : ---------------------------------------------------------------------- :
TaACO2   : ---------------------MASTAGLQTSHGHWFNTTLTSSQSHLGFRSSFPRPPAGDFLERSPPRCR :
TaACO3   : ---------------------------------------------------------------------- :
TaACO4   : ---------------------MASTAGLQISHGHWFNTTLTSSQSHPCSRSSFPRPPAGDYLERSPPRCR :
TaACO5   : ---------------------MASTAGLQTSHGHWFNTTLTSSQSHLCFRYSFPRPPAGDFLECSPPRRS :
TaACO6   : ---------------------------------------------------------------------- :
TaACO7   : -----------------------------------MPPLASSLLLSRSAAGPGSARATAAAAAAISRPAA :
TaACO8   : ---------------------------------------------------------------------- :
TaACO9   : -----------------------------------MKLLPPPPAAPCCS-SDPSSHHRRLPPPPQL---- :
TaACO10  : ---------------------------------------------------------------------- :
TaACO11  : -----------------------------------MKPLPTPPASSCCSSPDPYSHHRRLPPPPQP---- :
TaACO12  : ---------------------------------------------------------------------- :
TaACO13  : -----------------------------------MKLLPTPPAS-----SDPYSHHR-LPPPPQL---- :
TaACO14  : ---------------------------------------------------------------------- :
TaACO15  : ---------------------------------------------------------------------- :
PpACO1   : ---------------------------------------------------------------------- :
PpACO2   : ------------------MQILTYVCRSLQAQRFCPRLPHVHGLRASSTASPVGLTRFGNSSFLAPPSQP :
PpACO3   : MRLSRLPLPPRLPDRSFTMPLFMYVCRSLRQHGCSSKFPLSLGHRAASSAASAGSIRSGVPSFRSTPSQP :
PpACO4   : ---------------------------------------------------------------------- :
PpACO5   : --------------------MLPLLHSGSHPRRFAPPL--------SSHAPPR-----SLPHFLS--SLK :
SdACO1   : ---------------------------------------------------------------------- :
SdACO2   : ---------------------------------------------------------------------- :
SdACO3   : ---------------------------------------------------------------------- :
SdACO4   : ---------------------------------------------------------------------- :
SdACO5   : ---------------------------------------------------------------------- :
SdACO6   : ----------------------------------------------MLAAAWNLCLAAPIVRKDSGLSPP :
SdACO7   : ----------------------------------------------MLAAAWNLCLAAPIVRKDSGLSPP :
```

```
                    80         *       100         *       120         *       140
AtACO1    : ------------------------------------------------------------MASENPFRSI :
AtACO2    : SLRNQTNRSKSFSSALRSFRVCSASTRWSHGGSWGSPASLRAQARNSTPVMEKFERKYATMASEHSYKDI :
AtACO3    : SSTSPSSLLARRSFGTISPAFRRWSHSFHSKPSPFRFTSQIRAV---SPVLDRLQRTFSSMASEHPFKGI :
AtACO4    : -------------------------------------------MASVISSSPFL---------CKSSSKS :
OsACO1    : SSSSPSPPPPSSRPR--------PASPFASGLAGRIFGGRRAAARSSSSAAAVFERRFASAAAKNSYDEI :
OsACO2    : ---------------------------------------------------------------------- :
OsACO3    : RLLSLRARAFSRPSRGGWAAAGGWSGRASSSPVVGCGACRAQIGAVAPAVERVHRRMAATAAAEHPFKNI :
OsACO4    : -------------------------------------------MASISAASPVAG-------KAAAFAHK :
GmACO1    : CTS-----SFSATARSLLCSVPRWSHRLHSA-SPLTPRPRISAV---APLVERFHREIATMANENPFKGN :
GmACO2    : SAPRRSPGSSSAATRSFGSAVPRWSHGVDWR-SPLGLRPQIRAA---APLIERFHRRIATSATDNPFKGN :
GmACO3    : ---------------------------------------------------------------------- :
GmACO4    : -------------------------------------------MRIYDEFK------------EIVKIQK :
GmACO5    : -------------------------------------------MASSLLSPSFT---------SFPRIKK :
GmACO6    : ---------------------------------------------------------------------- :
GmACO7    : ---------------------------------------------------------------------- :
GmACO8    : -------------------------------------------MAFNSSP--------------FITCKK :
TaACO1    : --------------------------------------------MNYSCIIREA---------SMRMINP :
TaACO2    : RRCSHAVAAAIVVEKLGQGRSVGPSLFVPRRSRDGFLARRGRQQPAAAPCGSAVEGAGAAAPATDRFANT :
TaACO3    : ---------------------------------------------------------------------- :
TaACO4    : RRCSHAVAAAIVVEKLGQRRSVGPSLFVPRRSRDGFLARRGRQQPAAAPCGSAVEGAG-AAPATDRFAKT :
TaACO5    : RRCSHAVAAAIVVEKLGQGRSLGPSLFVSRRSRDGFLARRGRQQLAAAPCGSAVEGAG-AAPATDRFAKT :
TaACO6    : ---------------------------------------------------------------------- :
TaACO7    : AEPAPCPSAPAPSPLHAARGGQGPRGAFASGLAGRLFGGRRAAARSSSSAAAVFERRFASAATKNSYDEI :
TaACO8    : -------------------------------------------MASISAAAKAT---------SAAFAHK :
TaACO9    : RRAAFPP-AASRPFAGLLRAAR---VRCSSS-TSGRGRG---------SVARFQRSSMAASGTEHAYNNI :
TaACO10   : -------------------------------------------MASISAAAKAT---------SAAFAHK :
TaACO11   : RRAAFPP-PAPRPFAGLLRAAR---ARCSPSPSATTGPA----------VERSQRSRMAASGTEHAYSNI :
TaACO12   : -------------------------------------------MASISAAAKAT---------SAAFAHK :
TaACO13   : RRASFPP-AASRPFAGLLRAAR---VRCSSSSAAGRGRGSVAP-----AVARFQRR-MAVSGTEHAYSNI :
TaACO14   : -MSTSSSNSNPFSFALR----------------------------------------------------- :
TaACO15   : ---------------------------------------------------------------------- :
PpACO1    : ---------------------------------------------------------------------- :
PpACO2    : RL-SSPVINSRLFRTLAAASY-PDEHRPADFSVARS-----TKSTVGLRSTIPGARMASTQASENPFSSL :
PpACO3    : RL-SPDSAGSRLFRTIVSASYSADECRAAVASVGRSSPRALSPTMVPTTTTVPGSRMASTQASENPFSSL :
PpACO4    : -------------------------------------------MATTAVSVAHHPGISSALRDGASLASG :
PpACO5    : FL-CPASFHSVTILILLLSPP--------------------PPSPLPFP-----STMASSPASKNPFSNL :
SdACO1    : -------MHSQELKYFSLDSF----------------------------------------FAANPYKNL :
SdACO2    : -------MHSQELKYFSLDSF----------------------------------------FAANPYKNL :
SdACO3    : -------------------------------------------------------------------MEG :
SdACO4    : ---------------------------------------------------------------------- :
SdACO5    : ---------------------------------------------------------------------- :
SdACO6    : RLSCFHRVRSVHFRASIALRQ----------------------------------RQEIAMVSGHPFESI :
SdACO7    : RLSCFHRVRSVHFRASIALRQ----------------------------------RQEIAMVSGHPFESI :
```

```
                       *        160         *        180         *        200         *
AtACO1   : LKALEKPDGG-EFGNYYSLPALNDPRIDKLPYSIRILLESAIRNCDEFQVKSKDVEKILDWENTSPKQVE :
AtACO2   : LTSLPKPGGG-EYGKYYSLPALNDPRIDKLPFSVRILLESAIRNCDNYQVTKDDVEKILDWENTSTKQVE :
AtACO3   : FTTLPKPGGG-EFGKFYSLPALNDPRVDKLPYSIRILLESAIRNCDNFQVTKEDVEKIIDWEKTSPKQVE :
AtACO4   : DLGISSFPK--SSQISIHRCQKKSISRKIVSVMAPQKD-RSPGTTGSVKTGMTMTEKILARASEKSLVVP :
OsACO1   : LTGLAKPGGGAEFGKYYSLPALSDPRIERLPYSIRILLESAIRNCDEFQVTGKDVEKILDWENSAPKQVE :
OsACO2   : ---------------------------------------------------------------------- :
OsACO3   : LTTLPKPGGG-EYGKFYSLPALNDPRIDKLPYSIRILLESAIRNCDNFQVNQNDVEKIIDWENTSPKLAE :
OsACO4   : NELAAAAVAPSQQQLQRRVSGRRARSGRVRAVATPARAPRAPSSTGSVKSAMTMTEKILARASERASLEP :
GmACO1   : LTSLPKPGGG-EFGKFYSLPSLNDPRIDRLPYSIRILLESAIRNCDNFQVKKEDVEKILDWENNSTKQVE :
GmACO2   : LTSLPKPGGG-EFGKFYSLPSLNDPRIDRLPYSIRILLESAIRNCDNFQVKKEDVEKIIDWENSSVKQVE :
GmACO3   : ---------------------------------------------------------------------- :
GmACO4   : FRRLVSYSG-----FYCFATLISYFYVNNTTRAGFSRADQFYASYPAGTELLTDTTKLYKAALGNCFEAE :
GmACO5   : DVSLSAFTS---------QRCFKALPRRIIRCAVAAPQ-RQPSTTGSVRTAMTMTEKILARASEKVQLTP :
GmACO6   : ---------------------------------------------------------------------- :
GmACO7   : ---------------------------------------------------------------------- :
GmACO8   : DLGFFAFPSP-SSIIGKRQKCKKTSSNKIVSVVSSPKNKRSPSATGSTKIPMTATEKILARAAEKCEVRP :
TaACO1   : FVMVSRL----------DFWSRRTRAGRVHAVATSTGLPRAPASTGSVKSPMTTTEKILARKSERASLEP :
TaACO2   : LTSLPKPGGG-EYGKFYSIPALNDPKIDKLPYCIRILLESAVRNYDNFQVTESDVQNIIDWEKTSPKLAE :
TaACO3   : ---------------------------------------------------------------------- :
TaACO4   : LTNLPKPGGG-EYGKFYSIPALNDPKIDKLPYCIRILLESAVRNYDNFQVTESDIQNIIDWEKTSPKLAE :
TaACO5   : LTILPKPGGG-EYGKFYSIPALNDPKIDKLPYCIRILLESAVRNYDNFQVTESDVQNIIDWEKTSPKLAE :
TaACO6   : ---------------------------------------------------------------------- :
TaACO7   : LTSLAKPGGGADFGKYYSLPALADPRIDRLPYSIRILLESAIRNCDEFQVTGKDVEKILDWENSATKQVE :
TaACO8   : KELAAAAPQ--QQRLSAGASSRRARAGRVRAVATPTRAPRSPASTGSVKSPMTTTEKILARASERASLEP :
TaACO9   : LTSLPKPEGG-EYGKFYSLPALNDPRIDKLPYSIRILLESAIRNCDGFQVTKNDVEKIIDWENTSPKLAE :
TaACO10  : KELAAAVPP--QQ----LARSRRARAGRVRAVATPTRAPRSPASTGSVKSPMTTTEKILARASERASLEP :
TaACO11  : LTSLPKPEGG-EYGKFYSLPALNDPRIDKLPYSIRILLESAIRNCDGFQVTKNDVEKIIDWENTSPKLAE :
TaACO12  : KELAAAAPQ--QQRLSAGASSRRARAGRVRAVATPTRAPRSPASTGSVKSPMTTTEKILARASERASLEP :
TaACO13  : LTSLPKPEGG-EYGKFYSLPALNDPRIDKLPYSIRILLESAIRNCDGFQVTKNDVEKIIDWENTSPKLAE :
TaACO14  : --GLRDREGG-DCGSYYSIPDLGDQRIDKLPYTIRVLLESAVRNCDEFQITKEDVEKIMGWEKTSLEQVE :
TaACO15  : ----------------------------------------------------------MGWEKTSLEQVE :
PpACO1   : -------------------------------------------------MKADVEKIIDWEKTSSKQVE :
PpACO2   : ITDLPKPDGG-SYGKFYSLVKLNDPRVDSLPYSIRYLLEAAIRNCDNFQVTKEDVEKIVDWEKTAPKQVE :
PpACO3   : ITDLPKPDGG-SYGKFYSLVKLNDPRVDSLPYSIRYLLEAAIRNCDNFQVTKEDVEKIVDWEKTAPKQVE :
PpACO4   : SGFYGVRVS--VAAAPKTSVASAHRDMRVRAVMAPQKQERSPASTGAVKQAMTATEKILANASEKTKLAP :
PpACO5   : VTDLPKASGG-SYGQYYSLVKLNDPRVDELPYSIRYLLESAIRNCDNFQVLEADVEKILDWKVTAPKQVE :
SdACO1   : VKPLEG------HGKYYSLPALNDPRVDKLPYSIKILLESCIRNCDNFQVTKEDVEKIIDWENTAPQQVE :
SdACO2   : VKPLEG------HGKYYSLPALNDPRVEKLPYSIKILLESCIRNCDNFQVTKEDVEKIIDWENTAPQQVE :
SdACO3   : AMLHSSYFS--VNARPMS-----RRSSTISAVMAPPQQQRPPASTGAVKHAMTLTEKILARASEKTKLVP :
SdACO4   : ---------------------------------------------------------------------- :
SdACO5   : -MLHSSYFS--VNARPMS-----RRSSTISAVMAPPQQQRAPASTGAVKHAMTLTEKILARASEKTKLVP :
SdACO6   : LTSLSKDDGG-EYGKYYSLPDLHDPRIEKLPYSIKILLESAIRNCDNFQVTKDDVEKIADWVNTAPKLVE :
SdACO7   : LTSLSKDDGG-EYGKYYSLPDLHDPRIEKLPYSIKILLESAIRNCDNFQVTKDDVEKIADWVNTAPKLVE :
```

```
                      220         *        240         *        260         *        280
AtACO1   : IPFKPARVLLQDFTGVPAVVDLACMRDAMNNLGGDSNKINPLVPVDLVIDHSVQVDVARSENAVQANMEL :
AtACO2   : IAFKPARVILQDFTGVPVLVDLASMRDAVKNLGSDPSKINPLVPVDLVVDHSIQVDFARSEDAAQKNLEL :
AtACO3   : IPFKPARVLLQDFTGVPAVVDLACMRDAMNKLGSDSNKINPLVPVDLVIDHSVQVDVARSENAVQANMEL :
AtACO4   : GDNIWVNVDVLMTHDVCGPGAFGIFKREFGEK----AKVWDPEKIVVIPDHYIFTADKRANRNVDIMREH :
OsACO1   : IPFKPARVLLQDFTGVPAVVDLACMRDAMSKLGSDPNKINPLVPVDLVIDHSVQVDVARSENAVQANMEL :
OsACO2   : -------------------------------------------------------------------MEL :
OsACO3   : IPFKPARVLLQDFTGVPAVVDLAAMRDAMAKLGSDANKINPLVPVDLVIDHSVQVDVARSPNAVQSNMEL :
OsACO4   : GENVWVDVDVLMTHDVCGPGTIGIFKREFGED----AKVWDREKVVIIPDHYIFTSDERANRNVDILRDF :
GmACO1   : IPFKPARVLLQDFTGVPAVVDLACMRDAMNKLGSDSNKINPLVPVDLVIDHSVQVDVTRSDNAVQANMEL :
GmACO2   : IPFKPARVLLQDFTGVPAVVDLACMRDAMNKLGSDSNKINPLVPVDLVIDHSVQVDVARSENAVQANMEL :
GmACO3   : ----------LDFTGIPAVVDLACVRDARNKLGSDSNKINPLVPVDLVINHSVQVDVARSEN-------- :
GmACO4   : EWGPFEFCVMAKHFERQGKSPYAYHAKMFRNT----PKVWDCEKLMFIPDHYIFPNAEHAHYIVDISRKF :
GmACO5   : GDNVWVNVDILMTHDVCGPGSIGIFKREFGDD----AKVWDREKLVIIPDHYIFTSDERANRNVDILRDF :
GmACO6   : ---------------------------------------------------------------------- :
GmACO7   : ---------------------------------------------------------------------- :
GmACO8   : GENAWANVDVLMINDITCPGISGIFKKEFGNT----AKVWDREKIVVIPDHYIFTNDERAHRNVDIARDF :
TaACO1   : GENVWVDVDVLMTHDVCGPGTIGIFKQEFGED----AKVWDREKVVIIPDHYIFTGDERANRNVDILRDF :
TaACO2   : IPFKPARLVIMDNTGGPAVVDLAAIRDVIAELDSDPKKINPLVPVDLVIDHSVRVDVAKCADALKQNMDL :
TaACO3   : ---------------------------------------------------------------------- :
TaACO4   : IPFKPARLVIMDNTGGPAVVDLAAIRDVIAELESDPKKINPLVPVDLVIDHSVRVDVAKCADALKQNMDL :
TaACO5   : IPFKPARLVIMDNTGGPAVVDLAAIRDVIAELESDPKKINPLVPVDVVIDHSVRVDVAKCADALKQNMDL :
TaACO6   : ---------------------MTVLCLCLCALAYIAACIFFQVPVDLVVDHSVQVDVARSENAVQANMEL :
TaACO7   : IPFKPARVLLQDFTGVPAVVDLACMRDAMSKLGSDPNKINPLVPVDLVVDHSVQVDVARSENAVQANMEL :
TaACO8   : GENVWVDVDVLMTHDVCGPGTIGIFKQEFGED----AKVWDREKVVIIPDHYIFTSDERANRNVDILRDF :
TaACO9   : IPFKPARVLLQDFTGVPAVVDLAAMRDALAKLGSDANKINPLVPVDLVIDHSVQVDVARSSNALQSNMEL :
TaACO10  : GENVWVDVDVLMTHDVCGPGTIGIFKQEFGED----AKVWDREKVVIIPDHYIFTSDERANRNVDILRDF :
TaACO11  : IPFKPARVLLQDFTGVPAVVDLAAMRDALAKLGSDANKINPLVPVDLVIDHSVQVDVARSTNALQSNMEL :
TaACO12  : GENVWVDVDVLMTHDVCGPGTIGIFKQEFGED----AKVWDREKVVIIPDHYIFTSDERANRNVDILRDF :
TaACO13  : IPFKPARVLLQDFTGVPAVVDLAAMRDALAKLGSDANKINPLVPVDLVIDHSVQVDVARSSNALQSNMEL :
TaACO14  : IPFKPSRVILQDFTGVPVLVDFASMRDAMLELGGNPDKINPMVPADLVIDHSVTANVVRSQNAIQANMEL :
TaACO15  : IPFKPSRVILQDFTGVPVLVDLASMRDAMSELGGNPDKINPMVPADLVIDHSVTANVVRSQNAIQANMEL :
PpACO1   : IPFKPARVILQDFTGVPCVVDLAAMRDAIKRLGGDPSKINPLVPVDLVIDHSVQVDVARKANALQVNMKL :
PpACO2   : IPFKPARVILQDFTGVPAVVDLAAMRDAITRLGGDPDRINPLVPVDLVIDHSVQVDVARSANALQANMEL :
PpACO3   : IPFKPARVILQDFTGVPAVVDLAAMRDAITRLGGDPDRINPLVPVDLVIDHSVQVDVARSANALQANMEL :
PpACO4   : GENVWVKADVLMTHDVCGPGTIGIFKKEFGQN----AKVWDREKIVLIPDHYIFTSDERANRNVDILRDF :
PpACO5   : IPFKPARVILQDFTGVPAVVDLAAMRDAIERLGGDPDKINPLVPVDLVIDHSVQVDVARSPNALQANMEL :
SdACO1   : IAFKPARVILQDFTGVPAVVDLAAMRDAMKRLGGDPNVINPMIPVDLVIDHSVQVDVARAANALEANMKH :
SdACO2   : IAFKPARVILQDFTGVPAVVDLAAMRDAMKRLGGDPNVINPMIPVDLVIDHSVQVDVARAANALEANMKH :
SdACO3   : GENVWVNADVLMTHDVCGPGTIGIFKKEFGAN----AKVWDREKIVIIPDHYIFTADERANRNVDILRDF :
SdACO4   : -----------MTHNVCGPGPIGIFKKEFGANGEMCPQVWDREKIVIIPDHYIFTADERANRNVDILGDF :
SdACO5   : GENVWVNADVLMTHDVCGPGTIGIFKKEFGAN----AKVWDREKIVIIPDHYIFTADERANRNVDILRDF :
SdACO6   : IPFKPARVILQDFTGVPALVDLAAMRDAMKRLGGDPTKINPLIPVDLVIDHSVQVDVARSANAVASNMQF :
SdACO7   : IPFKPARVILQDFTGVPALVDLAAMRDAMKRLGGDPTKINPLIPVDLVIDHSVQVDVARSANAVASNMQF :
```

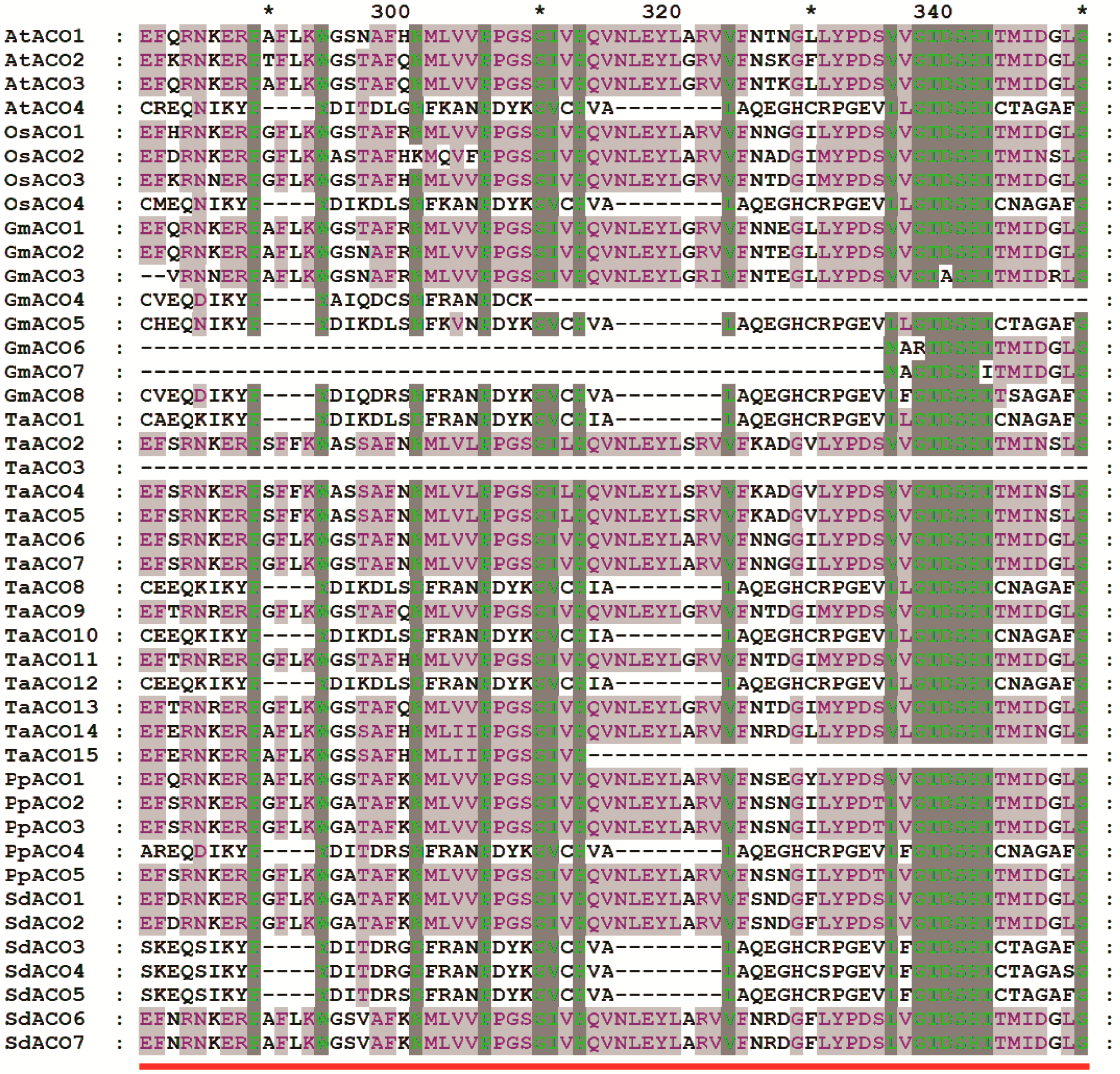

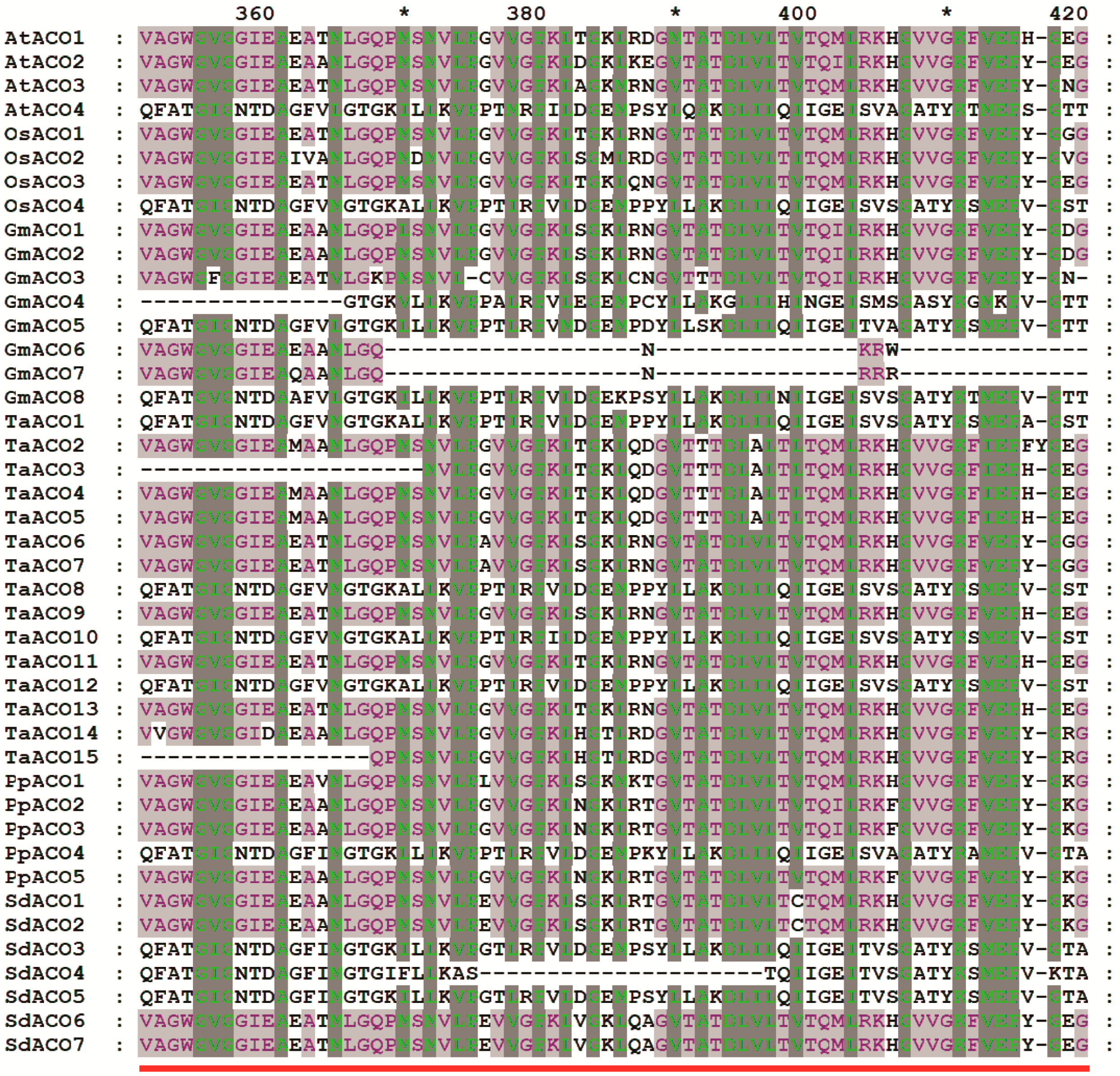
360 * 380 * 400 * 420
AtACO1
AtACO2
AtACO3
AtACO4
OsACO1
OsACO2
OsACO3
OsACO4
GmACO1
GmACO2
GmACO3
GmACO4
GmACO5
GmACO6
GmACO7
GmACO8
TaACO1
TaACO2
TaACO3
TaACO4
TaACO5
TaACO6
TaACO7
TaACO8
TaACO9
TaACO10
TaACO11
TaACO12
TaACO13
TaACO14
TaACO15
PpACO1
PpACO2
PpACO3
PpACO4
PpACO5
SdACO1
SdACO2
SdACO3
SdACO4
SdACO5
SdACO6
SdACO7

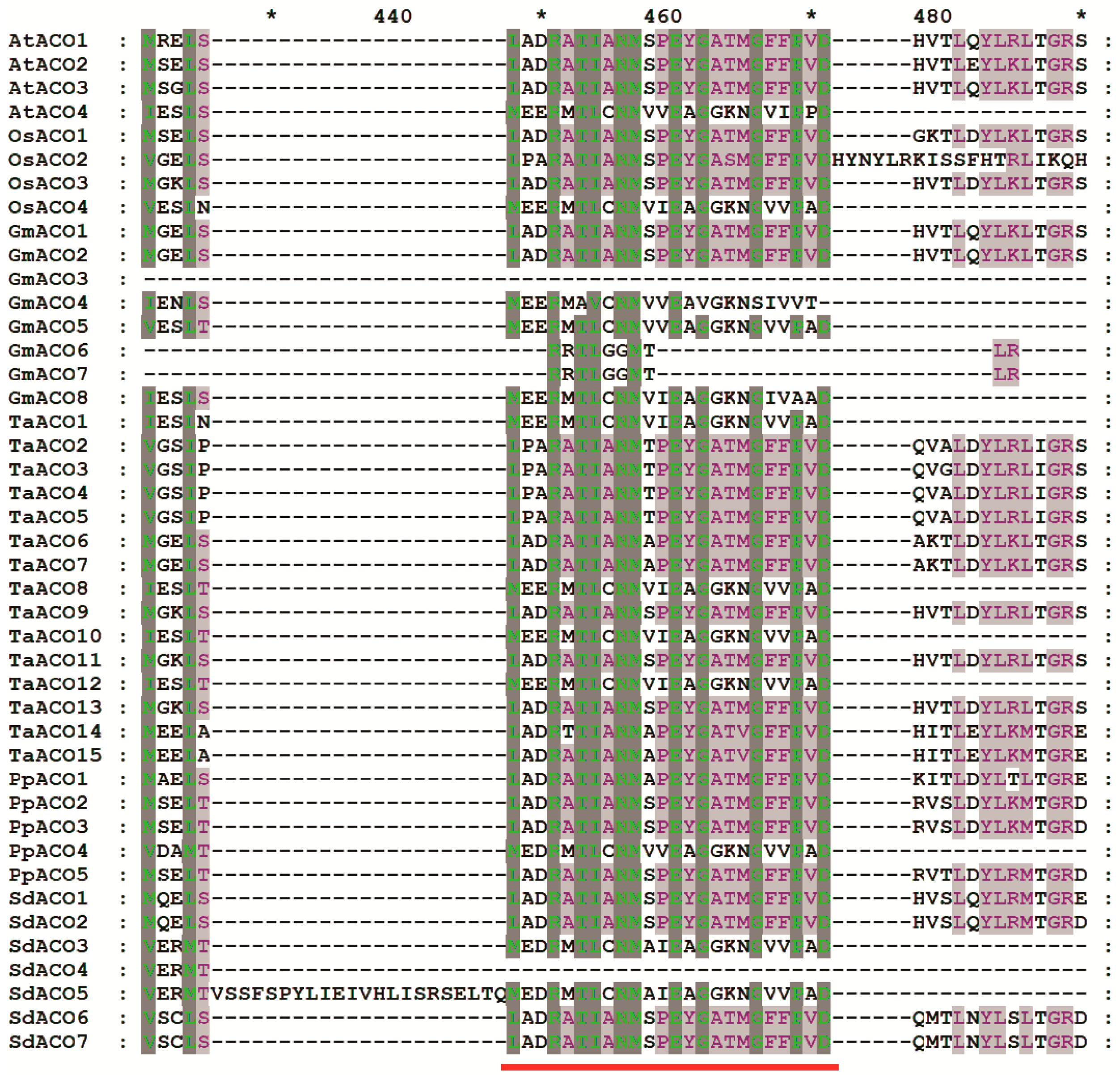

440
460
480
AtACO1
AtACO2
AtACO3
AtACO4
OsACO1
OsACO2
OsACO3
OsACO4
GmACO1
GmACO2
GmACO3
GmACO4
GmACO5
GmACO6
GmACO7
GmACO8
TaACO1
TaACO2
TaACO3
TaACO4
TaACO5
TaACO6
TaACO7
TaACO8
TaACO9
TaACO10
TaACO11
TaACO12
TaACO13
TaACO14
TaACO15
PpACO1
PpACO2
PpACO3
PpACO4
PpACO5
SdACO1
SdACO2
SdACO3
SdACO4
SdACO5
SdACO6
SdACO7

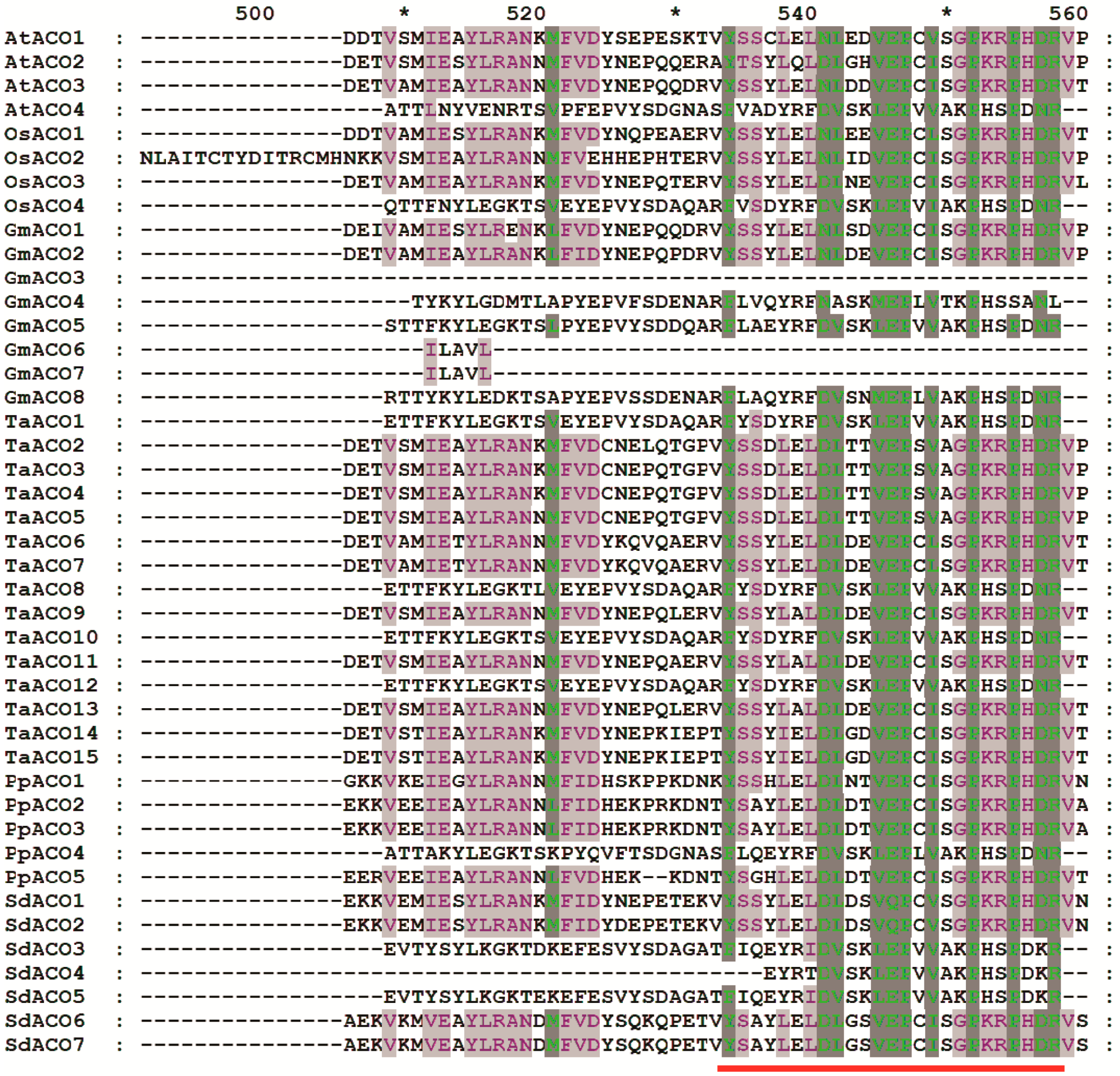
500 * 520 * 540 * 560
AtACO1
AtACO2
AtACO3
AtACO4
OsACO1
OsACO2
OsACO3
OsACO4
GmACO1
GmACO2
GmACO3
GmACO4
GmACO5
GmACO6
GmACO7
GmACO8
TaACO1
TaACO2
TaACO3
TaACO4
TaACO5
TaACO6
TaACO7
TaACO8
TaACO9
TaACO10
TaACO11
TaACO12
TaACO13
TaACO14
TaACO15
PpACO1
PpACO2
PpACO3
PpACO4
PpACO5
SdACO1
SdACO2
SdACO3
SdACO4
SdACO5
SdACO6
SdACO7

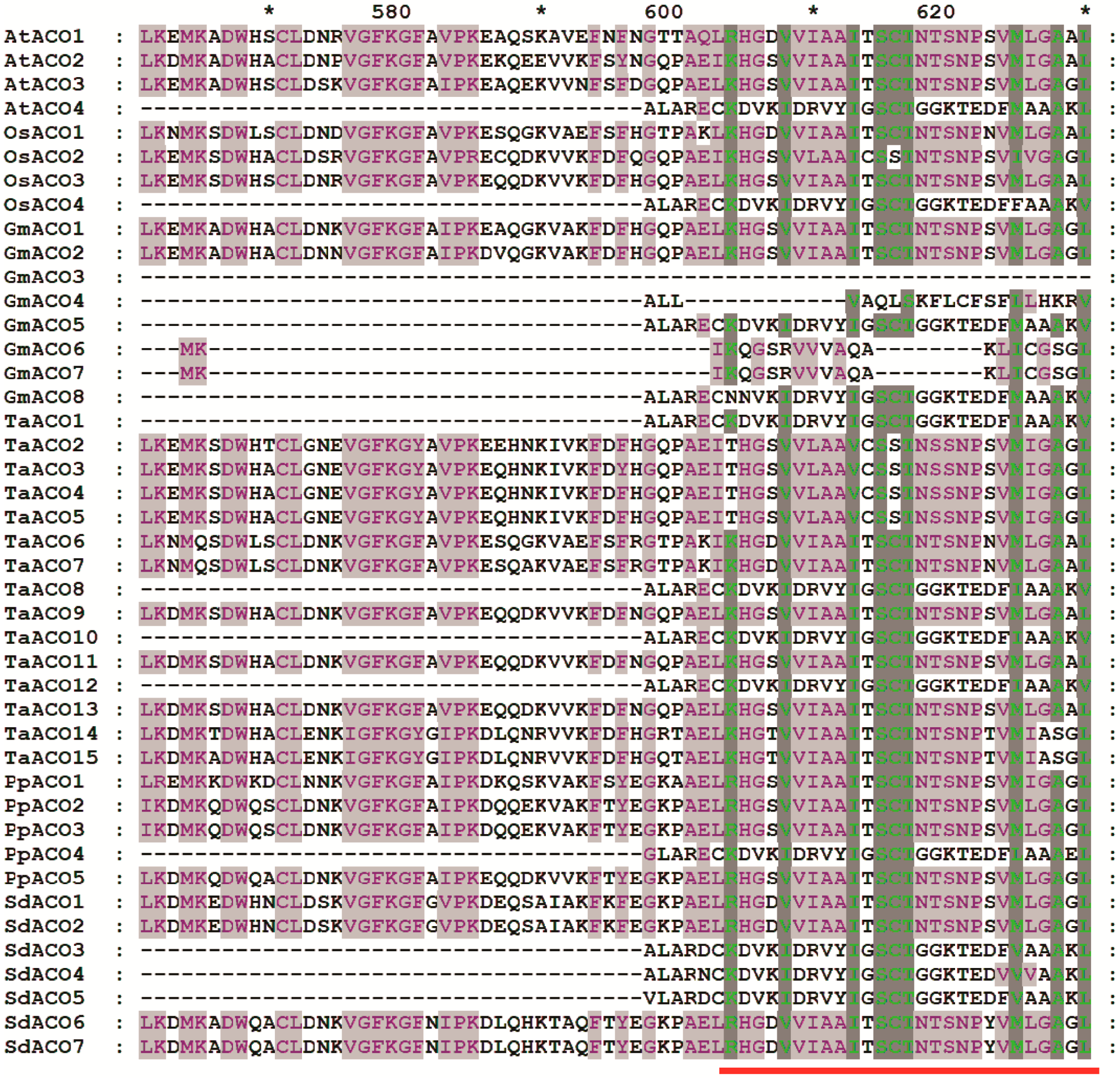
580
600
620
AtACO1
AtACO2
AtACO3
AtACO4
OsACO1
OsACO2
OsACO3
OsACO4
GmACO1
GmACO2
GmACO3
GmACO4
GmACO5
GmACO6
GmACO7
GmACO8
TaACO1
TaACO2
TaACO3
TaACO4
TaACO5
TaACO6
TaACO7
TaACO8
TaACO9
TaACO10
TaACO11
TaACO12
TaACO13
TaACO14
TaACO15
PpACO1
PpACO2
PpACO3
PpACO4
PpACO5
SdACO1
SdACO2
SdACO3
SdACO4
SdACO5
SdACO6
SdACO7

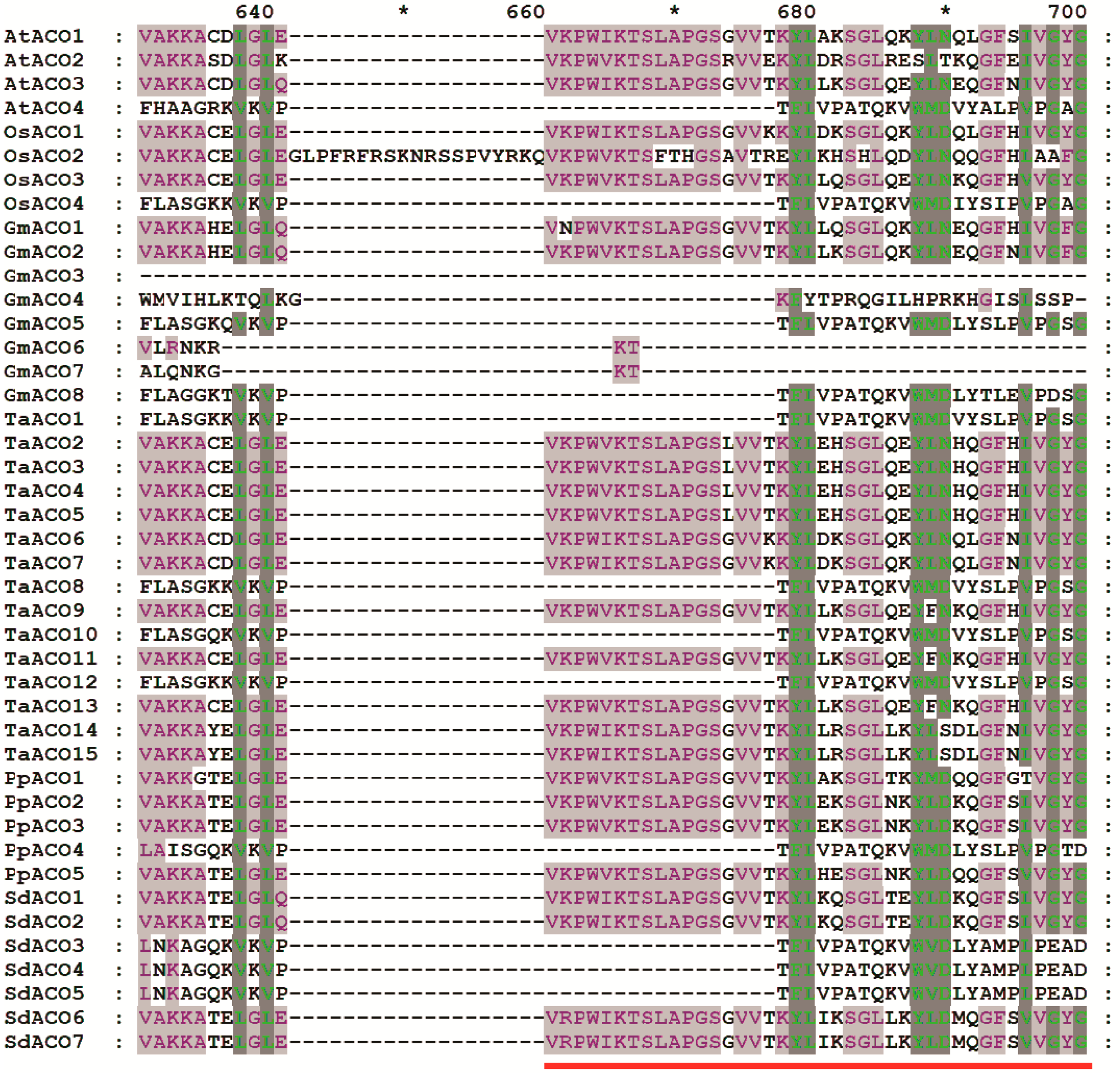
640
660
680
700
AtACO1
AtACO2
AtACO3
AtACO4
OsACO1
OsACO2
OsACO3
OsACO4
GmACO1
GmACO2
GmACO3
GmACO4
GmACO5
GmACO6
GmACO7
GmACO8
TaACO1
TaACO2
TaACO3
TaACO4
TaACO5
TaACO6
TaACO7
TaACO8
TaACO9
TaACO10
TaACO11
TaACO12
TaACO13
TaACO14
TaACO15
PpACO1
PpACO2
PpACO3
PpACO4
PpACO5
SdACO1
SdACO2
SdACO3
SdACO4
SdACO5
SdACO6
SdACO7

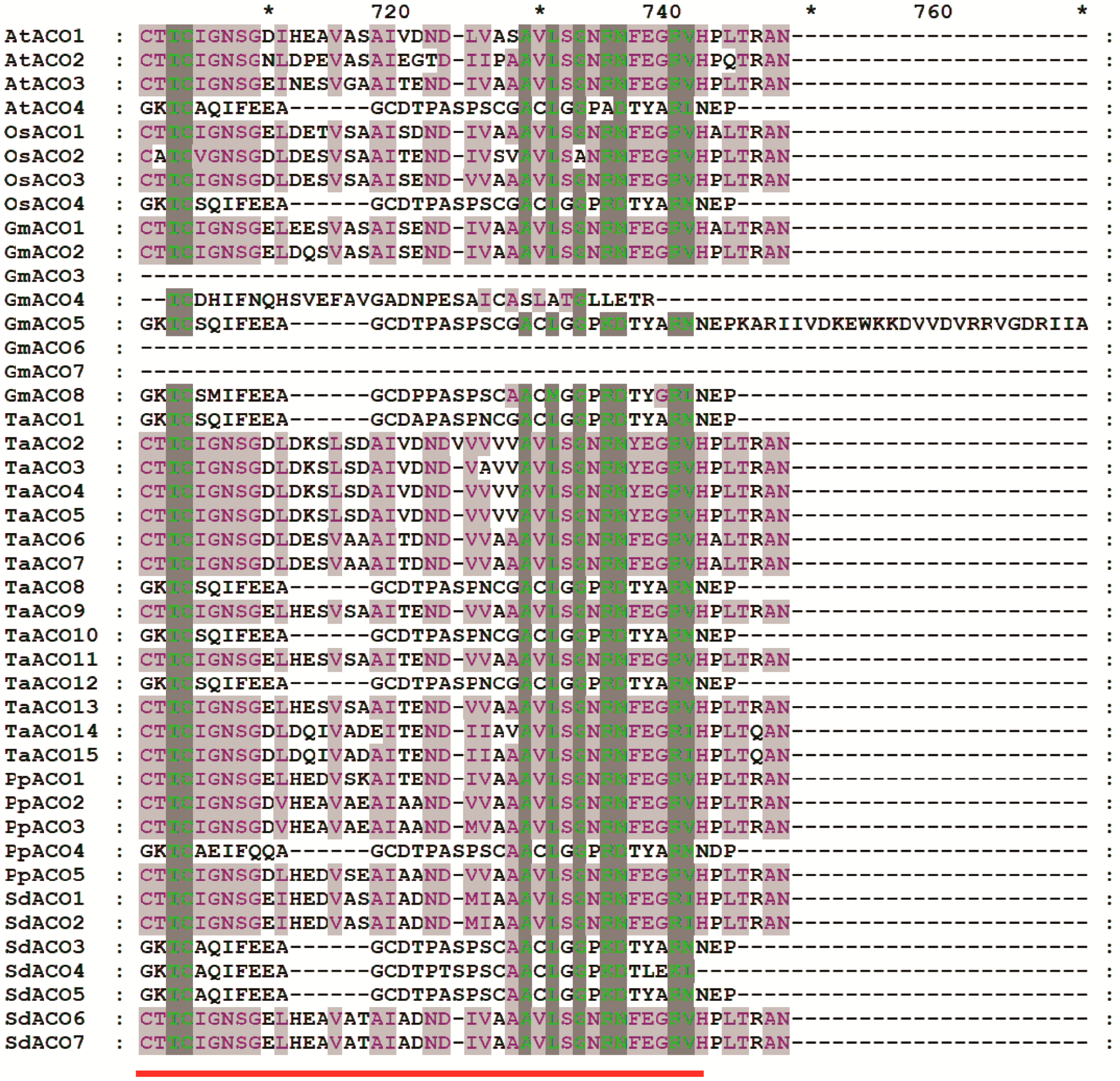
720
740
760
AtACO1
AtACO2
AtACO3
AtACO4
OsACO1
OsACO2
OsACO3
OsACO4
GmACO1
GmACO2
GmACO3
GmACO4
GmACO5
GmACO6
GmACO7
GmACO8
TaACO1
TaACO2
TaACO3
TaACO4
TaACO5
TaACO6
TaACO7
TaACO8
TaACO9
TaACO10
TaACO11
TaACO12
TaACO13
TaACO14
TaACO15
PpACO1
PpACO2
PpACO3
PpACO4
PpACO5
SdACO1
SdACO2
SdACO3
SdACO4
SdACO5
SdACO6
SdACO7

```
                            *        860         *        880         *        900         *
AtACO1   : ----------------YLASPPLVVAYALAGTVDIDFETQPIGTGKDGKQIFFRDIWPSNKEVAEVVQSS :
AtACO2   : ----------------YLASPPLVVAYALAGTVDIDFEKEPIGTRSDGKSVYLRDVWPSNEEVAQVVQYS :
AtACO3   : ----------------YLASPPLVVAYALAGTVNIDFETEPIGKGKNGKDVFLRDIWPTTEEIAEVVQSS :
AtACO4   : ---------------------------------------------------------------------- :
OsACO1   : ----------------YLASPPLVVAYALAGTVNIDFEKEPIGISKDGKEVYFRDIWPSTEEIAEVVKSS :
OsACO2   : ----------------YLASPPLVVAYALAGTVDIDFEKEPIGHGKDGNEVYLRDIWPTNEEIEQVVKSS :
OsACO3   : ----------------YLASPPLVVAYALAGTVDIDFEKEPIGVGKDGKEVFFRDIWPSTEEIAEVVQSS :
OsACO4   : ---------------------------------------------------------------------- :
GmACO1   : ----------------YLASPPLVVAYALAGTVDIDFEKEPIGTGKDGKNVYLRDIWPSTEEIAKVVQSS :
GmACO2   : ----------------YLASPPLVVAYALAGTVDIDFEKEPIGTGKDGNNVYLRDIWPSTQEIAEAVQSS :
GmACO3   : ---------------------------------------------------------------------- :
GmACO4   : ---------------------------------------------------------------------- :
GmACO5   : FGLGEMNGEGKSILEFSEALDLSIANTWFKKREDHLITYKSGGTCSQIDFFLIRKSDRKYCLNCKVIPEE :
GmACO6   : ---------------------------------------------------------------------- :
GmACO7   : ---------------------------------------------------------------------- :
GmACO8   : ---------------------------------------------------------------------- :
TaACO1   : ---------------------------------------------------------------------- :
TaACO2   : ----------------YLASPPLVVAYALAGTVDIDFENEPIGIGKDGKEVYFKDVWPTNEEIEQVIKSN :
TaACO3   : ----------------YLASPPFVVAYALAGTVDIDFENEPIGIGKDGKEVYFKDVWPTNEEIEQVIKSN :
TaACO4   : ----------------YLASPPLVVAYALAGTVDIDFENEPIGIGKDGKEVYFKDVWPTNEEIEQVIKSN :
TaACO5   : ----------------YLASPPLVVAYALAGTVDIDFENEPIGIGKDGKEVYFKDVWPTNEEIEQVIKSN :
TaACO6   : ----------------YLASPPLVVAYALAGTVNIDFEKEPVGISKDGKEVFFRDIWPTTDEIAEVVKAS :
TaACO7   : ----------------YLASPPLVVAYALAGTVNIDFEKEPVGISKDGKEVYFRDIWPTTDEIAEVVKAS :
TaACO8   : ---------------------------------------------------------------------- :
TaACO9   : ----------------YLASPPLVVAYALAGTVDIDFEKEPIGVGKDGKEVFFRDIWPTTEEIAEVVQSS :
TaACO10  : ---------------------------------------------------------------------- :
TaACO11  : ----------------YLASPPLVVAYALAGTVDIDFEKEPIGVGKDGKEVFFRDIWPTTEEIAEVVQSS :
TaACO12  : ---------------------------------------------------------------------- :
TaACO13  : ----------------YLASPPLVVAYALAGTVDIDFEKEPIGVGKDGKEVFFRDIWPTTEEIAEVVQSS :
TaACO14  : ----------------YLASPPLVVVYALAGTVDINFEEEPIGTGKGNRPIFLRDIWPSSEEVSEIVHSN :
TaACO15  : ----------------YLASPPLVVVYALAGTVDINFEEEPIGTGKGNRPIFLRDIWPSSKKVSEIVHSN :
PpACO1   : ----------------YLASPPLVVAYAFAGTVDIDFAKEPIGKSKGGKDVFLKDVWPSNEEIAKVVQSS :
PpACO2   : ----------------YLASPPLVVAYAFAGTVNIDFEKDPIGVGKDGKNVFLRDIWPSNQEVAEVVATS :
PpACO3   : ----------------YLASPPLVVAYAFAGTVNIDFEKDPIGVGKDGKNVFLRDIWPSNQEVAEVVATS :
PpACO4   : ---------------------------------------------------------------------- :
PpACO5   : ----------------YLASPPLVVAYAFAGTVNIDFETEPIGLGKDGKNVFLRDIWPSSDEVAEVVANA :
SdACO1   : ----------------YLASPPLVVAYALAGTVDIDFDTEPIGVGKSGKEVFLRDIWPSSEEVAKVVEKA :
SdACO2   : ----------------YLASPPLVVAYALAGTVDIDFDTEPIGVGKSGKEVFLRDIWPSSEEVAKVVEKA :
SdACO3   : ---------------------------------------------------------------------- :
SdACO4   : ---------------------------------------------------------------------- :
SdACO5   : ---------------------------------------------------------------------- :
SdACO6   : ----------------YLASPPLVVAYALAGTVNIDFEKEPIGVGHSGKQVFLRDIWPTSEEVAKVAEAS :
SdACO7   : ----------------YLASPPLVVAYALAGTVNIDFEKEPIGVGHSGKQVFLRDIWPTSEEVAKVAEAS :
```

```
                       920         *        940         *        960         *        980
AtACO1    : VLPDMFKATYEAITKGNSMWNQLSVA--SGTLYEWDPKSTYIHEPPYFKGMTMSPPGPH----------- :
AtACO2    : VLPSMFKSSYETITEGNPLWNELSAP--SSTLYSWDPNSTYIHEPPYFKNMTANPPGPR----------- :
AtACO3    : VLPDMFRATYESITKGNPMWNKLSVP--ENTLYSWDPNSTYIHEPPYFKDMTMDPPGPH----------- :
AtACO4    : ---------------------------------------------------------------------- :
OsACO1    : VLPDMFKSTYEAITKGNPMWNELSVS--ASTLYPWDPTSTYIHEPPYFKDMTMSPPGPR----------- :
OsACO2    : VLPHMFTQTYESIKRCNRRWNELRVPGEAAALYPWDPSSTYIRKPPYLEGMAMSPPSRPR---------- :
OsACO3    : VLPDMFKSTYEAITKGNPMWNQLTVP--EASLYSWDPNSTYIHEPPYFKDMTMSPPGPH----------- :
OsACO4    : ---------------------------------------------------------------------- :
GmACO1    : VLPEMFRSTYEAITKGNPMWNQLQVP--ADTLYSWDPDSTYIHEPPYFKSMTMDPPGPH----------- :
GmACO2    : VLPDMFRSTYEAITKGNTMWNQLQVP--AETLYSWDPKSTYIHEPPYFKGMTMDPPGAH----------- :
GmACO3    : ---------------------------------------------------------------------- :
GmACO4    : ---------------------------------------------------------------------- :
GmACO5    : SLTTQHRVLVMDVRIRDRAKRRSPMVAPRIKWWHLKGEKQGIFQQKIWEGWCGQSQGSANDLWNKMSQEI :
GmACO6    : ---------------------------------------------------------------------- :
GmACO7    : ---------------------------------------------------------------------- :
GmACO8    : ---------------------------------------------------------------------- :
TaACO1    : ---------------------------------------------------------------------- :
TaACO2    : VLPKMFVDTYDSITEGNDAWNKLVVP--KETLYPWDPKSTYIHKPAYLENITMTPPGPP----------- :
TaACO3    : VLPKMFVDTYGSITEGNDAWNNLVVP--KETLYPWDPKSTYIHKPAYLENITMTPPGPP----------- :
TaACO4    : VLPKMFVDTYGSITEGNDAWNNLVVP--KETLYPWDPKSTYIHKPAYLENITMTPPGPP----------- :
TaACO5    : VLPKMFVDTYGSITEGNDAWNNLVVP--KETLYPWDPKSTYIHKPAYLENITMTPPGPP----------- :
TaACO6    : VLPDMFKGTYEAITKGNPMWNELPVS--ASTLYPWDPKSTYIHEPPYFKDMTMTPPGAR----------- :
TaACO7    : VLPDMFKGTYEAITKGNPMWNELPVS--ASTLYPWDPKSTYIHEPPYFKDMTMTPPGAR----------- :
TaACO8    : ---------------------------------------------------------------------- :
TaACO9    : VLPDMFKSTYEAITKGNPMWNQLPVP--EASLYSWDSNSTYIHEPPYFKDMTMSPPGPH----------- :
TaACO10   : ---------------------------------------------------------------------- :
TaACO11   : VLPDMFKSTYEAITKGNPMWNELPVP--EASLYSWDSNSSYIHEPPYFKDMTMSPPGPH----------- :
TaACO12   : ---------------------------------------------------------------------- :
TaACO13   : VLPDMFKSTYEAITKGNPMWNELPVP--EASLYSWDSNSTYIHEPPYFKDMTMSPPGPH----------- :
TaACO14   : VLVDMFKSTYEVITKGNPMWNQLVVP--TTDVYSWDPNSTYIREPPFFKGMSMDLPGPH----------- :
TaACO15   : VLVDMFKSTYEAITKGNPMWNQLVVP--TADVYSWDPNSTYIREPPFFKGMSMDPPGPH----------- :
PpACO1    : VLPDMFTSTYQAITKGNQTWNSLPAP--SGSQYAWDSKSTYVHEPPFFQNMPKAPPGGK----------- :
PpACO2    : VLPEMFQETYQTITQGNTMWNGLDVP--AGAQYAWDPNSTYVHEPPFFKTMSKDPPGGM----------- :
PpACO3    : VLPEMFQETYQTITQGNTMWNGLDVP--AGAQYAWDPNSTYVHEPPFFKTMSKDPPGGM----------- :
PpACO4    : ---------------------------------------------------------------------- :
PpACO5    : VLPDMFRSTYKAITEGNTMWNKLEAP--AGSQYAWDPKSTYVHDPPFFKTMTKDPPGGR----------- :
SdACO1    : VVPDMFRSTYKTITKENKMWNNLSAP--SGALYAWDPESTYVHDPPFFKSMTESPPGVH----------- :
SdACO2    : VVPDMFRSTYKTITKENKMWNNLSAP--SGALYAWDPESTYVHDPPFFKSMTKSPPGVH----------- :
SdACO3    : ---------------------------------------------------------------------- :
SdACO4    : ---------------------------------------------------------------------- :
SdACO5    : ---------------------------------------------------------------------- :
SdACO6    : VLPEMFKSTYESITKGNTMWNDLPAP--TGDLYSWDPKSTYIHEPPFFKTMTRDPPGVH----------- :
SdACO7    : VLPEMFKSTYESITKGNTMWNDLPAP--TGDLYSWDPKSTYIHEPPFFKTMTRDPPGVH----------- :
```

```
                     *        1000          *        1020          *        1040           *
AtACO1   : --------GVKDAYCLLNFGDSITTDHISPAGSIHKDSPAAKYLMERGVDR-RDFNSYGSRRGNDEIMAR :
AtACO2   : --------EVKDAYCLLNFGDSVTTDHISPAGNIQKTSPAAKFLMDRGVIS-EDFNSYGSRRGNDEVMAR :
AtACO3   : --------NVKDAYCLLNFGDSITTDHISPAGNIQKDSPAAKFLMERGVDR-KDFNSYGSRRGNDEIMAR :
AtACO4   : ---------------------------------------------------------------------- :
OsACO1   : --------PVKDAYCLLNFGDSITTDHISPAGSIHPDSPAARYLKERGVER-KDFNSYGSRRGNDEIMAR :
OsACO2   : --------SVRDAYCLLNLGDSVTTDHISYSGSITPGSAAAEYLRAAGVADRERLGSYGGRRGNDEVVVR :
OsACO3   : --------GVKNAYCLLNFGDSITTDHISPAGSIHKDSPAAKYLLERGVDR-KDFNSYGSRRGNDEVMAR :
OsACO4   : ---------------------------------------------------------------------- :
GmACO1   : --------GVKDAYCLLNFGDSITTDHISPAGSIHKDSPAAKYLVEHGVER-KDFNSYGSRRGNDEVMAR :
GmACO2   : --------GVKDAYCLLNFGDSITTDHISPAGNINKDSPAAKYLLDRGVEQ-KDFNSYGSRRGNDEVMAR :
GmACO3   : ---------------------------------------------------------------------- :
GmACO4   : ---------------------------------------------------------------------- :
GmACO5   : IKVAKETLGESRGFGPRGKESWWWNESVQSKVRVKKECFKEWSRCRNSETWDKYKIARNETKKAVSEARA :
GmACO6   : ---------------------------------------------------------------------- :
GmACO7   : ---------------------------------------------------------------------- :
GmACO8   : ---------------------------------------------------------------------- :
TaACO1   : ---------------------------------------------------------------------- :
TaACO2   : --------SVKDAYCLLSLGDSITTDHISPSGVIKPGTPAAKYLLERGVKP-ENFTTYGSRRANDEIVVR :
TaACO3   : --------SVKDAYCLLSLGDNITTDHISPSGVIKPGTPAAKYLLERGVKP-ENFTTYGSRRANDEIVVR :
TaACO4   : --------SVKDAYCLLSLGDSITTDHISPSGVIKPGTPAAKYLLERGVKP-ENFTTYGSRRANDEIVVR :
TaACO5   : --------SVKDAYCLLSLGDSITTDHISPSGVIKPGTPAAKYLLERGVKP-ENFTTYGSRRANDEIVVR :
TaACO6   : --------PVKDAYCLLNFGDSITTDHISPAGSIHPDSPAAKYLKERNVER-KDFNSYGSRRGNDEIMAR :
TaACO7   : --------PVKDAYCLLNFGDSITTDHISPAGSIHPDSPAAKYLKERNVER-KDFNSYGSRRGNDEIMAR :
TaACO8   : ---------------------------------------------------------------------- :
TaACO9   : --------AVKNAYCLLNFGDSITTDHISPAGSIHRDSPAAKYLLERGVDR-KDFNSYGSRRGNDEVMAR :
TaACO10  : ---------------------------------------------------------------------- :
TaACO11  : --------AVKNAYCLLNFGDSITTDHISPAGSIHRDSPAAKYLLERGVDR-KDFNSYGSRRGNDEVMAR :
TaACO12  : ---------------------------------------------------------------------- :
TaACO13  : --------AVKNAYCLLNFGDSITTDHISPAGSIHRDSPAAKYLLERGVDR-KDFNSYGSRRGNDEVMAR :
TaACO14  : --------SIKDAYCLLSFGDCVTADHISPAGSIHKDSPAAKYLVGHSVKH-RDFNSYGSRRGNYEVMMR :
TaACO15  : --------SIKDAYCLLCFGDCVTTDHISPAGSIHKDSPAAKYLVDHSVKP-GDFNSYGSRRGNYEVMMR :
PpACO1   : --------PVKAAYCLLNFGDSITTDHISPAGNINKDSPAAKFLMDRGVQK-KDFNSYGSRRGNDEIMAR :
PpACO2   : --------SVKDAFCLLNFGDSITTDHISPAGNINKDSPAAKYLMDRGVER-KDFNSYGSRRGNDEIMVR :
PpACO3   : --------SVKDAFCLLNFGDSITTDHISPAGNINKDSPAAKYLMDRGVER-KDFNSYGSRRGNDEIMVR :
PpACO4   : ---------------------------------------------------------------------- :
PpACO5   : --------SVKDAYCLLNFGDSITTDHISPAGNINKDSPAARYLMERGVDR-RDFNSYGSRRGNDEIMGR :
SdACO1   : --------GVKDAYCILNFGDSITTDHISPAGNINKDSPAARYLMERGVEK-KDFNSYGSRRGNDEIMAR :
SdACO2   : --------GVKDAYCILNFGDSITTDHISPAGNINKDSPAARYLMERGVEK-KDFNSYGSRRGNDEIMAR :
SdACO3   : ---------------------------------------------------------------------- :
SdACO4   : ---------------------------------------------------------------------- :
SdACO5   : ---------------------------------------------------------------------- :
SdACO6   : --------GVHDAAVLLNLGDSITTDHISPAGSIHKDSPAARYLTERGVER-KDFNSYGSRRGNDEVMVR :
SdACO7   : --------GVHDAAVLLNLGDSITTDHISPAGSIHKDSPAARYLMERGVER-KDFNSYGSRRGNDEVMVR :
```

```
                    1060         *        1080         *        1100         *        1120
AtACO1   : GTFANIRIVNKHLKGEVGPKTVHIPTGEKLSVFDAAMKYRNEGRDTII--------------------LA :
AtACO2   : GTFANIRIVNKLLKGEVGPNTVHIPTGEKLSVFDAASKYKTAEQDTII--------------------LA :
AtACO3   : GTFANIRIVNKLMNGEVGPKTVHIPSGEKLSVFDAAMRYKSSGEDTII--------------------LA :
AtACO4   : ---------------------------------------------------------------------- :
OsACO1   : GTFANIRLVNKFLKGEVGPKTIHIPSGEKLSVFDAATKYKNEGHDTII--------------------LA :
OsACO2   : GAFANARIVNKLMNGKVGPKTVHVPTGEELCVFDAAIKYKSEGHNMVIV-------------------IA :
OsACO3   : GTFANIRIVNKFLNGEVGPKTVHVPTGEKLYVFDAALKYKSEGHDTIV--------------------LA :
OsACO4   : ---------------------------------------------------------------------- :
GmACO1   : GTFANIRLVNKLLNGEVGPKTIHIPTGEKLYVFDAATRFRE----------------------------- :
GmACO2   : GTFANIRLVNKLLNGEVGPKTVHIPTGEKLYVFDAAQRYKAEGQDTIV--------------------LA :
GmACO3   : ---------------------------------------------------------------------- :
GmACO4   : ---------------------------------------------------------------------- :
GmACO5   : QAFDGLYQALGTRDGERSIYRLAKGRERKTRDLDQVKCVKDEEGKVLVHEKDIKERWKAYFHNLFNDGYG :
GmACO6   : ---------------------------------------------------------------------- :
GmACO7   : ---------------------------------------------------------------------- :
GmACO8   : ---------------------------------------------------------------------- :
TaACO1   : ---------------------------------------------------------------------- :
TaACO2   : GAFANIRIVNKLLEGEVGPKTVHVPTGEKHYVFDAAMKYKSEGHDMVI--------------------LA :
TaACO3   : GAFANIRIVNKLLEGEVGPKTIHVPTGEKHYVFDAAMKYKSEGHDMVI--------------------LA :
TaACO4   : GAFANIRIVNKLLEGEVGPKTIHVPTGEKHYVFDAAMKYKSEGHDMVI--------------------LA :
TaACO5   : GAFANIRIVNKLLEGEVGPKTVHVPTGEKHYVFDAAMKYKSEGHDMVI--------------------LA :
TaACO6   : GTFANIRIVNKFLKGEVGPKTVHVPSGEKLAVFDAAMKYKNEGHDTII--------------------LA :
TaACO7   : GTFANIRIVNKFLKGEVGPQTIHVPSGEKLAVFDAAMKYKNEGHDTII--------------------LA :
TaACO8   : ---------------------------------------------------------------------- :
TaACO9   : GTFANIRIVNKFLGGEVGPKTIHVPTGEKLSVFDAATKYKSEGHDTII--------------------LA :
TaACO10  : ---------------------------------------------------------------------- :
TaACO11  : GTFANIRIVNKFLGGEVGPKTIHVPTGEKLSVFDAATKYKSEGHDTII--------------------LA :
TaACO12  : ---------------------------------------------------------------------- :
TaACO13  : GTFANIRIVNKFLGGEVGPKTIHVPTGEKLSVFDAATKYKSEGHDTII--------------------LA :
TaACO14  : GTFGNIRIVNKLLDGEPGPKTIHIPTREKLYVYDAAMRYKNDGQDTIV--------------------LV :
TaACO15  : GTFGNIRIVNKLLDGEPGPKTIHVPTREKLYVYDAAMRYKNDGQDTIV--------------------LA :
PpACO1   : GTFANIRIVNKFLGGEVGPKTIHVPSKERLFIFDAAKKYKDEGHDTII--------------------LA :
PpACO2   : GTFANIRIVNKFLKGEVGPKTVHVPTQEKMFIYDAAMKYKEEGHDTII--------------------LA :
PpACO3   : GTFANIRIVNKFLKGEVGPKTVHVPTQEKMFIYDAAMKYKEEGHDTII--------------------LA :
PpACO4   : ---------------------------------------------------------------------- :
PpACO5   : GTFANIRIVNKFLKGEVGPKTLHVPTQEKMFIYDAAQKYKAEGHDTII--------------------LA :
SdACO1   : GTFANIRIVNKFLKGEVGPKTIHIPSGEKLSVFDAAKKYQDEGHDTII--------------------LA :
SdACO2   : GTFANIRIVNKFLKGEVGPKTIHIPSGEKLSVFDAAKKYQDEGHDTII--------------------LA :
SdACO3   : ---------------------------------------------------------------------- :
SdACO4   : ---------------------------------------------------------------------- :
SdACO5   : ---------------------------------------------------------------------- :
SdACO6   : GTFANIRIVNKLLKGEVGPKTVHLPSGECHWVFDVAQKYKDEGKEMVV--------------------LA :
SdACO7   : GTFANIRIVNKLLKGEVGPKTVHLPSGECHWVFDVAQKYKDEGKEMVV--------------------LA :
```

```
                      *        1140         *        1160         *        1180         *
AtACO1   : GAEYGSGSSRDWAAKGPMLLGVKAVISKSFERIHRSNLVGMGIIPLCFKAGEDAETLG------------ :
AtACO2   : GAEYGSGSSRDWAAKGPLLLGVKAVIAKSFERIHRSNLAGMGIIPLCFKAGEDAETLG------------ :
AtACO3   : GAEYGSGSSRDWAAKGPMLQGVKAVIAKSFERIHRSNLVGMGIIPLCFKSGEDADTLG------------ :
AtACO4   : ---------------------------------------------------------------------- :
OsACO1   : GAEYGSGSSRDWAAKGPMLQGVKAVIAKSFERIHRSNLAGMGIIPLCFKSGEDADTLG------------ :
OsACO2   : GAEYGSGSSRDSAAKGPMLLMI------------------------------------------------ :
OsACO3   : GAEYGSGSSRDWAAKGPMLLGVKAVIAKSFERIHRSNLVGMGIIPLCFKAGEDADSLG------------ :
OsACO4   : ---------------------------------------------------------------------- :
GmACO1   : -CLYDKISSKCYICN--ISVYFPLCINYRVLFVQIDNTKGM----------------------------- :
GmACO2   : GAEYGSGSSRDWAAKGPMLLGVKAVIAKSFERIHRSNLVGMGIVPLCFKSGEDADTLG------------ :
GmACO3   : ---------------------------------------------------------------------- :
GmACO4   : ---------------------------------------------------------------------- :
GmACO5   : YDSSSLDTREEDRNYKYYRRIQKQEVKEALKRMSNGKAVGPDNIPIEVWKTLGDRGLEWLTKLFNEIMRS :
GmACO6   : ---------------------------------------------------------------------- :
GmACO7   : ---------------------------------------------------------------------- :
GmACO8   : ---------------------------------------------------------------------- :
TaACO1   : ---------------------------------------------------------------------- :
TaACO2   : GDEYGAGSSRDSAAKGPLLLGVKAVIAKGFERIHRSNLVGMGIIPLRFKAGEDADSLN------------ :
TaACO3   : DDEYGAGSSRDSAAKGPLLLGVKAVIAKGFERIHRSNLVGMGIIPLRFKVGEDADSLN------------ :
TaACO4   : GDEYGAGSSRDSAAKGPLLLGVKAVIAKGFERIHRSNLVGMGIIPLRFKVGEDADSLN------------ :
TaACO5   : GDEYGAGSSRDSAAKGPLLLGVKAVIAKGFERIHRSNLVGMGIIPLRFKAGEDADSLN------------ :
TaACO6   : GAEYGSGSSRDWAAKGPMLQGVKAVIAKSFERIHRSNLAGMGIVPLCFKAGEDADSLG------------ :
TaACO7   : GAEYGSGSSRDWAAKGPMLQGVKAVIAKSFERIHRSNLAGMGIVPLCFKAGEDADTLG------------ :
TaACO8   : ---------------------------------------------------------------------- :
TaACO9   : GAEYGSGSSRDWAAKGPMLLGVKAVISKSFERIHRSNLVGMGIIPLCFKAGEDADSLG------------ :
TaACO10  : ---------------------------------------------------------------------- :
TaACO11  : GAEYGSGSSRDWAAKGPMLLGVKAVIAKSFERIHRSNLVGMGIIPMCFKAGEDADSLG------------ :
TaACO12  : ---------------------------------------------------------------------- :
TaACO13  : GAEYGSGSSRDWAAKGPMLLGVKAVISKSFERIHRSNLVGMGIIPLCFKAGEDADSLG------------ :
TaACO14  : GSKYGTGSSRDWDAKGTMLLGVKAVIAKSFERIHRSNLVGMGVIPLCFKSGEDMDSIG------------ :
TaACO15  : GSEYGTGSSRDWDAKRTMLLGVKAVIAKSSERIH------------------------------------ :
PpACO1   : GAEYGSGSSRDWAAKGPYLQGVKAVISKSFERIHRSNLVGMGLIPLCFKQGQDADSLG------------ :
PpACO2   : GAEYGSGSSRDWAAKGPYLQGVKAVIAKSFERIHRSNLVGMGLIPLCFKEGEDAETLG------------ :
PpACO3   : GAEYGSGSSRDWAAKGPYMQGVKAVIAKSFERIHRSNLVGMGLIPLCFKEGEDAETLG------------ :
PpACO4   : ---------------------------------------------------------------------- :
PpACO5   : GAEYGSGSSRDWAAKGPYLQGVKAVIAKSFERIHRSNLVGMGLIPLCFRNGEDADSLG------------ :
SdACO1   : GAEYGSGSSRDWAAKGPYLQGVKAVIAKSFERIHRSNLVGMGIIPLCFKSGEDAESLG------------ :
SdACO2   : GAEYGSGSSRDWAAKGPYLQGVKAVIAKSFERIHRSNLVGMGIIPLCFKSGEDAESLG------------ :
SdACO3   : ---------------------------------------------------------------------- :
SdACO4   : ---------------------------------------------------------------------- :
SdACO5   : ---------------------------------------------------------------------- :
SdACO6   : GAEYGSGSSRDWAAKGPFLQGVKAVISKSFERIHRSNLVGMSIIPLCYKNGEDAESLG------------ :
SdACO7   : GAEYGSGSSRDWAAKGPFLQGVKAVISKSFERIHRSNLVGMSIIPLCYKNGEDAESLG------------ :
```

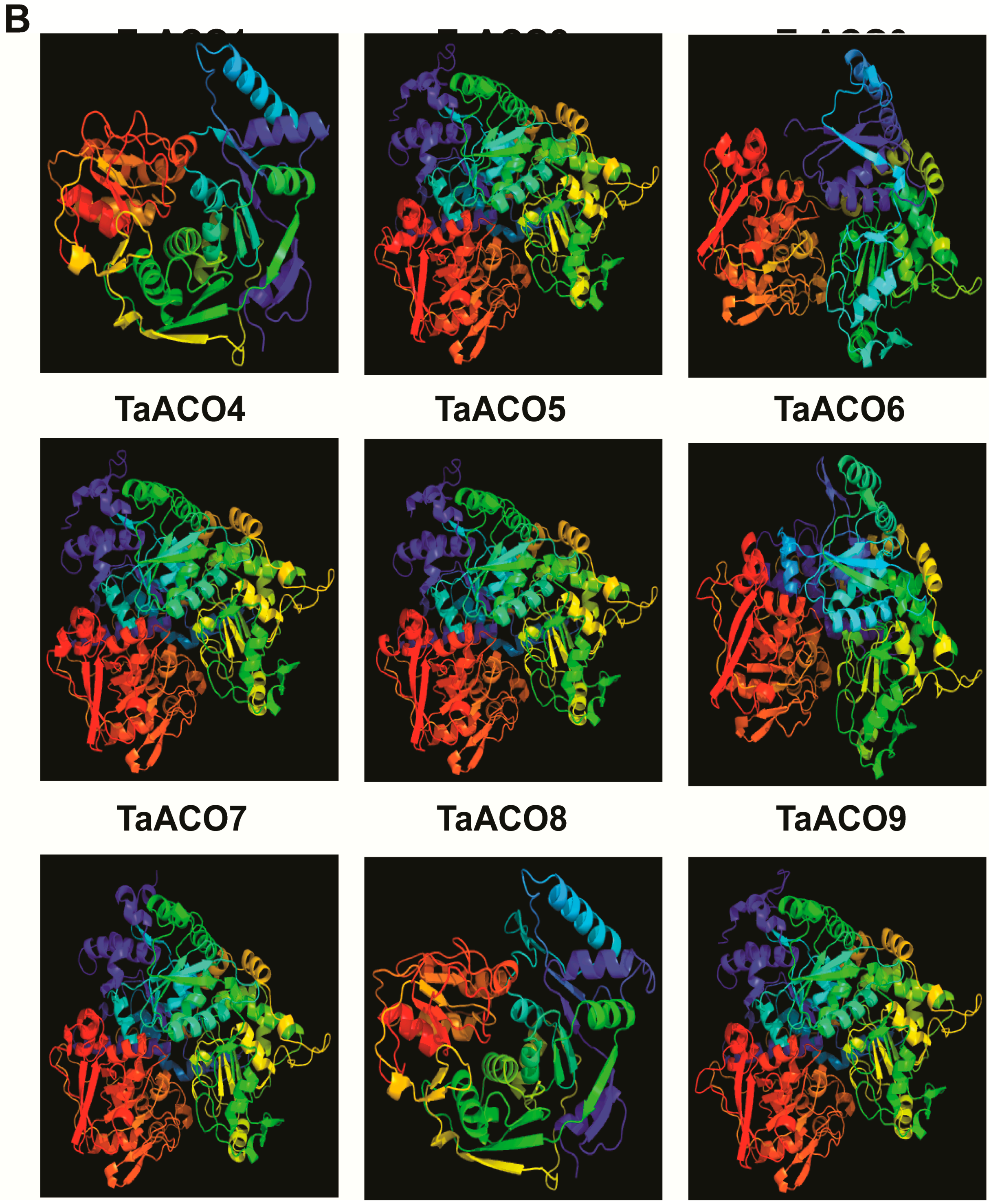
B
TaACO4
TaACO5
TaACO6
TaACO7
TaACO8
TaACO9

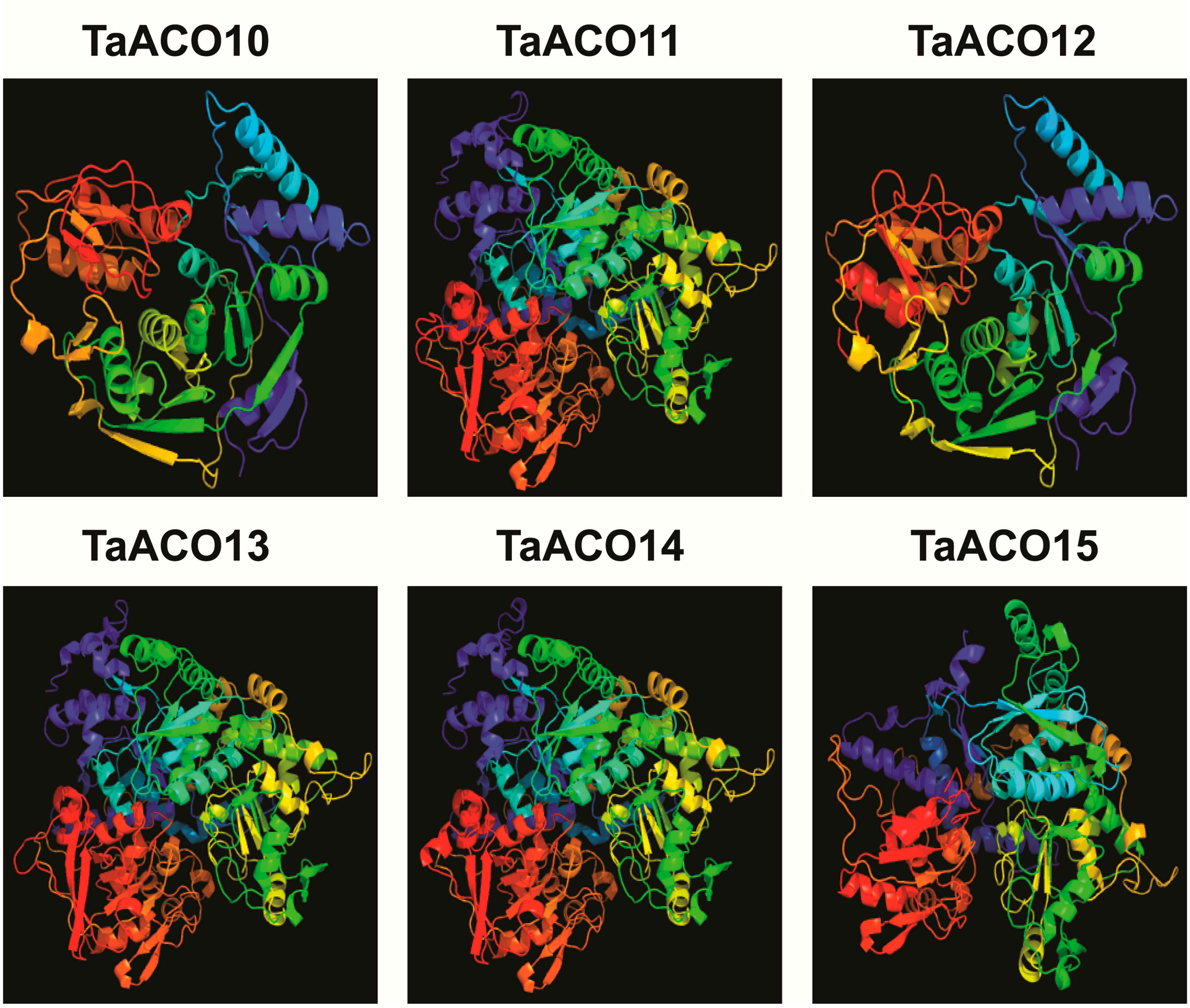

Figure S8. Alignment and 3-dimensional structure of the TaACO protein sequences. **A.** The aconitase domain is underlined with red color. Colored and shaded amino acids are chemically similar residues. Dashes indicate gaps introduced to maximize the alignment of the homologous region. **B.** Predicted 3D structures TaACO proteins.

A

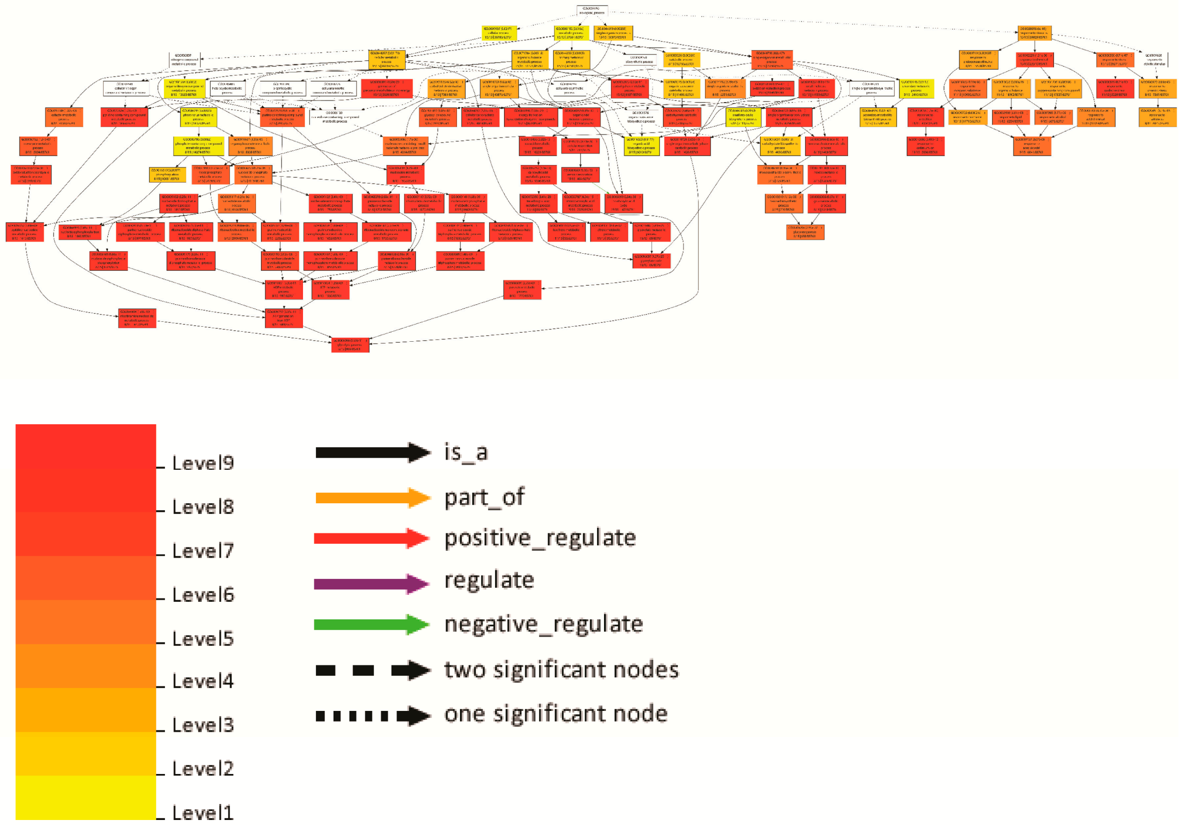
Level9
Level8
Level7
Level6
Level5
Level4
Level3
Level2
Level1
is_a
part_of
positive_regulate
regulate
negative_regulate
two significant nodes
one significant node

B

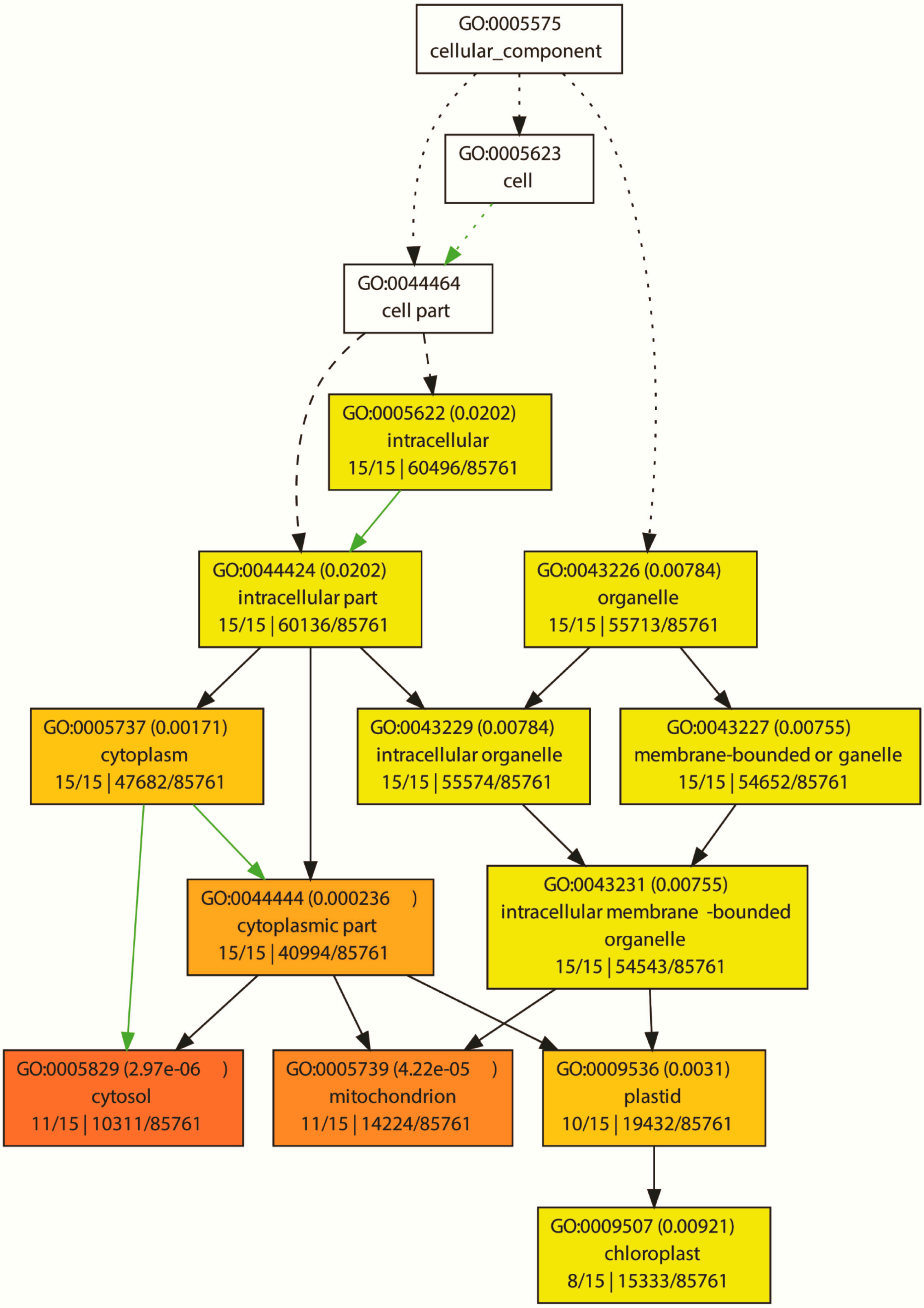

GO:0005575
cellular_component
GO:0005623
cell
GO:0044464
cell part
GO:0005622 (0.0202)
intracellular
15/15 | 60496/85761
GO:0044424 (0.0202)
intracellular part
15/15 | 60136/85761
GO:0043226 (0.00784)
organelle
15/15 | 55713/85761
GO:0005737 (0.00171)
cytoplasm
15/15 | 47682/85761
GO:0043229 (0.00784)
intracellular organelle
15/15 | 55574/85761
GO:0043227 (0.00755)
membrane-bounded organelle
15/15 | 54652/85761
GO:0044444 (0.000236)
cytoplasmic part
15/15 | 40994/85761
GO:0043231 (0.00755)
intracellular membrane -bounded
organelle
15/15 | 54543/85761
GO:0005829 (2.97e-06)
cytosol
11/15 | 10311/85761
GO:0005739 (4.22e-05)
mitochondrion
11/15 | 14224/85761
GO:0009536 (0.0031)
plastid
10/15 | 19432/85761
GO:0009507 (0.00921)
chloroplast
8/15 | 15333/85761

C

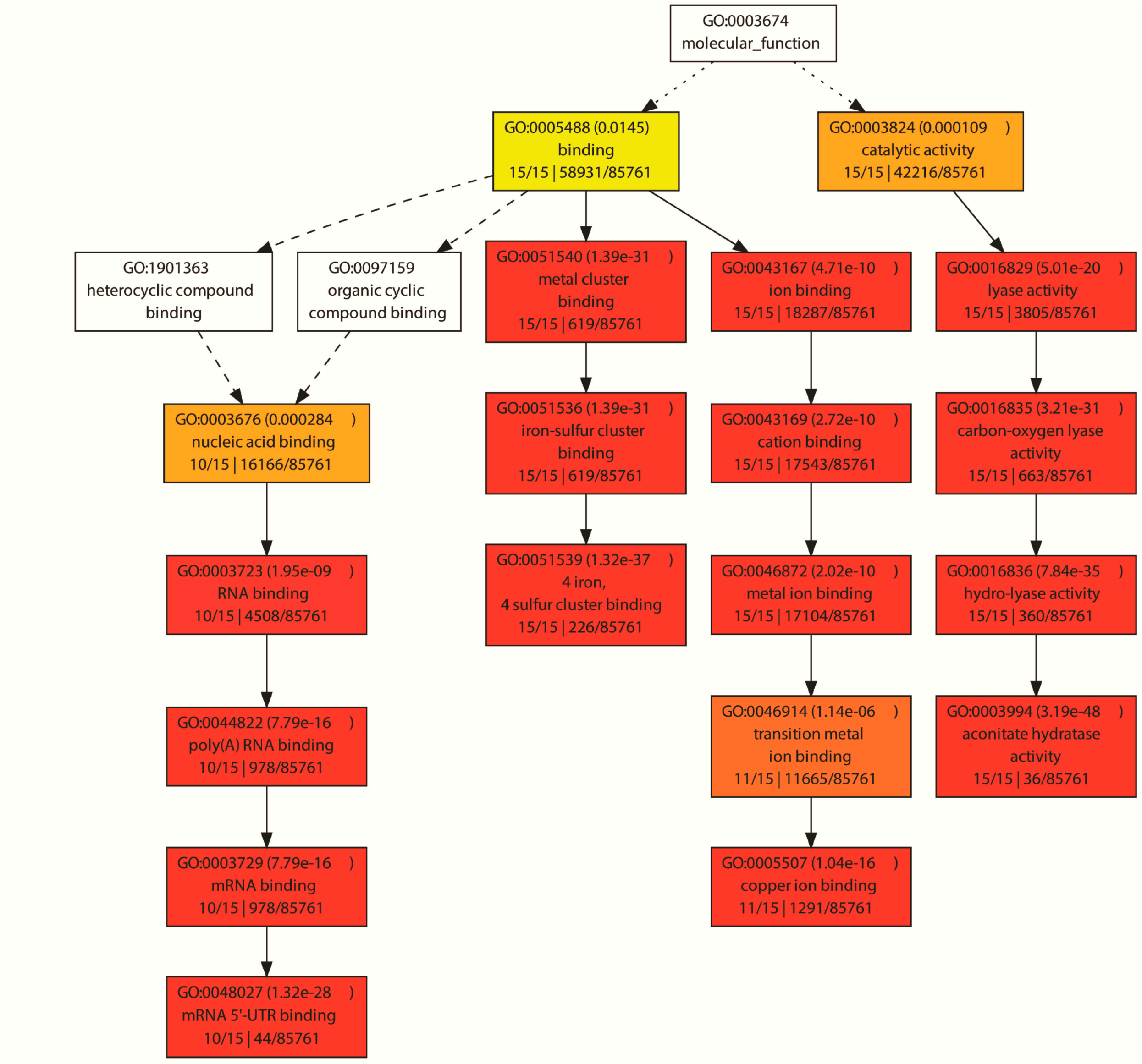

Figure S9. The predicted GO term of TaACO gene family using AgriGO **A.** Biological Process. **B.** Cellular component. **C.** Molecular function.

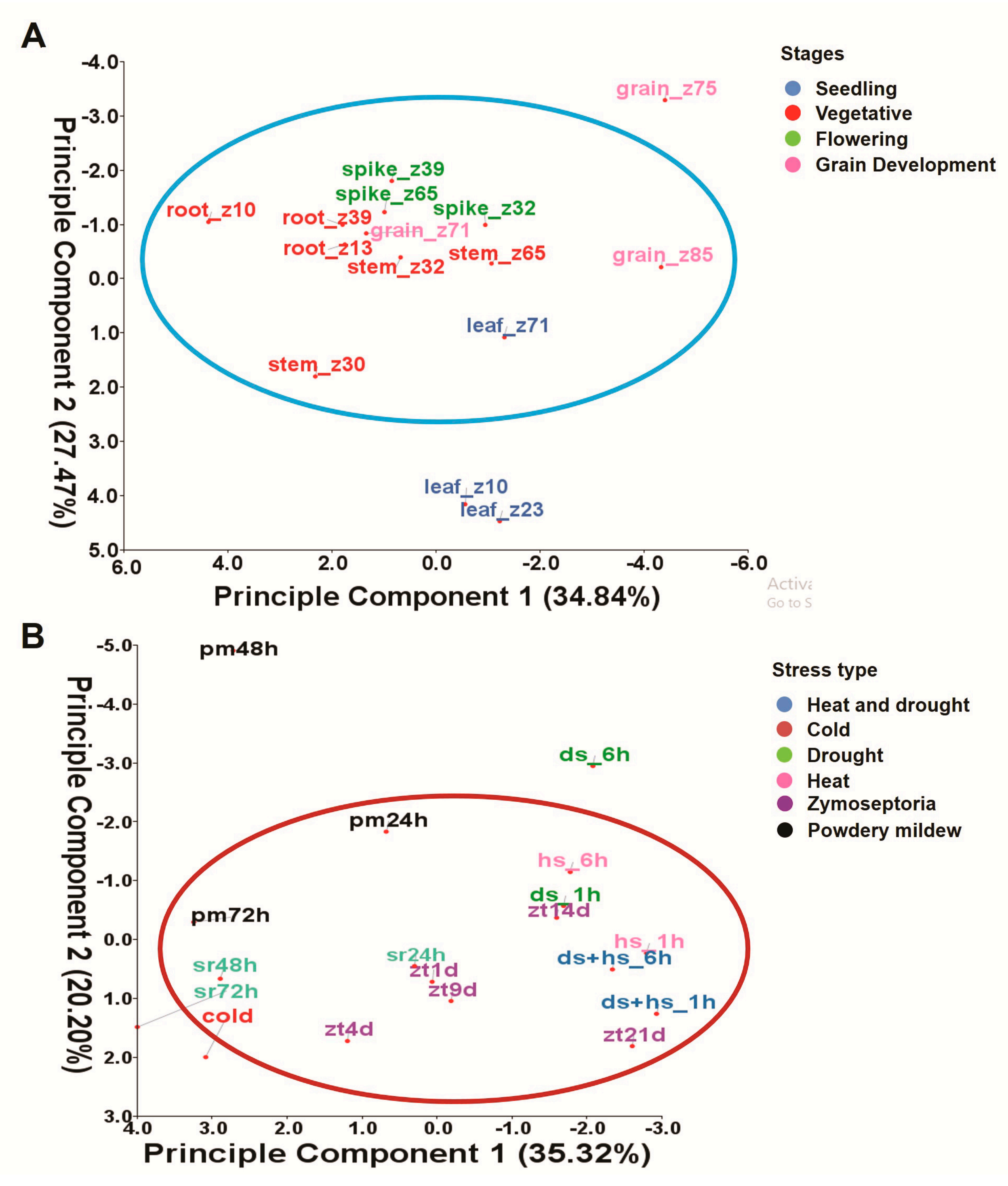

Figure S10. PCA plots displaying grouping of different (A) Different tissues and developmental stages (B) Diverse stress conditions based on the TaACO expression profiles. HS: Heat stress, DS: Drought stress, DS+HS: Combined drought and heat stress; PM: Powdery mildew; SR: Stripe rust, Zt: *Zymoseptoria tritici*, d: days and h: hour.

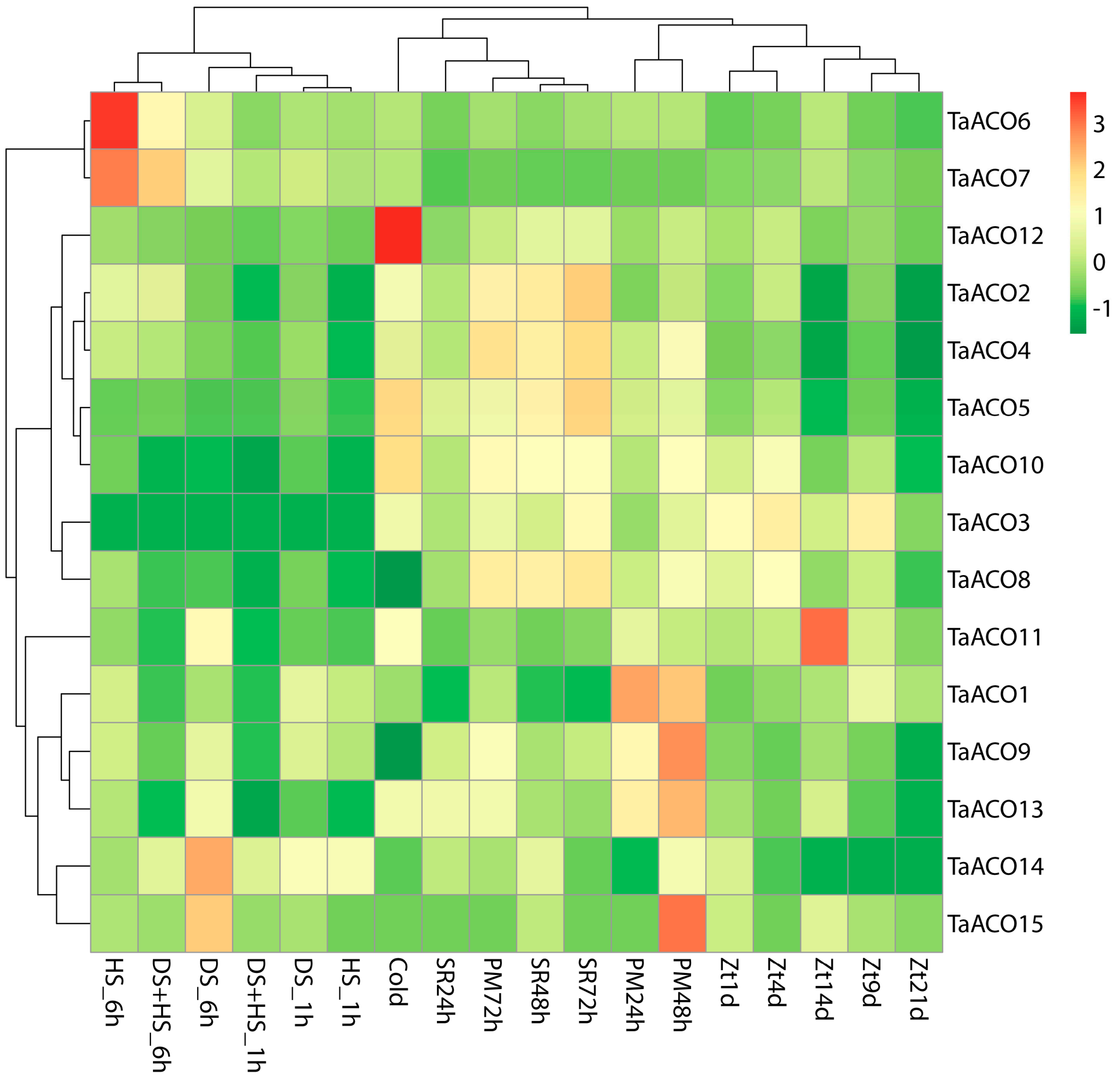

Figure S11. Heatmaps signifying the expression profile of *TaACO* genes in biotic and abiotic stress conditions. TPM values were directly used to produce the heatmaps. HS: Heat stress, DS: Drought stress, DS+HS: Combined drought and heat stress; PM: Powdery mildew; SR: Stripe rust, Zt: *Zymoseptoria tritici*, d: days and h: hour.
